# Box, stalked, and upside-down? Draft genomes from diverse jellyfish (Cnidaria, Acraspeda) lineages: *Alatina alata* (Cubozoa), *Calvadosia cruxmelitensis* (Staurozoa), and *Cassiopea xamachana* (Scyphozoa)

**DOI:** 10.1093/gigascience/giz069

**Published:** 2019-07-01

**Authors:** Aki Ohdera, Cheryl L Ames, Rebecca B Dikow, Ehsan Kayal, Marta Chiodin, Ben Busby, Sean La, Stacy Pirro, Allen G Collins, Mónica Medina, Joseph F Ryan

**Affiliations:** 1 Department of Biology, Pennsylvania State University, 326 Mueller, University Park, PA, 16801, USA; 2 Department of Invertebrate Zoology, National Museum of Natural History, Smithsonian Institution, 10th Street & Constitution Avenue NW, Washington DC, 20560, USA; 3 National Center for Biotechnology Information, 8600 Rockville Pike MSC 3830, Bethesda, MD, 20894, USA; 4 Data Science Lab, Office of the Chief Information Officer, Smithsonian Institution, 10th Street & Constitution Avenue NW, Washington DC, 20560, USA; 5 UPMC, CNRS, FR2424, ABiMS, Station Biologique, Place Georges Teissier, 29680 Roscoff, France; 6 Whitney Laboratory for Marine Bioscience, University of Florida, 9505 Ocean Shore Boulevard, St. Augustine, FL, 32080, USA; 7 Department of Biology, University of Florida, 220 Bartram Hall, Gainesville, FL, 32611, USA; 8 Department of Mathematics, Simon Fraser University, 8888 University Drive, Barnaby, British Columbia, BC, V5A 1S6, Canada; 9 Iridian Genomes, Inc., 6213 Swords Way, Bethesda, MD, 20817, USA; 10 National Systematics Laboratory of NOAA's Fisheries Service, 1315 East-West Highway, Silver Spring, MD, 20910, USA

**Keywords:** staurozoa, scyphozoa, cubozoa, acraspeda, cnidaria, medusozoa

## Abstract

**Background:**

Anthozoa, Endocnidozoa, and Medusozoa are the 3 major clades of Cnidaria. Medusozoa is further divided into 4 clades, Hydrozoa, Staurozoa, Cubozoa, and Scyphozoa—the latter 3 lineages make up the clade Acraspeda. Acraspeda encompasses extraordinary diversity in terms of life history, numerous nuisance species, taxa with complex eyes rivaling other animals, and some of the most venomous organisms on the planet. Genomes have recently become available within Scyphozoa and Cubozoa, but there are currently no published genomes within Staurozoa and Cubozoa.

**Findings:**

Here we present 3 new draft genomes of *Calvadosia cruxmelitensis* (Staurozoa), *Alatina alata* (Cubozoa), and *Cassiopea xamachana* (Scyphozoa) for which we provide a preliminary orthology analysis that includes an inventory of their respective venom-related genes. Additionally, we identify synteny between POU and Hox genes that had previously been reported in a hydrozoan, suggesting this linkage is highly conserved, possibly dating back to at least the last common ancestor of Medusozoa, yet likely independent of vertebrate POU-Hox linkages.

**Conclusions:**

These draft genomes provide a valuable resource for studying the evolutionary history and biology of these extraordinary animals, and for identifying genomic features underlying venom, vision, and life history traits in Acraspeda.

## Introduction

Some of the most fascinating and outstanding mysteries related to the genomic underpinnings of metazoan biology are centered around cnidarians [1]. Active areas of research include the basis of venom evolution and diversification [2–4], mechanisms of independent evolution of image-forming vision (lens eyes) [5–7], and the emergence of a pelagic adult stage within a biphasic (or multiphasic) life cycle [8]. Cnidaria encompasses 3 major clades: Anthozoa, Endocnidozoa, and Medusozoa [9–12]. Anthozoa comprises Hexacorallia and Octocorallia. Hexacorallia includes scleractinian corals, anemones, and zooanthids and is characterized by a 6-fold symmetry, with species exhibiting both colonial and solitary forms. Octocorallia includes sea fans, gorgonians, and soft corals; these animals are characterized by pinnate tentacles in 8-fold symmetry. Endocnidozoa, comprising the parasitic lineages Myxozoa and Polypodiozoa, was only recently properly classified as Cnidaria [13–15]. Medusozoans are characterized by the emergence of a medusa life history stage within some taxa of the clade, their high diversity in regards to life history and morphology, the presence of a linear mitochondrial genome (with a variable number of chromosomes), and by the presence of a hinged cap at the apex of the cnidocyst (cnidarian stinging organelles) [8, 16, 17].

**Figure 1:**
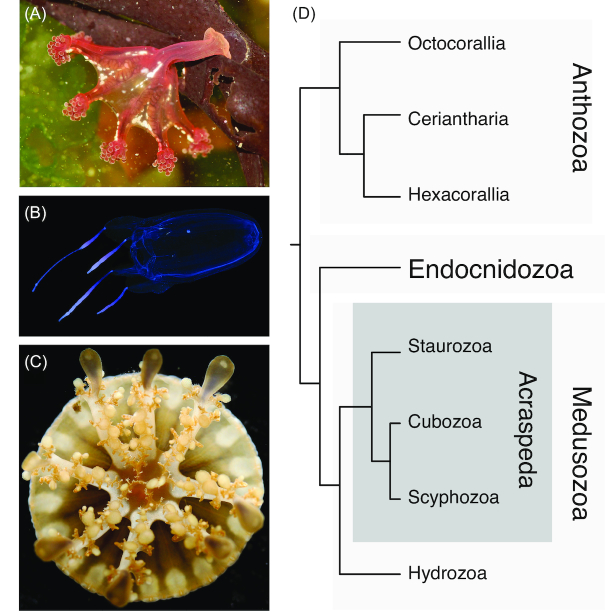
A, *Calvadosia cruxmelitensis* (Staurozoa); B, *Alatina alata* (Cubozoa); and C, *Cassiopea xamachana* (Scyphozoa). D, Phylogenetic relationship of major cnidarian lineages after Kayal et al. [11], revealing Cubozoa and Scyphozoa as sister groups, united with Staurozoa to form Acraspeda.

There are ∼3,900 described species within Medusozoa, classified into 4 diverse lineages: Hydrozoa (hydroids, hydromedusae, siphonophores), Staurozoa (stalked jellyfish), Cubozoa (box jellyfish), and Scyphozoa (true jellyfish) (Fig. 1A−C) [1, 11]. There exists much debate regarding the phylogenetic relationships among these lineages [10, 16, 18–20]. Recent phylogenomic analyses have placed Staurozoa as the sister to a clade that contains Cubozoa and Scyphozoa, reuniting these lineages into a group called Acraspeda (Fig. 1D) [9, 15, 21]. Given the extensive morphological diversity within Cnidaria, understanding the evolutionary relationships and mechanisms leading to lineage-specific innovations has been of considerable interest but has been fraught with challenges. In particular, the evolution and subsequent loss of the medusoid form in some lineages hints at a complex evolutionary history within Medusozoa [22].

The mechanisms of medusa formation are variable amongst medusozoans, often involving 2 phenotypically distinct life stages—polyp and medusa—that are genotypically identical (reviewed by Lewis Ames [1]). Cubozoan polyps undergo partial or complete metamorphosis and develop into the adult medusoid form capable of sexual reproduction, although in some cases a polyp rudiment remains [23]. Scyphozoan polyps (scyphistomae) undergo a transition known as strobilation, in which the upper calyx proceeds through metamorphosis and transverse fission to produce a medusa [24]. Unlike other medusozoans, staurozoans lack a free-swimming medusa stage but exhibit medusa-associated characters that are present in other medusozoans [25]. The basal portion of the adult forms a stalk, or peduncle, while coronal muscles and gastric filaments, among other features, characterize the apical portion (calyx) of the adult [20, 25–27]. Hydrozoans exhibit the greatest variation in life history strategies and often lack a medusa form [1]. Species that give rise to the medusoid form do so via lateral buds generated by asexual polyps, while others possess sexual polyps without a free-swimming stage [28, 29]. Elsewhere within Cnidaria, Anthozoa and the parasitic Endocnidozoa lack the medusa stage or medusoid characters entirely. Research on medusa development has shown similar gene expression patterns between hydrozoans and scyphozoans, with developmental genes co-opted for patterning the medusa body plan [30, 31]. Interestingly, strobilation in scyphozoans was recently shown to be under the control of the retinoic acid pathway [32–34]; these same genes are involved in metamorphosis of insects and amphibians, hinting towards regulation of metamorphosis being a conserved function in metazoans [35]. The study also found that potential lineage-specific genes were involved in controlling strobilation, suggestive of genomic innovations within Medusozoa playing a role in medusa morphogenesis, or more specifically within Scyphozoa.

Hox genes, which control body formation during early embryonic development, predate the emergence of both Bilateria and Cnidaria, and the evolution of these genes played a crucial role in the diversification of these lineages [36–38]. In particular, clustering and synteny has been shown to be important in bilaterians [39], but also in some cnidarians, such as the anthozoan *Nematostella vectensis* [36, 40, 41], and in several hydrozoan species [42–46]. Other than an initial characterization of select Hox genes in *Cassiopea xamachana* [47], information about Hox genes and Hox gene clustering in Acraspeda species has been limited. In hydrozoans and vertebrates, Hox genes were shown to be linked to another class of homeobox genes, the POU genes [48], but this linkage has not been demonstrated in any other cnidarian lineages. These new Acraspeda genomes provide us with an opportunity to investigate the evolutionary history of the POU-Hox linkage in more detail.

Genomic resources necessary to understand medusozoan evolution have been lacking, with genomes predominantly available for anthozoans and hydrozoans [49–56]. However, 3 new scyphozoan genomes, 2 genomes of the moon jellyfish *Aurelia* spp. and the giant Nomura's jellyfish *Nemopilema nomurai* were recently sequenced [57–59]. In addition, the genome for the cubozoan *Morbakka virulenta* has also recently been released [59]. While the majority of Medusozoa species are represented by hydrozoans (>90%), both cubozoans and scyphozoans garner significant attention as a result of their impact on economy and tourism [1, 60]. Largely due to venom being used as a mechanism of defense and prey capture, the inherent risk of jellyfish sting has been exacerbated by uncertainty about how cnidarians will respond to modern-day anthropogenic disturbances [61, 62]. Despite these risks, relatively little is known about cnidarian venom, as compared to snakes, cone snails, and other venomous organisms. Given the great phylogenetic distance between cnidarians and these well-studied venomous organisms, a better understanding of the cnidarian venom repertoire can provide insight into the evolution of venom and venom-encoding genes.

Here we present 3 new genomes for species of the 3 major Acraspeda lineages: *Calvadosia cruxmelitensis* (formerly *Lucernariopsis cruxmelitensis*) (Staurozoa, NCBI:txid1843192), *Alatina alata* (Cubozoa, NCBI:txid1193083), and *C. xamachana* (Scyphozoa, NCBI:txid12993). The winged box jellyfish *Alatina alata* (Cnidaria: Cubozoa: Carybdeida: Alatinidae) has been of interest due to its unusual circumtropical distribution [63], extraordinarily rapid gonad development [64], and its reputation as a potent stinger, earning it the honor of being the only jellyfish species to have its own category in US weather reports [65]. The stalked jellyfish *C. cruxmelitensis* has been the recent subject of detailed anatomic [20] and biodiversity studies [27]. The upside-down jellyfish *C. xamachana* is an established model for understanding cnidarian-dinoflagellate endosymbiosis [66] and, with its ease of culturing and tractability in the laboratory setting, is poised as a model system for evolutionary developmental biology research and other laboratory-based studies [67].

The genomes and corresponding gene annotations from these 3 lineages will serve as useful resources aimed at sparking investigative research into the evolution and diversification of life history strategies across cnidarians. Furthermore, future studies examining cnidarian venom evolution, and phylogeographic patterns of venomous jellyfish, may provide opportunities for development of jellyfish-derived therapeutic drugs, and additional novel biopharmaceuticals (reviewed by Lewis Ames [1]).

## Data Description

### Genome sampling, sequencing, and assembly

These 3 acraspedan genomes were assembled at different times throughout a 5-year period as part of several independent projects overseen by the coauthors, using separate methods for collection, extraction, sequencing, and assembly (see below). This valuable resource to the scientific community is the culmination of an extensive collaborative effort to respond to the need for model medusozoan systems in a plethora of research fields.

### *Cassiopea xamachana* sample collection and DNA extraction

We propagated *C. xamachana* polyps from a single polyp via asexual budding (Line T1-A). Polyps were maintained symbiont-free at 26°C and fed 3 times weekly with *Artemia* nauplii. To avoid the possibility of food source contaminates interfering with downstream bioinformatic analysis, we starved the polyps for 7 days in antibiotic-treated seawater prior to preservation in 95% ethanol; any *Artemia* cysts retained within the gut were manually removed before preservation. We extracted genomic DNA from the apo-symbiotic (lacking endosymbionts) polyps using a CTAB (cetyl trimethylammonium bromide) phenol chloroform extraction, first performing an overnight digestion of whole polyp tissue with proteinase K (20 mg/L) in CTAB buffer before proceeding with the standard protocol. DNA extract was stored at −20°C until further processing.

### *Calvadosia cruxmelitensis* sample collection and DNA extraction

We collected adult specimens of *C. cruxmelitensis* in January 2013 at Chimney Rock, off the coast of Penzance, Cornwall, England. Specimens were immediately preserved in ethanol and stored at −20°C until further processing. We extracted genomic DNA using a phenol-choloroform protocol in an Autogen mass extractor (Holliston, MA, USA), and stored the DNA extract at −20°C until further processing.

### *Alatina alata* sample collection and DNA extraction

We collected *A. alata* material during a spermcasting aggregation in Bonaire, The Netherlands (April 2014, 22:00–01:00) according to the methods in Lewis Ames et al. [7]. We selected a single live spermcasting male medusa from the same cohort as the female medusa used in previously published RNA sequencing studies (Genbank Accession: GEUJ01000000) [7, 9]. The medusa was divided into 4 longitudinal sections, and one quarter was placed into a 15-mL tube with pure (99%) ethanol, flash-frozen at −180°C (using a dry shipper), and subsequently transported to the Smithsonian National Museum of Natural History and stored at −20°C. We extracted genomic DNA using a DNeasy Blood & Tissue Kit (Qiagen: Hilden, Germany), following the manufacturer's protocol. DNA extract was stored at −20°C until further processing.

### *Cassiopea xamachana* sequencing and assembly

Library construction and sequencing was performed at HudsonAlpha Institute for Biotechnology. Four 350-bp paired-end linear libraries with insert sizes of 500 bp were generated with Illumina TruSeq DNA PCR-Free LT Prep Kits (San Diego, CA, USA) and sequenced on the Illumina Hiseq2000. Approximately 634 million reads totaling 117.6 Gb of high-quality paired-end sequence data were generated. We performed adaptor trimming and quality filtering using Trimmomatic v0.36 [68] with default settings, followed by genome size estimation and error correction with Allpaths-LG version 52,488 [69]. We removed mitochondrial reads using FastqSifter v1.1.1 (RRID:SCR_017200) [70] using the *C. xamachana* mitochondrial genome as a reference (NCBI NC_01 6466.1). We performed *de novo* genome assemblies using ABySS 2.0.1 with default settings [71], SPAdes genome assembler v3.10.0 [72], and Platanus version 1.2.1 (with default parameters, *k* = 89) [73] (Table 1). We used a custom Perl script, plat.pl [74], to invoke the Platanus commands for assembly, scaffolding, and gap closing. Of the 3 assembly methods, Platanus produced the best draft assembly with 93,483 scaffolds measuring a total of 393.5 Mb with an N50 of 15,563 bp (Table 1) (European Nucleotide Archive [ENA] Accession OLMO01000000). We recovered 82.66% (53.63% complete and 29.03% partial) of the core eukaryotic genes and 66.97% (58.59% complete and 8.38% partial) of the core metazoan genes with CEGMA version 2.5 [75] and BUSCO version 2.01 [76], respectively, through the gVolante web server [77] (Table 1).

**Table 1:** Statistics of*Alatina alata, Calvadosia cruxmelitensis*, and *Cassiopea xamachana* genome assemblies

	*Alatina alata*	*Calvadosia cruxmelitensis*	*Cassiopea xamachana*
NCBI Taxa ID	1,193,083	1,843,192	12,993
No. of sequences	291,445	50,999	93,483
Estimated genome size	2,673,604,203	230,957,924	361,689,769
Total length (bp)	851,121,747	209,392,379	393,520,168
N50 (bp)	7,049	16,443	15,563
CEGMA (% complete)	8.06	61.29	53.63
CEGMA (% complete + partial)	29.84	91.94	82.66
BUSCO (% complete)	18.30	70.86	58.59
BUSCO (% complete + partial)	32.11	85.07	66.97
Guanine-cytosine content (%)	38.07	39.95	37.07
Assembly accession	PUGI00000000	OFHS01000000	OLMO01000000
NCBI raw read accession	PRJNA421156	PRJEB23739	PRJEB23739
Specimen Voucher ID	USNM 1,248,604	USNM 1,286,381	UF Cnidaria 12,979

### *Calvadosia cruxmelitensis* sequencing and assembly

Library construction and sequencing for *C. cruxmelitensis* were performed at the University of Florida Interdisciplinary Center for Biotechnology Research. Four 150-bp paired-end linear libraries and four 150-bp single-end linear libraries with insert size of 300 bp were generated and sequenced on the Illumina NextSeq 500, generating 291,944,064 paired-end reads and 141,911,072 single-end reads. We performed adaptor trimming and quality filtering using Trimmomatic-0.32 [68] with default settings, followed by genome size estimation error correction using Allpaths-LG v.44837 [69]. We removed mitochondrial sequences to improve the final assembly with FastqSifter v1.1.1 (RRID:SCR_017200) [70], using a *de novo* assembly of the *C. cruxmelitensis* mitochondrial genome. We assembled the *C. cruxmelitensis* mitochondrial genome by capturing contigs from an initial assembly using available staurozoan mitochondrial DNA sequences from NCBI as a reference, following the methods presented in Kayal et al. [78], using Geneious v9.0 to generate the final mitochondrial assembly. We checked completeness of the mitochondrial genome using NCBI BLAST against the non-redundant database in addition to a manually generated set of medusozoan genes, annotated transfer RNA (tRNA) genes separately by using tRNAscan-SE [79] and Arwen [80], and checked the integrity of the assembly by aligning the reads to the completed mitochondrial genome. With the mitochondrial sequences removed, we generated 2 “sub-optimal” assemblies using Platanus v1.2.1 with *k-*mer size of 32 and 45 bp and default settings. Subsequently, we used these sub-optimal assemblies to construct artificial mate-pair libraries for 9 insert sizes (1,000, 2,000, 3,000, 4,000, 5,000, 7,500, 10,000, 15,000, 20,000) with MateMaker v1.0 (RRID:SCR_017199) [81]. We used the artificial mate-pair libraries to scaffold the optimal assembly (generated using Platanus *k*-mer = 45) with SSPACE Standard v3.0 [82]. This process produced a draft assembly with 417,008 scaffolds measuring a total of 209.3 Mb with an N50 of 16,443 bp (Table 1) (ENA Accession OFHS01000000). We recovered 91.94% (61.29% complete and 30.65% partial) of the core eukaryotic genes and 85.07% (70.86% complete and 14.21% partial) of the core metazoan genes with CEGMA and BUSCO, respectively.

### *Alatina alata* sequencing and assembly

Illumina library preparation and sequencing was conducted at the University of Kansas Genome Sequencing Core. Libraries were generated with the Illumina Nextera Library Preparation Kit (San Diego, CA, USA) and sequenced twice on the Illumina HiSeq 2500. The 2 different runs were performed on the same library: 1 with 100-bp paired-end, and 1 with 150-bp paired-end sequencing, resulting in 564 million reads totaling 148.6 Gb of paired-end sequence data. Pacific Biosciences (PacBio) library prep and sequencing were completed at the University of Washington Northwest Genomics Center. We constructed the libraries with unsheared DNA with end-cleanup only, with an average insert size of 6,000 bp. Sequencing was completed on the PacBio RS II platform, resulting in 486,000 long-reads totaling 990.2 Mb of data. We conducted hybrid assembly of Illumina short-reads and PacBio long-reads using MaSuRCA 3.2.2 [83] (which includes an error correction step for paired-end reads), which resulted in an assembly of 291,445 contigs and an N50 of 7,049 bp (NCBI Accession PUGI00000000). We did not perform adapter trimming prior to assembly because the MaSuRCA manual advises against preprocessing of reads, including adapter removal. Nevertheless, we identified considerable adapter contamination in our final assembly. Subsequently, we used a custom script (remove_adapters_and_200.pl [74]) to remove adapters and sequences shorter than 200 nucleotides. The total length of the assembly was 851.1 Mb. We recovered 29.84% (8.06% complete and 21.78% partial) of the core eukaryotic genes and 32.11% (18.30% and 13.81% partial) of the core metazoan genes with CEGMA and BUSCO, respectively. The low recovery rates for conserved genes in the *A. alata* genome are likely due to the considerably larger size of the genome, which tends to be coupled with long introns, and therefore higher rates of gene fragmentation in a draft assembly [84–86].

### Comparison of Assemblies

A comparison of the draft genomes assembled in this study reveals that the genome of *A. alata* is 4 times the size of that of *C. cruxmelitensis* and almost twice the size of the genome of *C. xamachana*. The apparent contiguity of the assemblies is reflected in this size difference, with the N50 of the *A. alata* genome (7,049 bp) being considerably smaller for both *C. cruxmelitensis* (16,443 bp) and *C. xamachana* (15,563 bp). The N50, the minimum length of at least half the contigs/scaffolds in an assembly, tends to scale with the level of completeness as measured by CEGMA and BUSCO. For example, CEGMA recovered 91.94% of 248 conserved eukaryotic genes (complete + partial) in *C. cruxmelitensis* and 82.66% in *C. xamachana*, and 29.84% in *A. alata* (Table 1).

### Gene Model Prediction

We predicted genes for all 3 genomes using Augustus v3.2.2 [87], with the *Homo sapiens* training set and hits generated with BLAT [88] alignments of published transcriptome data (*C. cruxmelitensis* ENA accession = HAHC01000000; *C. xamachana* ENA accession = PRJEB21012; *A. alata* accession = PRJNA312373) to the genome assemblies of the respective taxa [11]. The *H. sapiens* training set was used because Augustus gene predictions using the *Nematostella vectensis* v1.0 training set failed to detect intronic regions within predicted genes, thereby resulting in predicted proteins consisting of single exons. We generated 66,156 gene models for *A. alatina*, 26,258 for *C. cruxmelitensis*, and 31,459 for *C. xamachana*.

## Gene Orthology and Lineage-Specific Gene Ontology

We used OrthoFinder v1.1.4 [89] to construct orthologous groups between gene models of *A. alatina, C. cruxmelitensis, C. xamachana, N. vectensis, Hydra magnipapillata*, and *H. sapiens*. We also included translated transcriptome assemblies for *A. alatina, C. cruxmelitensis*, and *C. xamachana* in these ortholog analyses, as well as an additional transcriptome of the apo-symbiotic polyp stage of *C. xamachana*, which was assembled using Trinity v2.4.0 [90] with default settings (ENA Project Accession: PRJEB23739). All transcriptomes were translated using TransDecoder 3.0.0 [91] with minimum protein length (-m) set to 50 and all other settings as default. We annotated orthogroups by BLASTing a representative species against the Uniprot/Swiss-Prot database [92]. Orthogroups with annotations were further mapped to Gene Ontology (GO) terms, and analysed for putative enrichment related to biological function using ClusterProfiler [93], against a *C. xamachana* annotation database generated using AnnotationForge [94].

**Figure 2:**
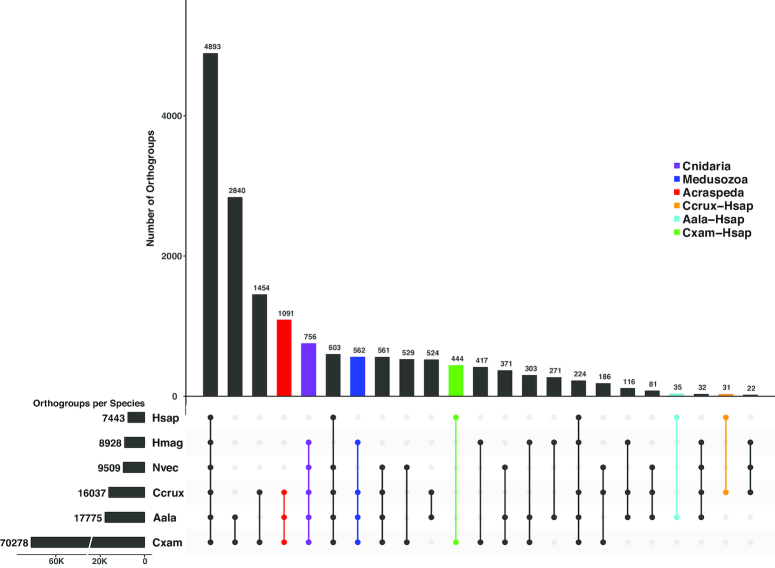
Gene content distribution in cnidarian lineages. Filled circles in the bottom panel indicate shared orthogroups in those lineages. Bar graphs indicate the number of orthogroups corresponding to each filled-circle pattern. Numbers next to each species abbreviation indicate the total number of orthogroups identified for that species. Hsap = *Homo sapiens*; Nvec = *Nematostella vectensis*; Hmag = *Hydra magnipapillata*; Ccrux = *Calvadosia cruxmelitensis*; Aala = *Alatina alata*; Cxam = *Cassiopea xamachana*.

Our OrthoFinder analysis generated a total of 80,482 orthogroups for the combined genomic datasets. Using a custom script, we identified 756 Cnidaria-specific orthogroups, another 562 medusozoan-specific orthogroups, and yet another 1,091 Acraspeda-specific orthogroups (Fig. 2); genes in each taxon-specific orthogroup were non-overlapping. Of these unique orthogroups, we were able to retrieve Swiss-Prot annotations for 57% of Cnidaria-specific orthogroups, 32% of Medusozoa-specific orthogroups, and 55% of Acraspeda-specific orthogroups (Tables S1−S3). Unannotated orthogroups may represent taxonomically restricted genes or genes no longer discernable as such due to possibly extensive genetic mutation experienced in evolutionary history. Using this framework, we identified enriched GO terms, uncovering 123 terms corresponding to biological processes that appear to be enriched within Cnidaria: 107 for Medusozoa, and 14 for Acraspeda (adjusted *P*-value < 0.01). We used ReViGO to remove redundant GO terms from these initial lists and grouped them further through *k*-means clustering by Euclidean distance. The optimal number of clusters was predicted using the R package NbClust v3.0.

**Figure 3:**
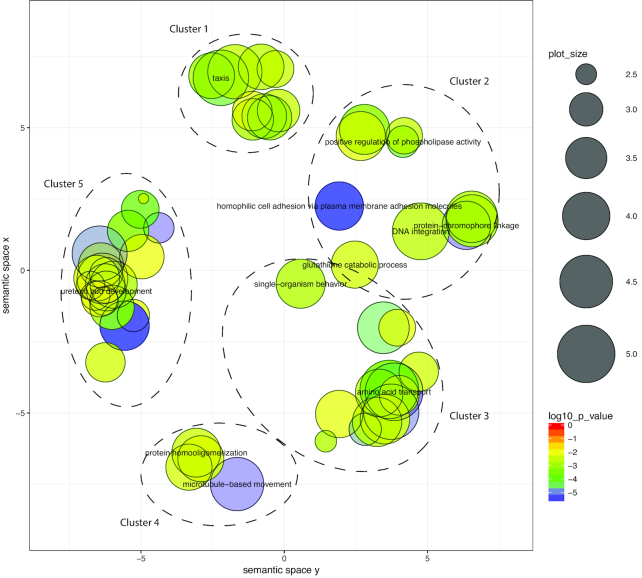
Gene ontology biological processes over-enriched within Cnidaria-specific orthogroups visualized using ReViGO. Over-representation analysis was performed with ClusterProfiler, with a *P*-adjusted cutoff of 0.01. Color indicates log_10_-transformed *P*-adjusted value. Terms are plotted within an *x-y* semantic space, in which similar terms are clustered within closer proximities. Color indicates *P*-value and circle size indicates frequency of GO term in the *Cassiopea* database.

**Figure 4:**
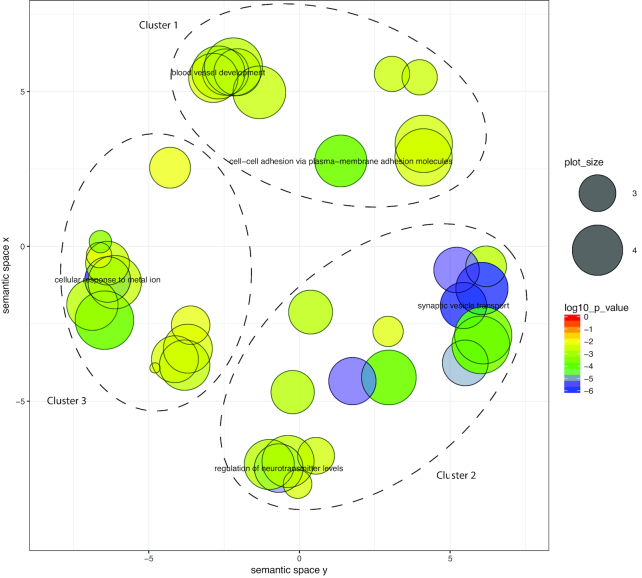
Gene ontology biological processes over-enriched within Medusozoa-specific orthogroups visualized using ReViGO. Over-representation analysis was performed with ClusterProfiler, with a *P*-adjusted cutoff of 0.01. Color indicates log_10_-transformed *P*-adjusted value. Terms are plotted within an *x-y* semantic space, in which similar terms are clustered within closer proximities. Color indicates *P*-value and circle size indicates frequency of GO term in the *Cassiopea* database.

**Figure 5:**
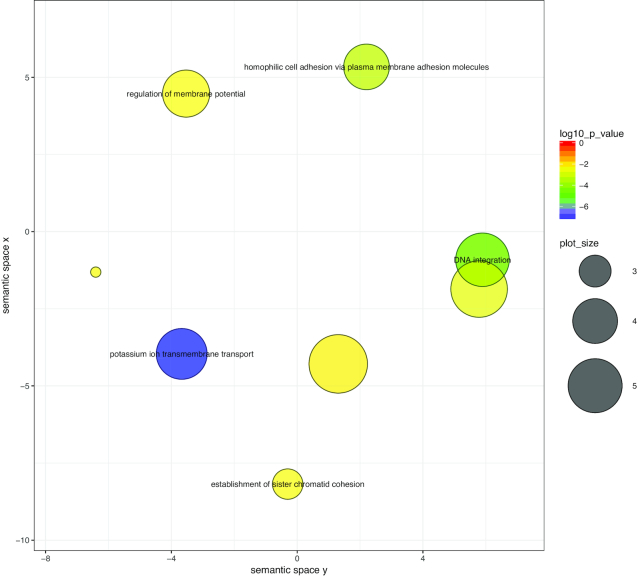
Gene ontology biological processes over-enriched within Acraspeda-specific orthogroups visualized using ReViGO. Over-representation analysis was performed with ClusterProfiler, with a *P*-adjusted cutoff of 0.01. Color indicates log_10_-transformed *P*-adjusted value. Terms are plotted within an *x-y* semantic space, in which similar terms are clustered within closer proximities. Color indicates *P*-value and circle size indicates frequency of GO term in the *Cassiopea* database.

Our ReViGO analysis reduced the 123 Cnidaria-specific GO terms for biological processes to 59 non-redundant ReViGO terms comprising 5 clusters (Fig. 3, Table S1, Fig. S1). Within the 5 clusters, many genes putatively encoding proteins for the cnidarian nerve net were represented (e.g., development and sensory perception), indicative of a system exhibiting a complex response to physical and chemical stimuli. Additionally, terms related to transport (ion, amines, carbon compounds) and the extracellular matrix were also represented. Genes associated with the extracellular matrix are possibly linked to the cnidarian novelty, the mesoglea (the proteinaceous layer between the endoderm and ectoderm in these diploblastic animals). The 107 Medusozoa-specific GO terms were reduced to 41 non-redundant ReViGO terms, which when grouped by *k*-means formed 3 clusters (Fig. 4, Table S2, Fig. S2). Similar to terms represented within Cnidaria, Medusozoa terms were also associated with response to stimuli and the nervous system. Unique terms seemingly important to medusozoan biology were those related to wound healing and tissue migration, as well as apoptotic signaling regulation. These terms are possibly associated with unique asexual reductive traits (e.g., budding, fission, strobilation) seen within the medusozoan lineage. Despite initially identifying 1,091 orthogroups unique to Acraspeda, only 14 GO terms were enriched (highly abundant); this number was further reduced to 8 non-redundant ReViGO terms (Fig. 5, Table S3). The apparently low enrichment may reflect an under-representation of Acraspeda genes within the reference database. Interestingly, half of the terms were associated with DNA recombination.

## Venom Analysis

**Figure 6:**
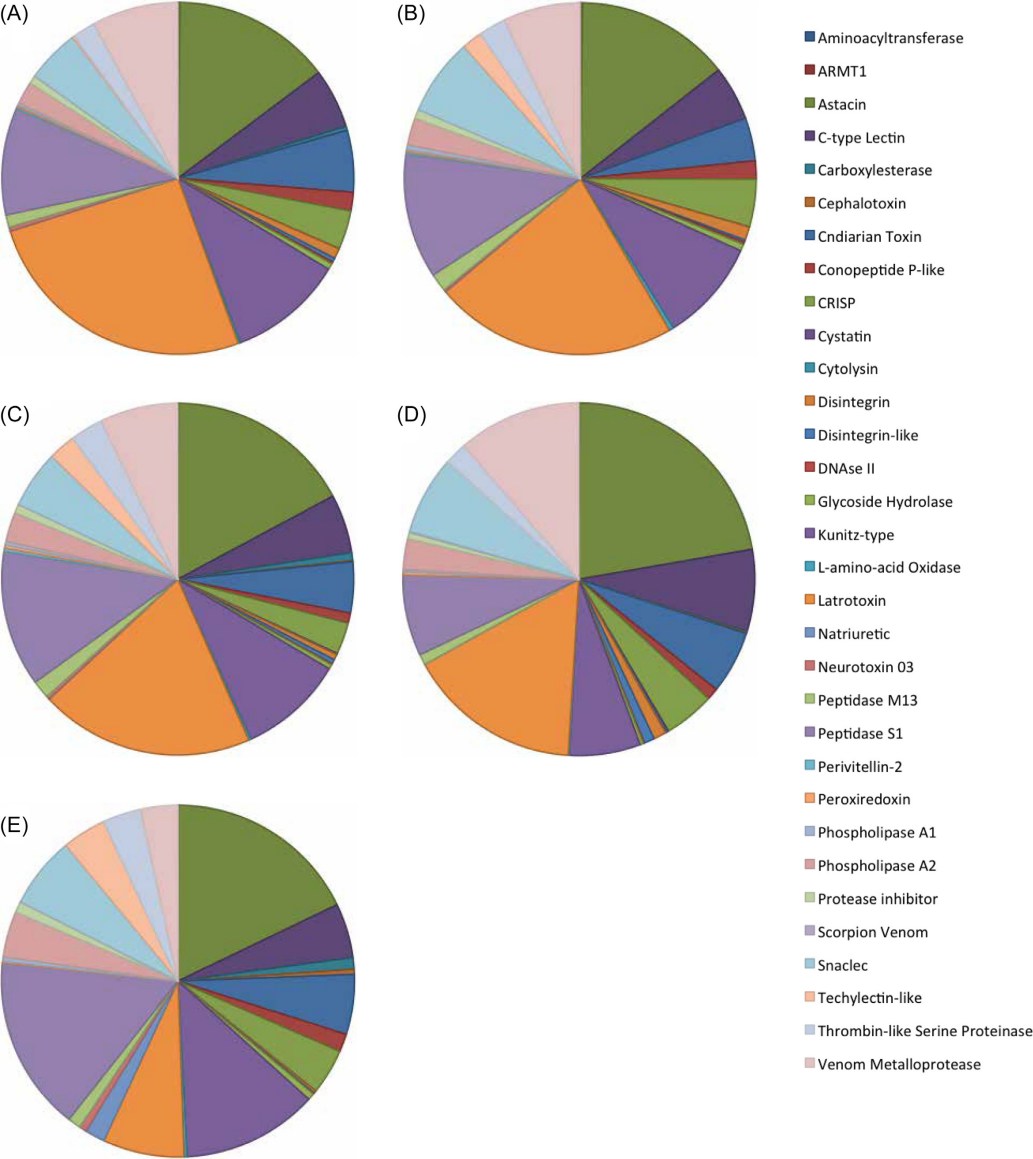
Venom-encoding gene repertoire of 5 cnidarian genomes. Venom-encoding genes were identified with the venomix database using OrthoFinder and BLAST. A, *Calvadosia cruxmelitensis*; B, *Alatina alata*; C, *Cassiopea xamachana*; D, *Hydra magnipapillata*; and E, *Nematostella vectensis*.

**Figure 7:**
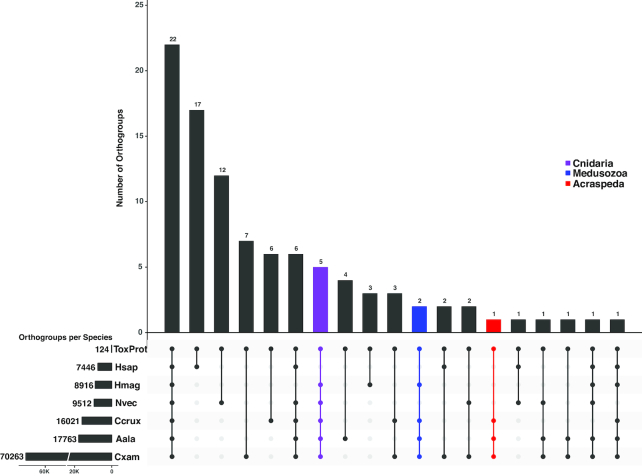
Distribution of venom-related genes in cnidarian lineages. Filled circles in the bottom panel indicate presence of shared venom-related genes in those lineages. Bar graphs indicate the number of venom-related orthogroups corresponding to each filled-circle pattern. Numbers next to each species abbreviation indicate the total number of venom-related orthogroups identified for that species. Hsap = *Homo sapiens*; Nvec = *Nematostella vectensis*; Hmag = *Hydra magnipapillata*; Ccrux = *Calvadosia cruxmelitensis*; Aala = *Alatina alata*; Cxam = *Cassiopea xamachana*.

We identified potential venom-encoding genes within the cnidarian transcriptomes using the venomix database (a publicly available curated set of 6,622 venom-related proteins) and associated pipeline [95]. Additional venom-encoding genes were identified by BLASTing the >6,000 venom-related protein sequences of the venomix database to the Augustus protein predictions (BLAST v2.2.31+ *e*-value = 10^−6^) [96]. By combining the results of both approaches, we identified 93 types of venom-encoding genes in *C. cruxmelitensis*, 93 in *A. alata*, 97 in *C. xamachana*, 96 in *H. magnipapillata*, and 91 in *N. vectensis*. In total, we identified 117 types of putative venom proteins, organized into 32 families, that were present in ≥1 of the 5 cnidarian taxa (Fig. 6, Table S4). To attempt to reconstruct evolutionary relationships among venom proteins in cnidarians, we added the venomix database to our initial set of input protein sequences and reran our OrthoFinder pipeline. Using this process, we identified 124 orthogroups encoding venom genes in the 5 cnidarian genomes and the human genome (Fig. 7). Of the 124 venom orthogroups, few were found to be specific to any 1 cnidarian lineage, with 5 orthogroups present across all cnidarians, 2 spanning medusozoans, and 1 shared between Acraspeda. Most of the proteins in the venomix database were identified first in bilaterian animals, and properly curated based on extensive supporting data, whereas putative toxins identified in non-model cnidarians often lack robust evidence to support annotations, precluding their entry into curated databases; hence the limited number of proteins returned in our homology search. However, we were successful in identifying 9 cnidarian-specific toxin proteins [4, 97–100]. Four of these proteins (potassium channel toxin BcsTx, peptide toxin Am-1, AvTx, MsepPTx) were found exclusively in *N. vectensis*, while CqTx was exclusive to the genome of *A. alatina*. Interestingly, CrTx, a toxin previously identified in Cubozoa, including *A. alatina* [99, 101], was also found in *C. xamachana* and *H. magnipapillata*. While the genome of *A. alatina* appears to possess 5 copies of the CrTx gene, we identified 3 putative copies in *C. xamachana* and 1 copy in *H. magnipapillata* genomes, respectively. However, CrTx was absent from both the *C. cruxmelitensis* genome and transcriptome, suggesting thath this gene may have been lost from the staurozoan lineage. Additionally, the pore-forming toxins sticholysin and hydralysin [97, 102] were found only in the *H. magnipapillata* genome in our analysis. Sticholysin was originally identified in the sea anemone *Stichodactyla helianthus*, but its absence from the *N. vectensis* genome may indicate that it is not an Anthozoa-specific protein, but rather variably distributed in Cnidaria.

## Hox-POU Synteny Analysis

**Figure 8:**
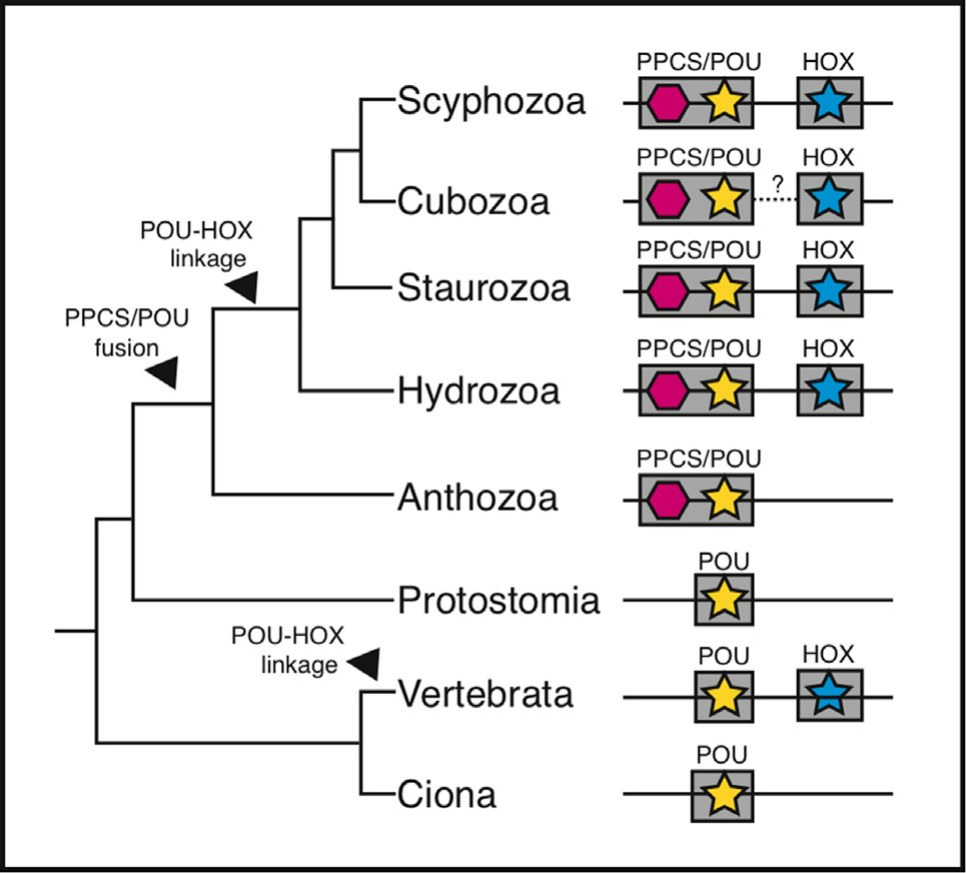
Evolution of PPCS/POU gene fusion and POU-Hox linkage. A fusion event involving a POU and PPCS domain occurred in the stem of Cnidaria. The syntenic linkage of POU and Hox genes occurred at least twice in animal evolution: once in the stem of Medusozoa and once in the vertebrate lineage.

In the hydrozoan *Eleutheria dichotoma*, a POU6-class homeobox gene is fused with a phosphopantothenoylcysteine-synthetase (PPCS), and this PPCS/POU6 fusion is linked to a Hox class homeobox gene, Cnox5 [48]. The PPCS/POU6 fusion is known only in Cnidaria, and its presence in the anthozoan *N. vectensis* suggests that it was likely present in the last common cnidarian ancestor. On the other hand, PPCS/POU6 fusion is not linked to a Hox-class gene in *N. vectensis* (cf. Putnam et al. 2007 assembly [55]), suggesting that POU-Hox linkage might be a more recent event. Of the Acraspeda genomes we find the PPCS/POU6 fusion gene linked to a Cnox5 ortholog in *C. cruxmelitensis* and *C. xamachana* (Fig. 8, Fig. S3). Considering that the last common medusozoan ancestor likely >500 million years ago [103], it is reasonable to conclude that a functional constraint has led to conserved synteny for PPCS/POU6 fusion. However, the linkage to Cnox5 was not recovered in the *A. alata* genome, preventing further speculation about whether POU-Hox linkage was present in the last common acraspedan ancestor.

Based on the well-established linkage of a POU-class homeobox gene to Hox clusters in vertebrates [48], it had been suggested that a POU-Hox linkage may have been present in the last common ancestor of cnidarians and bilaterians. To check this, we searched several additional anthozoan genomes: *Stylophora pistillata* [54] and *Acropora digitifera* [104], as well as several invertebrate bilaterian genomes: *Capitella teleta* (Polychaeta) [105], *Strigamia maritima* (Chilopoda) [106], *Octopus bimaculoides* (Cephalopoda) [107], *Mizuhopecten yessoensis* (Bivalvia) [108], and *Ciona intestinalis* (Ascidiacea) [109] that were not available at the time of the original study. We found no evidence for ancient POU-Hox synteny in these anthozoans nor in the invertebrate bilaterian genomes, suggesting that the POU-Hox linkage in medusozoans was achieved independently from the vertebrate POU-Hox linkage (Fig. 8, Fig. S3). These findings demonstrate how the 3 new medusozoan genomes allow us to address questions pertaining to molecular evolution, as well as the synergistic benefit of increased genomic-level taxon sampling when testing hypotheses about ancestral states.

## Conclusions

In this note we describe draft genomes for 3 species of the medusozoan sub-group Acraspeda (Cnidaria)—*C. cruxmelitensis* (Staurozoa), *A. alata* (Cubozoa), and *C. xamachana* (Scyphozoa)—and our corresponding bioinformatics workflows for their assemblies and partial annotations. The findings of our preliminary orthology analyses and annotation of Hox-linked and venom-related genes provide a glimpse into genetic components underlying the evolution of certain traits in these early metazoans. Coupled with appropriate bioinformatics tools and data management pipelines, researchers across a broad range of scientific fields can utilize these resources to investigate the genetic basis of defense, reproduction, and communication in this ancient and species-rich group that encompasses a diversity of life histories, some of which exhibit pelagic life stages. Furthermore, cnidarian genomes offer strategic opportunities to investigate possible genetic links to any number of ecological issues related to jellyfish that are frequently reported in the scientific literature, or in news media.

These medusozoan genomes will be useful resources in developing functional constructs (e.g., CRISPR/Cas9 guide RNAs) that can be used to understand the genomic basis for some of the captivating biological innovations of these animals, and eventually for the design of probes for target-capture DNA sequencing. Last, the availability of these genomic-level sequence data is an important step forward in the pursuit to elucidate evolutionary events that may have shaped Medusozoa, and in reconstructing the last common ancestor of Cnidaria and Bilateria. Therefore, we are confident that these new genomes will prove valuable for understanding the biology of these fascinating creatures, and for exploring key genomic events that were formative in the early evolution of animals.

## Availability of supporting data and materials

Accession numbers for raw sequencing reads and assemblies are available in Table 1. Custom scripts and parameters used for the analyses are available in a github repository [74]. Other data supporting this work are available in the *GigaScience* repository, GigaDB [110].

## Additional files

Supplementary Figure S1. ReViGO output for Cnidaria genes clustered through *k*-means clustering by Euclidean distance. Number of optimal clusters predicted prior to clustering using NbClust.

Supplementary Figure S2. ReViGO output for Medusozoa genes clustered through *k*-means clustering by Euclidean distance. Number of optimal clusters predicted prior to clustering using NbClust.

Supplementary Figure S3. Linkage of PPCS-POU genes with Hox genes in cnidarian genomes. Genomic scaffolds for 3 Medusozoa lineages (*Eleutheria dichotoma, Calvadosia cruxmelitensis*, and *Cassiopea xamachana*) show linkage of the PPCS-POU gene linked to a Hox gene (dark green). This linkage is not seen in Anthozoa (*Nematostella vectensis*). The light green region indicates the transcribed portion of the scaffold, and exons are represented within by curved rectangles (PPCS exons = purple, POU exons = yellow). Scaffold length shown to the right of each bar. Edic: *E. dichotoma*; Ccrux:*C.cruxmelitensis*; Cxam: C*. xamachana*; Nvec: *N. vectensis*.

Table S1. Orthogroups specific to Cnidaria identified using OrthoFinder and annotated by a representative gene from the *Hydra magnipapillata* genome. Protein annotations were retrieved from Swiss-Prot.

Table S2. Orthogroups specific to Medusozoa identified using OrthoFinder and annotated by a representative gene from the *Hydra magnipapillata* genome. Protein annotations were retrieved from Swiss-Prot.

Table S3. Orthogroups specific to Acraspeda identified using OrthoFinder and annotated by a representative gene from the *Hydra magnipapillata* genome. Protein annotations were retrieved from Swiss-Prot.

Table S4: Venom-encoding gene repertoire of 5 cnidarian genomes identified via the venomix database and pipeline. Venom genes are categories by families (column 1). Both genomic and transcriptomic data were used, with transcriptomic isoforms counted as a single venom-encoding gene.

giz069_GIGA-D-18-00115_Original_Submission

giz069_GIGA-D-18-00115_Revision_1

giz069_GIGA-D-18-00115_Revision_2

giz069_Response_to_Reviewer_Comments_Original_Submission

giz069_Response_to_Reviewer_Comments_Revision_1

giz069_Reviewer_1_Report_Original_Submission Ana Riesgo, PhD -- 6/4/2018 Reviewed

giz069_Reviewer_1_Report_Revision_1 Ana Riesgo, PhD -- 4/15/2019 Reviewed

giz069_Reviewer_2_Report_Original_Submission Paulyn Cartwright -- 6/4/2018 Reviewed

giz069_Reviewer_2_Report_Revision_1 Paulyn Cartwright -- 4/27/2019 Reviewed

giz069_Supplemental_Files

## Abbreviations

ABySS: Assembly By Short Sequencing; BLAST: Basic Local Alignment Search Tool; BLAT: BLASTlike Alignment Tool; bp: base pairs; BUSCO: Benchmarking Universal Single-Copy Orthologs; CEGMA: Core Eukaryotic Genes Mapping Approach; CRISPR: clustered regularly interspaced short palindromic repeats; CTAB: cetyl trimethylammonium bromide; ENA: European Nucleotide Archive; Gb: gigabase pairs; GO: Gene Ontology; MaSuRCA: Maryland Super-Read Celera Assembler; Mb: megabases pairs; NCBI: National Center for Biotechnology Information; PacBio: Pacific Biosciences; Platanus: PLATform for Assembling NUcleotide Sequences; PPCS: phosphopantothenoylcysteine synthetase; ReViGO: Reduce + Visualize Gene Ontology; SPAdes: St. Petersburg genome assembler; tRNA: transfer RNA.

## Competing interests

The authors declare that they have no competing interests.

## Funding

The sequencing of *Alatina alata* was funded by Iridian Genomes. A.O. acknowledges funding from an NSF Dimensions Grant (OCE 1442206); C.L.A. acknowledges funding from The University of Maryland Eugenie Clark Scholarship and a fellowship from Oak Ridge Institute for Science and Education (ORISE); startup funds from the University of Florida DSP Research Strategic Initiatives #00114464 and University of Florida Office of the Provost Programs to J.F.R.

## Author contributions

*Alatina alata* samples were collected by C.L.A., A.G.C., and S.P. *Alatina alata* assembly: R.B.D. and C.L.A. with B.B. and S.L.; *C. xamachana* assembly: A.O. and J.F.R.; *Calvadosia cruxmelitensis* assembly: M.C. and J.R. *Calvadosia cruxmelitensis* mitochondrial genome assembly: E.K. Orthology analyses: A.O. and J.R. The manuscript was drafted by A.O. with substantial contributions from C.L.A., A.G.C., and J.F.R. All coauthors read and provided feedback on the final manuscript. S.P., A.G.C., M.M., and J.R. oversaw the project from start to finish.
