# Supplementary material for: Box, stalked, and upside-down? Draft genomes from diverse jellyfish (Cnidaria, Acraspeda) lineages: Alatina alata (Cubozoa), Calvadosia cruxmelitensis (Staurozoa), and Cassiopea xamachana (Scyphozoa)
Source: Gigascience. 2019 Jul 1;8(7):giz069. doi: 10.1093/gigascience/giz069 (PMC6599738; doi:10.1093/gigascience/giz069)

GigaScience

Box, stalked and upside-down? Draft genomes from diverse jellyfish (Cnidaria, Acraspeda) lineages: Alatina alata (Cubozoa), Calvadosia cruxmelitensis (Staurozoa), and Cassiopea xamachana (Scyphozoa)

--Manuscript Draft--

**Manuscript Number:**	GIGA-D-18-00115
**Full Title:**	Box, stalked and upside-down? Draft genomes from diverse jellyfish (Cnidaria, Acraspeda) lineages: Alatina alata (Cubozoa), Calvadosia cruxmelitensis (Staurozoa), and Cassiopea xamachana (Scyphozoa)
**Article Type:**	Data Note
**Funding Information:**	University of Florida (US) DSP Research Strategic Initiatives and the Office of the Provost (Start Up) — Dr. Joseph F Ryan<br>University of Maryland, Howard J. Brinkley & Eugenie Clark Scholarships — Dr Cheryl Lewis Ames<br>Oakridge Institute for Science and Education's Scientific Visitor Program — Dr Cheryl Lewis Ames, Mr Sean La<br>Pennsylvania State University (Start Up) — Dr Mónica Medina<br>Iridian Genomes — Dr Stacy Pirro<br>National Science Foundation (US), Division of Ocean Sciences (OCE) (1442306) — Dr Mónica Medina
**Abstract:**	Anthozoa, Endocnidozoa, and Medusozoa comprise the three major clades of Cnidaria. Medusozoa is further divided into four clades, Hydrozoa, Staurozoa, Cubozoa, and Scyphozoa—the latter three lineages make up the clade Acraspeda. Acraspeda includes some of the most venomous organisms on the planet, numerous nuisance species, some of the most highly developed eyes in the animal kingdom, and it encompasses extraordinary diversity in terms of life history. Currently, no genomes are publicly available for any of these animals. Here we present three new draft genomes of Calvadosia cruxmelitensis (Staurozoa), Alatina alata (Cubozoa), and Cassiopea xamachana (Scyphozoa) for which we provide preliminary orthology analyses that includes an inventory of their known venom-related genes. To further demonstrate the utility of these datasets, we identify synteny between Pou and Hox genes that had previously been reported in a hydrozoan, suggesting that this linkage is highly conserved, dates back to at least the last common ancestor of Medusozoa, and is likely independent from the Hox-Pou linkages seen in vertebrates. These draft genomes provide a valuable resource for studying the evolutionary history and biology of these extraordinary animals, and for identifying genomic features underlying venom, vision, and life history traits.
**Corresponding Author:**	Joseph F. Ryan<br>Whitney Laboratory for Marine Bioscience<br>UNITED STATES
**Corresponding Author Secondary Information:**	
**Corresponding Author's Institution:**	Whitney Laboratory for Marine Bioscience
**Corresponding Author's Secondary Institution:**	
**First Author:**	Aki Hammond Ohdera
**First Author Secondary Information:**	
**Order of Authors:**	Aki Hammond Ohdera<br>Cheryl Lewis Ames

	Rebecca B Dikow
	Ehsan Kayal
	Marta Chiodin
	Ben Busby
	Sean La
	Stacy Pirro
	Allen G Collins
	Mónica Medina
	Joseph F Ryan
**Order of Authors Secondary Information:**	
**Additional Information:**	
**Question**	**Response**
Are you submitting this manuscript to a special series or article collection?	No
**Experimental design and statistics**<br><br>Full details of the experimental design and statistical methods used should be given in the Methods section, as detailed in our Minimum Standards Reporting Checklist. Information essential to interpreting the data presented should be made available in the figure legends.<br><br>Have you included all the information requested in your manuscript?	Yes
**Resources**<br><br>A description of all resources used, including antibodies, cell lines, animals and software tools, with enough information to allow them to be uniquely identified, should be included in the Methods section. Authors are strongly encouraged to cite Research Resource Identifiers (RRIDs) for antibodies, model organisms and tools, where possible.<br><br>Have you included the information requested as detailed in our Minimum Standards Reporting Checklist?	Yes
**Availability of data and materials**<br><br>All datasets and code on which the conclusions of the paper rely must be either included in your submission or	Yes

deposited in publicly available repositories (where available and ethically appropriate), referencing such data using a unique identifier in the references and in the "Availability of Data and Materials" section of your manuscript.<br><br>Have you have met the above requirement as detailed in our Minimum Standards Reporting Checklist?	

Manuscript
Click here to download Manuscript GigaNote.manuscrip.v10.5.docx

Box, stalked and upside-down? Draft genomes from diverse jellyfish (Cnidaria, Acraspeda) lineages: *Alatina alata* (Cubozoa), *Calvadosia cruxmelitensis* (Staurozoa), and *Cassiopea xamachana* (Scyphozoa)

Aki Ohdera[1], Cheryl L. Ames[2,3,4], Rebecca B. Dikow[2], Ehsan Kayal[2], Marta Chiodin[3], Ben Busby[4], Sean La[4,5], Stacy Pirro[6], Allen G. Collins[2], Mónica Medina[1*], Joseph F. Ryan[3*]

1. Department of Biology, Pennsylvania State University, University Park, PA, USA
2. Department of Invertebrate Zoology, National Museum of Natural History, Smithsonian Institution, Washington D.C., USA
3. Whitney Laboratory for Marine Bioscience, University of Florida, St. Augustine, FL, USA
4. National Center for Biotechnology Information, Bethesda, MD, USA
5. Department of Mathematics, Simon Fraser University, British Columbia, BC, Canada
6. Iridian Genomes, Inc. Bethesda, MD, USA
7. UPMC, CNRS, FR2424, ABiMS, Station Biologique Roscoff, France
8. National Systematics Laboratory of NOAA's Fisheries Service, Washington, DC, USA

* Corresponding authors

Author E-Mails:

AO – aohdera29@gmail.com
CLA – amesc@si.edu
RBD – dikowr@si.edu
EK – ehsan.kayal@gmail.com
MC – marta.chiodin@nyumc.org
BB – ben.busby@gmail.com
SL – laseanl@sfu.ca
SP – stacy734@yahoo.com
AGC – collinsa@si.edu
MM – mum55@psu.edu
JFR - joseph.ryan@whitney.ufl.edu

Abstract

Anthozoa, Endocnidozoa, and Medusozoa comprise the three major clades of Cnidaria. Medusozoa is further divided into four clades, Hydrozoa, Staurozoa, Cubozoa, and Scyphozoa—the latter three lineages make up the clade Acraspeda. Acraspeda includes some of the most venomous organisms on the planet, numerous nuisance species, some of the most highly developed eyes in the animal kingdom, and it encompasses extraordinary diversity in terms of life history. Currently, no genomes are publicly available for any of these animals. Here we present three new draft genomes of *Calvadosia cruxmelitensis* (Staurozoa), *Alatina alata* (Cubozoa), and *Cassiopea xamachana* (Scyphozoa) for which we provide preliminary orthology analyses that includes an inventory of their known venom-related genes. To further demonstrate the utility of these datasets, we identify synteny between Pou and Hox genes that had previously been reported in a hydrozoan, suggesting that this linkage is highly conserved, dates back to at least the last common ancestor of Medusozoa, and is likely independent from the Hox-Pou linkages seen in vertebrates. These draft genomes provide a valuable resource for studying the evolutionary history and biology of these extraordinary animals, and for identifying genomic features underlying venom, vision, and life history traits.

Keywords: Staurozoa, Scyphozoa, Cubozoa, Acraspeda, Cnidaria, Medusozoa

Context

Some of the most fascinating and outstanding mysteries in genome biology are centered around cnidarians. Active areas of research include the basis of venom evolution and diversification [1-3], mechanisms of independent evolution of image-forming vision (lens eyes) [4-6], and the emergence of a pelagic adult stage within a biphasic life cycle [7]. Cnidaria encompasses three major clades: Medusozoa, Anthozoa and Endocnidozoa. Medusozoans are characterized by the emergence of a medusa life history stage within some taxa of the clade, their high diversity in regards to life history and morphology, the presence of a linear mitochondrial genome (with a variable number of chromosomes), and by the presence of a hinged cap at the apex of the cnidocyst (cnidarian stinging organelles) [7-9].

There are approximately 3900 described species within Medusozoa, classified into four diverse lineages: Hydrozoa (hydroids, hydromedusae, siphonophores), Staurozoa (stalked jellyfish), Cubozoa (box jellyfish), and Scyphozoa (true jellyfish) (Figure 1A-C). There exists much debate regarding the phylogenetic relationships among these lineages [8, 10-13]. Recent phylogenomic analyses have placed Staurozoa as the sister to a clade that contains Cubozoa and Scyphozoa, uniting these lineages in a group called Acraspeda (Figure 1D) [14-16].

The mechanisms of medusa formation are variable amongst medusozoans: often involving two phenotypically distinct life stages - polyp and medusa - that are genotypically identical. Cubozoan polyps undergo partial or complete metamorphosis and develop into the adult medusoid form capable of sexual reproduction, although in some cases a polyp rudiment remains [17].

Scyphozoan polyps (scyphistomae) undergo a transition known as strobilation, in which the upper calyx proceeds through metamorphosis and transverse fission to produce a medusa [18]. Unlike other medusozoans, staurozoans lack a free-swimming medusa stage but exhibit medusa-associated characters that are present in other medusozoans. The basal portion of the adult forms a stalk, or peduncle, while coronal muscles and gastric filaments, among other features, characterizes the apical portion (calyx) of the adult [19, 20]. Hydrozoans exhibit the greatest variation in life history strategies and often lack a medusa form. Species that give rise to the medusoid form do so via lateral buds generated by asexual polyps, while others possess sexual polyps without a free-swimming stage [21, 22]. Elsewhere within Cnidaria, Anthozoa and the parasitic Endocnidozoa lack the medusa stage or medusoid characters entirely. Research on medusa development has shown similar gene expression patterns between hydrozoans and scyphozoans, with pre-existing developmental genes co-opted for patterning the medusa body plan [23, 24]. Interestingly, strobilation in scyphozoans was recently shown to be under the control of the retinoic acid pathway, [25, 26]. These same genes are involved in metamorphosis of insects and amphibians, hinting that the conservation of metamorphosis regulation might be conserved in metazoans.

The genomic resources necessary to understand medusozoan evolution have been lacking, with genomes currently available only for cnidarian models including the anthozoan *Nematostella vectensis* and the freshwater hydrozoan *Hydra* [27-29]. While the majority of Medusozoa species are represented by hydrozoans (>90%), both cubozoans and scyphozoans garner significant attention as a result of their impact on economy and tourism [30]. Largely due to

venom being employed as a mechanisms of defense and prey capture, the inherent risk of jellyfish sting has been exacerbated by uncertainty about how cnidarians will respond to modern-day anthropogenic disturbances along coastal environments [31, 32]. In addition, there has been increased interest in cnidarian venom for their pharmacological applications [33].

Here we present three new genomes, each belonging to a species representing one of the three major Acraspeda lineages: *Calvadosia cruxmelitensis* (formerly *Lucernariopsis cruxmelitensis*)(Staurozoa), *Alatina alata* (Cubozoa), and *Cassiopea xamachana* (Scyphozoa). Herein, we perform a range of preliminary gene inventory and synteny analyses to show the utility of these new data. The genomes and corresponding gene annotations from these three lineages will serve as useful resources aimed at sparking investigative research into the evolution and diversification of life history strategies across cnidarians, the evolution of venom within Cnidaria and phylogeographic patterns of venomous jellyfish, potential jellyfish-derived therapeutic drug development, as well as countless additional research programs.

Data Description

Cassiopea xamachana *Sample Collection and DNA extraction*

We propagated *C. xamachana* polyps from a single polyp via budding (Line T1-A). Polyps were maintained symbiont-free at 26 °C, and fed 3 times weekly with *Artemia* nauplii. To avoid the possibility of food-source contaminates interfering with downstream bioinformatic analysis, we starved the polyps for seven days in antibiotic-treated seawater prior to preservation in 95% ethanol, with any *Artemia* cysts still retained within the gut being manually

removed before preservation. We extracted genomic DNA using a CTAB (cetyl trimethylammonium bromide) phenol chloroform extraction. We performed an overnight digestion of polyps with proteinase K (20 mg/ml) in CTAB buffer before proceeding with the standard protocol. Extract DNA was stored at -20 °C until further processing.

Calvadosia cruxmelitensis *Sample Collectioin and DNA extraction*

We collected adult specimens of *C. cruxmelitensis* in January 2013 at Chimney Rock, off the coast of Penzance, Cornwall, England. Specimens were immediately preserved in ethanol and stored at -20 °C until further processing. We extracted genomic DNA using a phenol-choloroform protocol in an Autogen mass extractor, and stored the DNA at -20 °C.

Alatina alata *Sample Collection and DNA extraction*

We collected *A. alata* material during a spermcasting aggregation in Bonaire, The Netherlands (April, 2014, 22:00-01:00) according to the methods in Ames et al. (2016)[6]. We selected a single live spermcasting male medusa from the same cohort as the medusa used for RNA-Seq studies (Genbank Accession: GEUJ01000000) [6, 16]. We divided the medusa into four longitudinal sections, and one quarter was placed into a 15 ml tube with pure (99%) ethanol. We flash froze the tissue samples at -180 °C (using a dry shipper), and subsequently transported them to the Smithsonian NMNH where they were stored at -20 °C until genomic DNA extraction using a DNeasy Blood & Tissue Kit (Qiagen) following the manufacturer's protocol.

Cassiopea xamachana *Sequencing and Assembly*

Library construction and sequencing was performed at HudsonAlpha Institute for Biotechnology. Four 350 bp paired-end linear libraries with insert sizes of 500 bp were made with Illumina TruSeq DNA PCR-Free LT Prep Kits and sequenced on the Illumina Hiseq2000. Approximately 634 million reads totaling 117.6 Gb of high-quality paired-end sequence data were generated. We performed adaptor trimming and quality filtering using Trimmomatic v0.36 [34] with default settings, followed by error correction with Allpaths-LG version 52488 [35]. We removed mitochondrial reads using FastqSifter v1.1.1 (https://github.com/josephryan/FastqSifter; doi:10.5281/zenodo.1211357) with the *Cassiopea frondosa* mitochondrial genome as a reference (NCBI NC_016466.1). We performed *de novo* genome assemblies using ABySS 2.0.1 with default settings [36], SPAdes genome assembler v3.10.0 [37], and Platanus version 1.2.1 (with default parameters, k=89) [38] (Table 1). We used a custom Perl script, plat.pl, (https://github.com/josephryan/Ohdera_et_al_2018; doi:10.5281/zenodo.1211353) to invoke the Platanus commands for assembly, scaffolding, and gap closing. We generated a draft assembly with 142,229 scaffolds measuring a total of 400.8 Mb with an N50 of 15,024 Kb (Table 1) (ENA Accession OLMO01000000). We recovered 82.6% (54.03 % complete and 28.57 partial) of the core eukaryotic genes and 64.72% (56.96% complete and 7.76% partial) of the core metazoan genes with CEGMA ver. 2.5 [39] and BUSCO ver.2.01 [40], respectively, through the gVolante web server [41] (Table 1).

Calvadosia cruxmelitensis *Sequencing and Assembly*

Library construction and sequencing for *C. cruxmelitensis* were performed at the University of Florida Interdisciplinary Center for Biotechnology Research. Four 150 bp paired-end linear libraries and four 150 bp single-end linear libraries with insert size of 300 bp were generated and sequenced on the Illumina NextSeq 500. We performed adaptor trimming and quality filtering using Trimmomatic-0.32 [34] with default settings, followed by error correction using Allpaths-LG version [35]. We removed mitochondrial sequences to improve the final assembly with FastqSifter v1.1.1 using a *de novo* assembly of the *C. cruxmelitensis* mitochondrial genome. We assembled the *C. cruxmelitensis* mitochondrial genome by capturing contigs from an initial assembly using available staurozoan mitochondrial DNA sequences from NCBI as a reference, following the methods presented in Kayal et a. [42]. We used Geneious v9.0 to generate the final mitochondrial assembly. We checked completeness of the mitochondrial genome using NCBI BLAST against the nr database in addition to a manually generated set of medusozoan genes. We annotated tRNA genes separately by using tRNAscan-SE [43] and Arwen [44]. We checked the integrity of the assembly by aligning the reads to the completed mitochondrial genome. With the mitochondrial sequences removed, we generated two "sub-optimal" assemblies using Platanus v1.2.1 with kmer size of 32 bp and 45 bp and default settings. Subsequently, we used these "sub-optimal" assemblies to construct artificial mate-pair libraries for 9 insert sizes (1000, 2000, 3000, 4000, 5000, 7500, 10000, 15000, 20000) with MateMaker v.1.0.0 (https://github.com/josephryan/matemaker; doi:10.5281/zenodo.1211822). We used the artificial mate-pair libraries to scaffold the optimal assembly (generated using Platanus kmer=45) with SSPACE Standard v3.0 [45]. This

process produced a draft assembly with 417,008 scaffolds measuring a total of 260.1 Mb and an N50 of 11,886 Kb (Table 1) (ENA Accession OFHS01000000). We recovered 91.4 % (62.9% complete and 29.04 partial) of the core eukaryotic genes and 82.51% (64.03% complete and 18.48% partial) of the core metazoan genes with CEGMA and BUSCO, respectively.

Alatina alata *Sequencing and Assembly*

Illumina library prep and sequencing was conducted at the University of Kansas Genome Sequencing Core. Libraries were generated with the Illumina Nextera Library Preparation kit and sequenced twice on the Illumina HiSeq 2500. The two different runs were performed on the same library: one with 100 bp paired-end, and one with 150 bp paired-end sequencing, resulting in 564 million reads totaling 148.6 Gb of paired-end sequence data. PacBio library prep and sequencing were completed at the University of Washington Northwest Genomics Center. Libraries were constructed with unsheared DNA with end-cleanup only, and an average insert size of 6000 bp. Sequencing was completed on the PacBio RS II platform, resulting in 486,000 long-reads totaling 990.2 Mb of data. We performed error correction and subsequent hybrid assembly using Illumina short-reads and PacBio long-reads using MaSuRCA 3.2.2 [46], which resulted in an assembly of 338,010 contigs and an N50 of 7756 (NCBI Accession PUGI00000000). The total length of the assembly was 1.075 Gbp. We recovered 34.68 % (8.47% complete and 26.21% partial) of the core eukaryotic genes and 34.65% (21.78% and 12.87% partial) of the core metazoan genes with CEGMA and BUSCO, respectively.

Parameters for the assembly are available at https://github.com/josephryan/Ohdera_et_al_2018 (doi:10.5281/zenodo.1211353).

Gene Model Prediction

We predicted genes for all three genomes using Augustus v3.2.2 [47] . We used the *Nematostella vectensis* v1.0 training set (http://ryanlab.whitney.ufl.edu/downloads/nematostella_training_files_for_augustus.tar.gz) and hints generated with BLAT [48] alignments of transcriptome data (*C. cruxmelitensis* ENA accession= HAHC01000000; *C. xamachana* ENA accession= PRJEB21012; *A. alata* accession= PRJNA312373) to the genome assemblies. We generated 50,725 gene models for *A. alatina*, 27,276 for *C. cruxmelitensis*, and 24,718 for *C. xamachana*.

Orthologous Gene Analysis

We used OrthoFinder v1.1.4 [49] to construct orthologous groups between gene models of *A. alatina*, *C. cruxmelitensis*, *C. xamachana, N. vectensis, Hydra magnipapillata,* and *Homo sapiens.* We also included translated transcriptome assemblies for *A. alatina*, *C. cruxmelitensis*, and *C. xamachana* in these ortholog analyses, as well as an additional transcriptome of the apo-symbiotic polyp stage of *C. xamachana*, which was assembled using Trinity v2.4.0 [50] with default settings (ENA Project Accession: PRJEB23739). All transcriptomes were translated using TransDecoder 3.0.0 [51] with minimum protein length (-m) set to 50 and all other settings as default. Our OrthoFinder analysis generated a total of 78,227 orthogroups, with 1,187 species-specific

orthogroups (note: orthogroups with single sequences are not counted in these species-specific orthogroups). Using a custom script we identified 1,052 Acraspeda-specific orthogroups, 480 medusozoan-specific orthogroups, and 209 orthogroups present in *H. sapiens* and *N. vectensis* but not Medusozoa (Figure 2). All of these orthologous groups potentially play an important role in the evolution of Medusozoa. We hypothesize that the former two sets represent lineage-specific gains and the latter orthogroup set to represent potential gene loss events that occurred in the stem Medusozoa.

Venom Analysis

We identified potential venom-encoding genes from the transcriptomes using the venomix database (a curated set of 6,622 venom-related proteins) and associated pipeline (https://bitbucket.org/JasonMacrander/venomix). Transcripts that were identified as venom-encoding were searched against the protein predictions of the corresponding genomes with BLAST v2.2.31+ (e-value = 10^{-6}) [52] in order to confirm the presence of these genes within the genome assemblies. Transcripts identified with the venomix pipeline were treated as a single protein hit, and combined with the BLAST output (Table 2). We identified 119 families of putative venom proteins that were present in at least one of the five cnidarian taxa (Table 2). We identified 93 families of venom-encoding genes in both *C. cruxmelitensis* and *A.alata*, and 98 families in the *C. xamachana* genome. In addition to the venomix analysis, we analyzed the venom content of the genomes using OrthoFinder v1.1.4 by adding the venomix database to our initial set of input sequences. Using this process, we identified 123 orthogroups encoding venom genes that occurred in all five cnidarian genomes as well as the

human genome (Figure 3). Of the 123 venom orthogroups, few were found to be specific to any one cnidarian lineage, with only three orthogroups present across all cnidarians and one spanning just medusozoans. This is likely because most venom-related proteins in the venomix database were identified first in bilaterian animals, many of which are model organisms, whereas putative toxins recently identified in non-model cnidarians often lack robust annotations required for accession into this curated database. However, we were successful in identifying two taxon-specific toxin proteins, CrTX and CqTX, the amino acid sequences of which were first characterized in cubozoans, in the *A. alata, and C. xamachana* genomes [53, 54]. We identified seven and six putative proteins of CqTX and CrTX, respectively, from *A. alata*, while the *C. xamachana* genome contained only three CqTX genes and one CqTX gene. However, both toxins were absent from the *C. cruxmelitensis* genome and transcriptome, potentially indicating these genes may have been lost in the staurozoan lineage.

Hox-POU synteny analysis

In the hydrozoan *Eleutheria dichotoma*, a POU6 class homeobox gene is fused with a phosphopantothenoylcysteine-synthetase (PPCS); and this fusion gene is linked to a Hox class homeobox gene, Cnox5 [55]. The fusion of PPCS and POU6 is not seen outside of Cnidaria, but it is present in the anthozoan *Nematostella vectensis* suggesting it was present in the last common cnidarian ancestor. On the other hand the PPCS/POU6 fusion is not linked to a Hox class gene in *N. vectensis* (cf. Putnam et al. 2007 assembly [27]) suggesting this linkage might be a more recent event. We searched our three Acraspeda genomes for the presence of the PPCS/POU6 fusion gene and for synteny between this gene and

the ortholog to the *E. dichotoma* Cnox5. In the *C. cruxmelitensis* and *C. xamachana* genomes we find the PPCS/POU6 fusion gene linked to a Cnox5 ortholog (Figure 4). This result suggests that these two genes were linked in the last common medusozoan ancestor. Furthermore, considering that the last common medusozoan ancestor may have lived some 630-850 million years ago [56], it is reasonable to conclude that a functional constraint has led to this conservation in synteny.

Based on the well-established linkage of a POU class homeobox gene to Hox clusters in vertebrates [55], it had been suggested that a Pou-Hox linkage may have been present in the last common ancestor of cnidarians and bilaterians. To check this, we searched several additional anthozoan genomes: *Stylophora pistillata*, *Acropora digitifera*, *Orbicella faveolata*, as well as several invertebrate bilaterian genomes: *Capitella teleta* (Polychaeta), *Strigamia maritima* (Chilopoda), *Octopus bimaculoides* (Cephalopoda), *Mizuhopecten yessoensis* (Bivalvia), and *Ciona intestinalis* (Ascidiacea) that were not available at the time of the original study. We found no evidence for ancient Pou-Hox synteny in these anthozoans nor in the invertebrate bilaterian genomes, suggesting that the Hox-Pou linkage in medusozoans was achieved independently from the vertebrate Hox-Pou linkage. These findings demonstrate the utility of the three new medusozoan genomes in addressing questions pertaining to molecular evolution, as well as the synergistic effect that increased genomic-level taxon sampling can provide when testing hypotheses about ancestral state reconstruction.

Conclusions

In this note we provided draft genomes for three species of the medusozoan sub-group Acraspeda (Cnidaria)– *Calvadosia cruxmelitensis* (Staurozoa), *Alatina alata* (Cubozoa), and *Cassiopea xamachana* (Scyphozoa) – and our corresponding bioinformatics workflows for their assemblies and partial annotations. These assemblies represent the first published genomes for acraspedan jellyfish species and have been made available in the NCBI public database. The findings of our preliminary orthology analyses and annotation of Hox-linked and venom-related genes provide a glimpse into genetic components underlying the evolution of certain traits in these early metazoans. Coupled with appropriate bioinformatics tools and data management pipelines, researchers across a broad range of scientific fields can utilize these resources to investigate the genetic basis of defense, reproduction, and communication in this ancient and specious group that encompasses a diversity of life histories, some of which exhibit pelagic life stages. Furthermore, cnidarian genomes offer strategic opportunities to investigate possible genetic links to any number of ecological issues related to jellyfish that are frequently reported in the scientific literature, or in the news media.

These medusozoan genomes will be useful resources in developing functional constructs (e.g. CRISPR/Cas9 guide RNAs) that can be employed to understand the genomic basis for some of the captivating biological features of these animals. Lastly, the availability of these genomic-level sequence data is an important step forward in the pursuit to elucidate evolutionary events that may have shaped Medusozoa, and in reconstructing the last common ancestor of Cnidaria and Bilateria. Therefore, we are confident that these new genomes will prove essential for understanding key evolutionary genomic events that were formative in the early evolution of cnidarians and bilaterians.

Availability of supporting Data

Accession numbers for raw sequencing reads and assemblies are available in Table 1. Custom scripts and parameters used for the analyses are available at https://github.com/josephryan/Ohdera_et_al_2018 (doi:10.5281/zenodo.1211353).

Acknowledgement

This work was supported by start up funds to JFR from the University of Florida DSP Research Strategic Initiatives and the Office of the Provost, start up funds from Pennsylvania State University and funds from NSF Dimensions Grant #1443206 provided to MM. Funding was also provided to CLA and SL from the Oakridge Institute for Science and Education's Scientific Visitor Program for the *Alatina* assembly. CLA also acknowledges Dr. Rita Peachy, Director of CIEE Research Station, Bonaire, The Netherlands, and her staff for their assistance during collection. Additional funding for CLA was provided by the Howard J. Brinkley & Eugenia Clark Scholarships. We thank Jason Macrander for consultation regarding the Venomix database, Sheila Kitchen and Bishoy Hanna for advice on the *Cassiopea* genome assembly, as well as Alexandra Hernandez for providing comments on a previous version of this manuscript. We also thank David Fenwick for providing us with the *C. cruxmelitensis* panel.

1. Jouiaei M, Yanagihara AA, Madio B, Nevalainen TJ, Alewood PF and Fry BG. Ancient Venom Systems: A Review on Cnidaria Toxins. Toxins (Basel). 2015;7 6:2251-71. doi:10.3390/toxins7062251.
2. Jouiaei M, Sunagar K, Federman Gross A, Scheib H, Alewood PF, Moran Y, et al. Evolution of an Ancient Venom: Recognition of a Novel Family of Cnidarian Toxins and the Common Evolutionary Origin of Sodium and Potassium Neurotoxins in Sea Anemone. Molecular Biology and Evolution. 2015;32 6:1598-610. doi:10.1093/molbev/msv050.
3. Brinkman DL and Burnell JN. Biochemical and molecular characterisation of cubozoan protein toxins. Toxicon. 2009;54 8:1162-73. doi:10.1016/j.toxicon.2009.02.006.
4. Coates MM. Visual ecology and functional morphology of cubozoa (cnidaria). Integr Comp Biol. 2003;43 4:542-8. doi:10.1093/icb/43.4.542.
5. Liegertova M, Pergner J, Kozmikova I, Fabian P, Pombinho AR, Strnad H, et al. Cubozoan genome illuminates functional diversification of opsins and photoreceptor evolution. Sci Rep. 2015;5:11885. doi:10.1038/srep11885.
6. Lewis Ames C, Ryan JF, Bely AE, Cartwright P and Collins AG. A new transcriptome and transcriptome profiling of adult and larval tissue in the box jellyfish Alatina alata: an emerging model for studying venom, vision and sex. BMC Genomics. 2016;17:650. doi:10.1186/s12864-016-2944-3.
7. Collins AG. Phylogeny of Medusozoa and the evolution of cnidarian life cycles. Journal of Evolutionary Biology. 2002;15:418-32.
8. Bridge D, Cunningham CW, Schierwater B, DeSalle R and Buss LW. Class-level relationships in the phylum Cnidaria- Evidence from mitochondrial genome structure. Proceedings of the National Academy of Science. 1992;89:8750-3.
9. Reft AJ and Daly M. Morphology, Distribution, and Evolution of Apical Structure of Nematocysts in Hexacorallia. Journal of Morphology. 2012;273:121-36.
10. von Salvini-Plawen L. On the origin and evolution of the lower Metazoa. ZEITSCHRIFT FUR ZOOLOGISCHE SYSTEMATIK UND EVOLUTIONSFORSCHUNG. 1978;16:40-88.
11. Marques AC and Collins AG. Cladistic analysis of Medusozoa and cnidarian evolution. Invertebrate Biology. 2004;123 1:23-42.
12. Ortman BD, Bucklin A, Pagès F and Youngbluth M. DNA Barcoding the Medusozoa using mtCOI. Deep Sea Research Part II: Topical Studies in Oceanography. 2010;57 24-26:2148-56. doi:10.1016/j.dsr2.2010.09.017.
13. Miranda LS, Hirano YM, Mills CE, Falconer A, Fenwick D, Marques AC, et al. Systematics of stalked jellyfishes (Cnidaria: Staurozoa). PeerJ. 2016;4:e1951. doi:10.7717/peerj.1951.
14. Kayal E, Roure B, Philippe H, Collins AG and Lavrov DV. Cnidarian phylogenetic relationships as revealed by mitogenomics. BMC Evolutionary Biology. 2013;13 5:1.
15. Kayal E, Bentlage B, Pankey MS, Ohdera AH, Medina M, Plachetzki DC, et al. Comprehensive phylogenomic analyses resolve cnidarian relationships and the origins of key organismal traits. PeerJ Preprints. 2017:5:e3172v1. doi:10.7287/peerj.preprints.3172v1.

16. Zapata F, Goetz FE, Smith SA, Howison M, Siebert S, Church SH, et al. Phylogenomic Analyses Support Traditional Relationships within Cnidaria. PLoS One. 2015;10 10:e0139068. doi:10.1371/journal.pone.0139068.
17. Toshino S, Miyake H, Ohtsuka S, Adachi A, Kondo Y, Okada S, et al. Monodisc strobilation in Japanese giant box jellyfish *Morbakka virulenta* (Kishinouye, 1910): a strong implication of phylogenetic similarity between Cubozoa and Scyphozoa. Evol Dev. 2015;17 4:231-9. doi:10.1111/ede.12127.
18. Helm RR. Evolution and development of scyphozoan jellyfish. Biol Rev Camb Philos Soc. 2018; doi:10.1111/brv.12393.
19. Kikinger R and von Salvini-Plawen L. Development From Polyp to Stauromedusa in *Stylocoronella* (Cnidaria: Scyphozoa). Journal of the Marine Biological Association of the United Kingdom. 2009;75 04:899. doi:10.1017/s0025315400038236.
20. Miranda LS, Mills CE, Hirano YM, Collins AG and Marques AC. A review of the global diversity and natural history of stalked jellyfishes (Cnidaria, Staurozoa). Marine Biodiversity. 2017; doi:10.1007/s12526-017-0721-4.
21. Boero F, Bouillon J, Piraino S and Schmid V. Diversity of hydroidomedusan life cycles- ecological implications and evolutionary patterns. Proceedings of the 6th International Conference on Coelenterate Biology. 1997:56-62.
22. Bentlage B, Osborn KJ, Lindsay DJ, Hopcroft RR, Raskoff KA and Collins AG. Loss of metagenesis and evolution of a parasitic life style in a group of open ocean jellyfish. Molecular Phylogenetics and Evolution. 2018; doi:10.1016/j.ympev.2018.02.030.
23. Reber-Muller S, Streitwolf-Engel R, Yanze N, Schmid V, Stierwald M, Erb M, et al. BMP2/4 and BMP5-8 in jellyfish development and transdifferentiation. Int J Dev Biol. 2006;50 4:377-84. doi:10.1387/ijdb.052085sr.
24. Kraus JE, Fredman D, Wang W, Khalturin K and Technau U. Adoption of conserved developmental genes in development and origin of the medusa body plan. Evodevo. 2015;6:23. doi:10.1186/s13227-015-0017-3.
25. Fuchs B, Wang W, Graspeuntner S, Li Y, Insua S, Herbst EM, et al. Regulation of polyp-to-jellyfish transition in *Aurelia aurita*. Current biology : CB. 2014;24 3:263-73. doi:10.1016/j.cub.2013.12.003.
26. Brekhman V, Malik A, Haas B, Sher N and Lotan T. Transcriptome profiling of the dynamic life cycle of the scypohozoan jellyfish *Aurelia aurita*. BMC Genomics. 2015;16:74. doi:10.1186/s12864-015-1320-z.
27. Putnam NH, Srivastava M, Hellsten U, Dirks B, Chapman J, Salamov A, et al. Sea anemone genome reveals ancestral eumetazoan gene repertoire and genomic organization. Science. 2007;317 5834:86-94. doi:10.1126/science.1139158.
28. Chapman JA, Kirkness EF, Simakov O, Hampson SE, Mitros T, Weinmaier T, et al. The dynamic genome of *Hydra*. Nature. 2010;464 7288:592-6. doi:10.1038/nature08830.
29. Shinzato C, Shoguchi E, Kawashima T, Hamada M, Hisata K, Tanaka M, et al. Using the *Acropora digitifera* genome to understand coral responses to environmental change. Nature. 2011;476 7360:320-3. doi:10.1038/nature10249.

30. Nastav B, Malej M, Malej Jr A and Malej A. Is it possible to determine the economic impact of jellyfish outbreaks on fisheries? A Case Study – Slovenia. Mediterranean Marine Science. 2013;14 1:214. doi:10.12681/mms.382.
31. Purcell JE and Arai MN. Interactions of pelagic cnidarians and ctenophores with fish: a review. Hydrobiologia. 2001;451 1-3:27-44. doi:Doi 10.1023/A:1011883905394.
32. Purcell JE, Uye S and Lo W. Anthropogenic causes of jellyfish blooms and their direct consequences for humans: a review. Marine Ecology Progress Series. 2007;350:153-74. doi:10.3354/meps07093.
33. Jha RK and Zi-rong X. Biomedical Compounds from Marine organisms. Marine Drugs. 2004;2:123-46.
34. Bolger AM, Lohse M and Usadel B. Trimmomatic: a flexible trimmer for Illumina sequence data. Bioinformatics. 2014;30 15:2114-20. doi:10.1093/bioinformatics/btu170.
35. Gnerre S, MacCallum I, Przybylski D, Ribeiro FJ, Burton JN, Walker BJ, et al. High-quality draft assemblies of mammalian genomes from massively parallel sequence data. Proceedings of the National Academy of Sciences. 2011;108 4:1513-8. doi:10.1073/pnas.1017351108.
36. Simpson JT, Wong K, Jackman SD, Schein JE, Jones SJ and Birol I. ABySS: a parallel assembler for short read sequence data. Genome Res. 2009;19 6:1117-23. doi:10.1101/gr.089532.108.
37. Bankevich A, Nurk S, Antipov D, Gurevich AA, Dvorkin M, Kulikov AS, et al. SPAdes: a new genome assembly algorithm and its applications to single-cell sequencing. J Comput Biol. 2012;19 5:455-77. doi:10.1089/cmb.2012.0021.
38. Kajitani R, Toshimoto K, Noguchi H, Toyoda A, Ogura Y, Okuno M, et al. Efficient de novo assembly of highly heterozygous genomes from whole-genome shotgun short reads. Genome Res. 2014;24 8:1384-95. doi:10.1101/gr.170720.113.
39. Parra G, Bradnam K and Korf I. CEGMA: a pipeline to accurately annotate core genes in eukaryotic genomes. Bioinformatics. 2007;23 9:1061-7. doi:10.1093/bioinformatics/btm071.
40. Simao FA, Waterhouse RM, Ioannidis P, Kriventseva EV and Zdobnov EM. BUSCO: assessing genome assembly and annotation completeness with single-copy orthologs. Bioinformatics. 2015;31 19:3210-2. doi:10.1093/bioinformatics/btv351.
41. Nishimura O, Hara Y and Kuraku S. gVolante for standardizing completeness assessment of genome and transcriptome assemblies. Bioinformatics. 2017;33 22:3635-7. doi:10.1093/bioinformatics/btx445.
42. Kayal E, Bentlage B, Cartwright P, Yanagihara AA, Lindsay DJ, Hopcroft RR, et al. Phylogenetic analysis of higher-level relationships within Hydroidolina (Cnidaria: Hydrozoa) using mitochondrial genome data and insight into their mitochondrial transcription. PeerJ. 2015;3:e1403. doi:10.7717/peerj.1403.
43. Lowe TM and Eddy SR. tRNAscan-SE: A program for improved detection of transfer RNA genes in genomic sequence. Nucleic Acids Research. 1997;25 5:955-64. doi:DOI 10.1093/nar/25.5.955.

44. Laslett D and Canback B. ARWEN: a program to detect tRNA genes in metazoan mitochondrial nucleotide sequences. Bioinformatics. 2008;24 2:172-5. doi:10.1093/bioinformatics/btm573.
45. Boetzer M, Henkel CV, Jansen HJ, Butler D and Pirovano W. Scaffolding pre-assembled contigs using SSPACE. Bioinformatics. 2011;27 4:578-9. doi:10.1093/bioinformatics/btq683.
46. Zimin AV, Marcais G, Puiu D, Roberts M, Salzberg SL and Yorke JA. The MaSuRCA genome assembler. Bioinformatics. 2013;29 21:2669-77. doi:10.1093/bioinformatics/btt476.
47. Stanke M and Waack S. Gene prediction with a hidden Markov model and a new intron submodel. Bioinformatics. 2003;19 Suppl 2:ii215-ii25. doi:10.1093/bioinformatics/btg1080.
48. Kent WJ. BLAT -- The BLASTlike Alignment Tool. Genome Research. 2002;12:656-64.
49. Emms DM and Kelly S. OrthoFinder: solving fundamental biases in whole genome comparisons dramatically improves orthogroup inference accuracy. Genome Biol. 2015;16:157. doi:10.1186/s13059-015-0721-2.
50. Grabherr MG, Haas BJ, Yassour M, Levin JZ, Thompson DA, Amit I, et al. Full-length transcriptome assembly from RNA-Seq data without a reference genome. Nat Biotechnol. 2011;29 7:644-52. doi:10.1038/nbt.1883.
51. Haas BJ, Papanicolaou A, Yassour M, Grabherr M, Blood PD, Bowden J, et al. De novo transcript sequence reconstruction from RNA-Seq: reference generation and analysis with Trinity. Nature protocols. 2013;8 8:10.1038/nprot.2013.084. doi:10.1038/nprot.2013.084.
52. Camacho C, Coulouris G, Avagyan V, Ma N, Papadopoulos J, Bealer K, et al. BLAST+: architecture and applications. BMC Bioinformatics. 2009;10:421. doi:10.1186/1471-2105-10-421.
53. Nagai H, Takuwa K, Nakao M, Sakamoto B, Crow GL and Nakajima T. Isolation and characterization of a novel protein toxin from the Hawaiian box jellyfish (sea wasp) Carybdea alata. Biochem Biophys Res Commun. 2000;275 2:589-94. doi:10.1006/bbrc.2000.3352.
54. Nagai H, Takuwa-Kuroda K, Nakao M, Oshiro N, Iwanaga S and Nakajima T. A novel protein toxin from the deadly box jellyfish (Sea Wasp, Habu-kurage) Chiropsalmus quadrigatus. Biosci Biotechnol Biochem. 2002;66 1:97-102. doi:10.1271/bbb.66.97.
55. Kamm K and Schierwater B. Ancient linkage of a POU class 6 and an anterior Hox-like gene in cnidaria: implications for the evolution of homeobox genes. J Exp Zool B Mol Dev Evol. 2007;308 6:777-84. doi:10.1002/jez.b.21196.
56. Rogers AD. Cnidarians (Cnidaria). In: Hedges SB and Kumar S, editors. The Timetree of Life. Oxford University Press, USA; 2009. p. 233-8.

Figure 1. Photos of Acraspeda and Phylogeny of Cnidaria. A) *Calvadosia cruxmelitensis* (Staurozoa), B) *Alatina alata* (Cubozoa), and C) *Cassiopea xamachana* (Sycphozoa). D) Relationship of major cnidarian lineages after Kayal et al. (2018).

Figure 2. Gene Content Distribution in Cnidarian Lineages. Filled circles in the bottom panel indicate shared orthogroups in these lineages. Bar graphs indicate the number of orthogroups corresponding to each filled-circle pattern. Numbers next to each species abbreviation indicate the total number of orthogroups identified for that species. Hsap = *Homo sapiens*; Nvec = *Nematostella vectensis*; Hmag = *Hydra magnipapillata*; Ccrux = *Calvadosia cruxmelitensis*; Aala = *Alatina alata*; Cxam = *Cassiopea xamachana*.

Figure 3. Distribution of venom-related genes in cnidarian lineages. Filled circles in the bottom panel indicate presence of venom-related gene in each lineage. Bar graphs indicate the number of venom-related orthogroups corresponding to each filled-circle pattern. Numbers next to each species abbreviation indicate the total number of venom-related orthogroups identified for that species. Hsap = *Homo sapiens*; Nvec = *Nematostella vectensis*; Hmag = *Hydra magnipapillata*; Ccrux = *Calvadosia cruxmelitensis*; Aala = *Alatina alata*; Cxam = *Cassiopea xamachana*.

Figure 4. Linkage of PPCS-Pou Genes with Hox Genes in Medusozoa Genomes. Genomic scaffolds from three Medusozoa lineages (*C. xamachana, C. cruxmelitnesis*, and *E. dichotoma*) show linkage of the PPCS-POU gene linked to a Hox gene (dark green). This linkage is not seen in Anthozoa (*N. vectensis*). Scaffold length is shown to the right of the bar. The light green region indicates the transcribed portion of the scaffold, and exons are represented within by curved rectangles (PPCS exons = purple, POU exons = yellow). Edic = *Eleutheria dichotoma*; Ccrux = *Calvadosia cruxmelitensis*; Cxam = *Cassiopea xamachana;* Nvec = *Nematostella vectensis*.

	Alatina alata	*Calvadosia cruxmelitensis*	*Cassiopea xamachana*
NCBI Taxa ID	1193083	1843192	12993
# of Sequences	337,986	50,999	93,483
Total Length (bp)	1,075,356,741	209,392,379	393,520,168
N50	7,756	16,443	15,563
CEGMA (Complete)	8.47	62.9	54.03
CEGMA (Complete + Partial)	34.68	91.94	82.66
BUSCO (Complete)	21.78	64.03	56.96
BUSCO (Complete + Partial)	34.65	82.51	64.72
GC Content (%)	38.02	39.67	37.45
Assembly Accession	PUGI00000000	OFHS01000000	OLMO01000000
Raw Read Accession (NCBI)	PRJEB421156	PRJEB23739	PRJEB23739
Specimen Voucher ID	USNM 1248604	USNM 1286381	UF Cnidaria 12979

Table 1. Statistics of the genomic assemblies of the three Acraspeda genomes.

PPCS exons = purple, POU exons = yellow, Hox exons = green.

Family	ID	Cc	Aa	Cx	Hm	Nv
Acetylcholinesterase	Acetylcholinesterase-1_1	0	6	10	1	1
Acidic_phospholipase	Acidic_phospholipase_A2_1_322	1	1	1	2	4
	Acidic_phospholipase_A2_2_1	0	0	0	0	3
	Acidic_phospholipase_A2_daboiatoxin_B_chain_6	0	0	0	0	6
Alpha-amylase	Alpha-amylase_1	0	0	1	0	0
Alpha-latroinsectotoxin	Alpha-latroinsectotoxin-Lh1a_1	0	2	6	3	0
	Alpha-latroinsectotoxin-Lt1a_2	30	61	45	3	3
Astacin-like_ metalloprotease_toxin	Astacin-like_metalloprotease_toxin_1_1	24	7	8	5	10
	Astacin-like_metalloprotease_toxin_3_2	10	7	8	5	10
	Astacin-like_metalloprotease_toxin_4_1	11	7	8	5	10
	Astacin-like_metalloprotease_toxin_5_1	11	7	8	5	10
Basic_phospholipase	Basic_phospholipase_A2_2_60	1	0	0	2	2
	Basic_phospholipase_A2_acanthin-1_3	1	1	1	2	4
	Basic_phospholipase_A2_notexin_18	2	1	1	2	8
C-type_lectin	C-type_lectin_6	12	6	13	11	27
	C-type_lectin_galatrox_1	1	0	2	7	0
	C-type_lectin_mannose-binding_isoform_30	9	8	17	11	27
Conodipine-M_alpha_chain_1	Conodipine-M_alpha_chain_1	1	1	1	2	2
	Cystatin-1_1	0	0	1	2	0
Cysteine-rich	Cysteine-rich_venom_protein_1_2	0	0	0	0	2
	Cysteine-rich_venom_protein_bucarin_1	1	1	1	2	0
	Cysteine-rich_venom_protein_Mr30_2	2	2	3	2	4
Cytolysin_RTX	Cytolysin_RTX-S-2_2	0	0	0	4	0
Delta-latroinsectotoxin	Delta-latroinsectotoxin-Lt1a_1	19	53	45	3	3
Disintegrin	Disintegrin_acostatin-alpha_26	3	1	1	0	0
	Disintegrin_bitistatin_6	4	1	2	4	5
	Disintegrin_EMF10B_17	3	0	0	0	0
	Disintegrin_lebein-1-beta_8	2	0	0	0	0
	Disintegrin_saxatilin_24	3	1	1	4	5
Disintegrin-like	Disintegrin-like_leberagin-C_8	5	1	2	4	5
Factor_V	Factor_V_activator_RVV-V_gamma_142	3	3	4	5	11
Galactose-specific_lectin	Galactose-specific_lectin_nattectin_1	14	8	21	11	20
Hyaluronidase	Hyaluronidase_1	1	1	1	2	1
	Hyaluronidase_CdtHya1_1	0	0	0	0	1
	Hyaluronidase_conohyal-ad1_1	1	1	1	2	1
	Hyaluronidase_conohyal-Cn1_1	1	1	2	2	1
Hydralysin	Hydralysin-1_6	0	0	0	2	0
KappaPI-theraphotoxin	KappaPI-theraphotoxin-Hs1a_80	6	2	2	6	6
Kunitz-type	Kunitz-type_conkunitzin-S1_2	3	1	1	4	4
	Kunitz-type_proteinase_inhibitor_AEPI-IV_2	1	0	0	2	0
	Kunitz-type_serine_protease_inhibitor_2_4	3	4	1	4	5
	Kunitz-type_serine_protease_inhibitor_As-fr-19_1	1	2	1	6	4
	Kunitz-type_serine_protease_inhibitor_bicolin_1	0	0	0	2	1
	Kunitz-type_serine_protease_inhibitor_bitisilin-3_1	5	5	1	6	8
	Kunitz-type_serine_protease_inhibitor_BmKTT-2_1	6	4	1	6	7
	Kunitz-type_serine_protease_inhibitor_BmKTT-3_1	5	3	1	6	6
	Kunitz-type_serine_protease_inhibitor_conotoxin_Cal9	4	4	1	5	7
	Kunitz-type_serine_protease_inhibitor_Hg1_1	6	4	1	7	7
	Kunitz-type_serine_protease_inhibitor_ homolog_alpha-dendrotoxin_3	2	1	1	3	6
	Kunitz-type_serine_protease_inhibitor_ homolog_beta-bungarotoxin_B5-B_chain_1	1	1	1	6	3
	Kunitz-type_serine_protease_inhibitor_ homolog_delta-dendrotoxin_2	5	1	2	2	6
	Kunitz-type_serine_protease_inhibitor_ homolog_dendrotoxin_K_154	7	4	2	6	8
	Kunitz-type_serine_protease_inhibitor_ Kunitz-1_1	7	4	3	6	8
	Kunitz-type_serine_protease_inhibitor_ PIVL_13	5	2	1	5	8
	Kunitz-type_serine_protease_inhibitor_ U1-aranetoxin-Av1a_1	7	4	2	7	7
L-amino-acid_oxidase	L-amino-acid_oxidase_31	1	1	1	3	7
Nematocyte	Nematocyte_expressed_protein_6_1	22	7	7	5	10
Neprilysin	Neprilysin-1_1	4	2	5	2	2
Neutral_phospholipase	Neutral_phospholipase_A2_3_21	1	1	1	2	4

Peptide Toxin	Peptide_toxins_Am-1_1	0	0	0	0	2
Perivitellin	Perivitellin-2_31_kDa_subunit_1	0	0	1	0	0
	Perivitellin-2_67_kDa_subunit_1	2	1	2	0	0
Peroxiredoxin	Peroxiredoxin-4_1	1	1	1	2	2
Phospholipase	Phospholipase_A1_19	1	1	1	2	2
	Phospholipase_A2_1	3	1	2	3	8
	Phospholipase_A2_2	0	0	1	0	0
	Phospholipase_A2_homolog_crotoxin_acid_subunit_CA_21	1	1	1	2	4
	Phospholipase_A2_imperatoxin-1_4	1	1	1	2	0
	Phospholipase_A2_large_subunit_1	1	1	1	2	0
	Phospholipase_A2_phaiodactylipin_6	1	1	0	2	0
	Phospholipase_A2_Scol@Pla_1	1	1	1	2	4
Plancitoxin	Plancitoxin-1_1	1	1	1	2	0
Potassium Channel Toxin	Potassium_channel_toxin_BcsTx3_1	0	0	0	0	2
Protease inhibitor	Protease_inhibitor_1_6	7	4	2	9	8
Putative protein-glutamate O-methyltransferase	Putative_antimicrobial_peptide_7848_1	0	0	0	0	1
SE-cephalotoxin	Putative_protein-glutamate_O-methyltransferase_1	1	1	1	2	2
Serine_proteinase-like	SE-cephalotoxin_1	0	0	1	1	0
Snaclec (C-type lectin-like proteins)(CLPs)	Serine_proteinase-like_BMK-CBP_1	0	1	1	3	0
	Snaclec_aspercetin_subunit_alpha_2	3	1	1	0	6
	Snaclec_botrocetin_subunit_beta_1	11	3	6	21	27
	Snaclec_coagulation_factor_IX@factor_X-binding_protein_subunit_A_1	9	3	10	5	15
	Snaclec_coagulation_factor_X-activating_enzyme_light_chain_2_135	12	3	10	11	24
	Snaclec_echicetin_subunit_alpha_1	9	5	4	8	17
	Snaclec_echicetin_subunit_beta_6	8	3	9	5	6
	Snaclec_lebecin_subunit_alpha_9	8	3	8	3	14
	Snaclec_rhodocetin_subunit_alpha_2	3	5	8	1	2
	Snaclec_rhodocetin_subunit_delta_1	10	5	11	22	27
	Snaclec_VP12_subunit_A_1	3	2	4	0	15
Snake_venom_ metalloproteinase	Snake_venom_metalloprotease_inhibitor_02A10_1	0	0	0	0	2
	Snake_venom_metalloproteinase_Ac1_1	3	3	1	4	6
	Snake_venom_metalloproteinase_acutolysin-C_1	3	3	1	4	6
	Snake_venom_metalloproteinase_fibrolase_19	3	3	1	4	7
	Sticholysin-2_8	0	0	0	4	0
Techylectin-like	Techylectin-like_protein_1	24	7	28	0	3
Thrombin-like_enzyme	Thrombin-like_enzyme_ancrod_11	4	3	5	5	53
	Thrombin-like_enzyme_TLBm_1	3	3	2	5	28
	Toxin_AvTX-60A_3	0	0	0	0	1
	Toxin_CqTX-A_3	0	6	1	0	0
	Toxin_CrTX-A_1	0	7	3	2	2
	Toxin_MsePTx1_1	0	0	0	0	0
	Turripeptide_Ici9	2	6	5	4	32
Turripeptide	Turripeptide_Pal9	1	5	2	4	28
	U24-ctenitoxin-Pn1a_1	3	1	1	0	1
Venom_allergen	Venom_allergen_3_30	5	4	2	3	23
	Venom_allergen_5	3	8	7	3	37
	Venom_allergen_5_1	5	5	6	3	36
	Venom_allergen_5_15	6	5	4	3	34
Venom peptide isomerase heavy chain	Venom_peptide_isomerase_heavy_chain_2	6	4	7	5	58
	Venom_peptide_SjAPI-2_1	0	0	0	0	1
	Venom_protein_59	0	0	0	2	4
Venom_prothrombin	Venom_prothrombin_activator_pseutarin-C_catalytic_subunit_12	5	5	9	5	58
Zinc-metalloproteinase	Zinc_metalloproteinase_recombinant_fibrinogenase_II_1	4	3	1	4	105
	Zinc_metalloproteinase-disintegrin-like_alternagin_2	3	1	2	4	88
	Zinc_metalloproteinase-disintegrin-like_ammodytagin_1	3	1	1	4	56
	Zinc_metalloproteinase-disintegrin-like_mikarin_1	3	1	1	4	56
	Zinc_metalloproteinase-disintegrin-like_VAP2B_272	8	4	4	4	145
	Zinc_metalloproteinase@disintegrin_107	6	4	1	4	139
	Zinc_metalloproteinase@disintegrin_4	4	1	1	4	76
	Zinc_metalloproteinase@disintegrin_5	4	1	1	4	83

Table 2. Venom-encoding genes identified from five cnidarian genomes using the venomix database.

Figure 1

Click here to download Figure Figure1.pdf

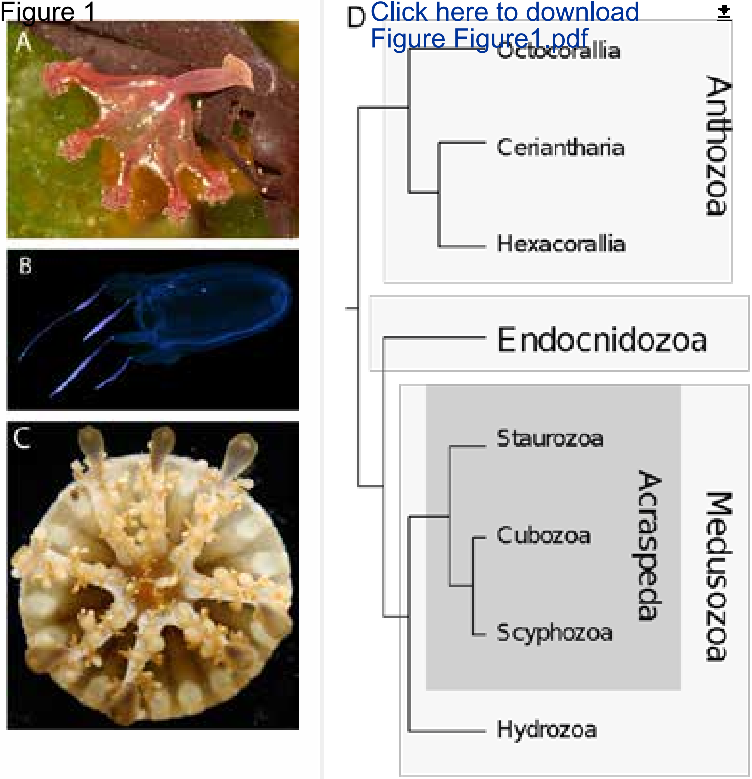

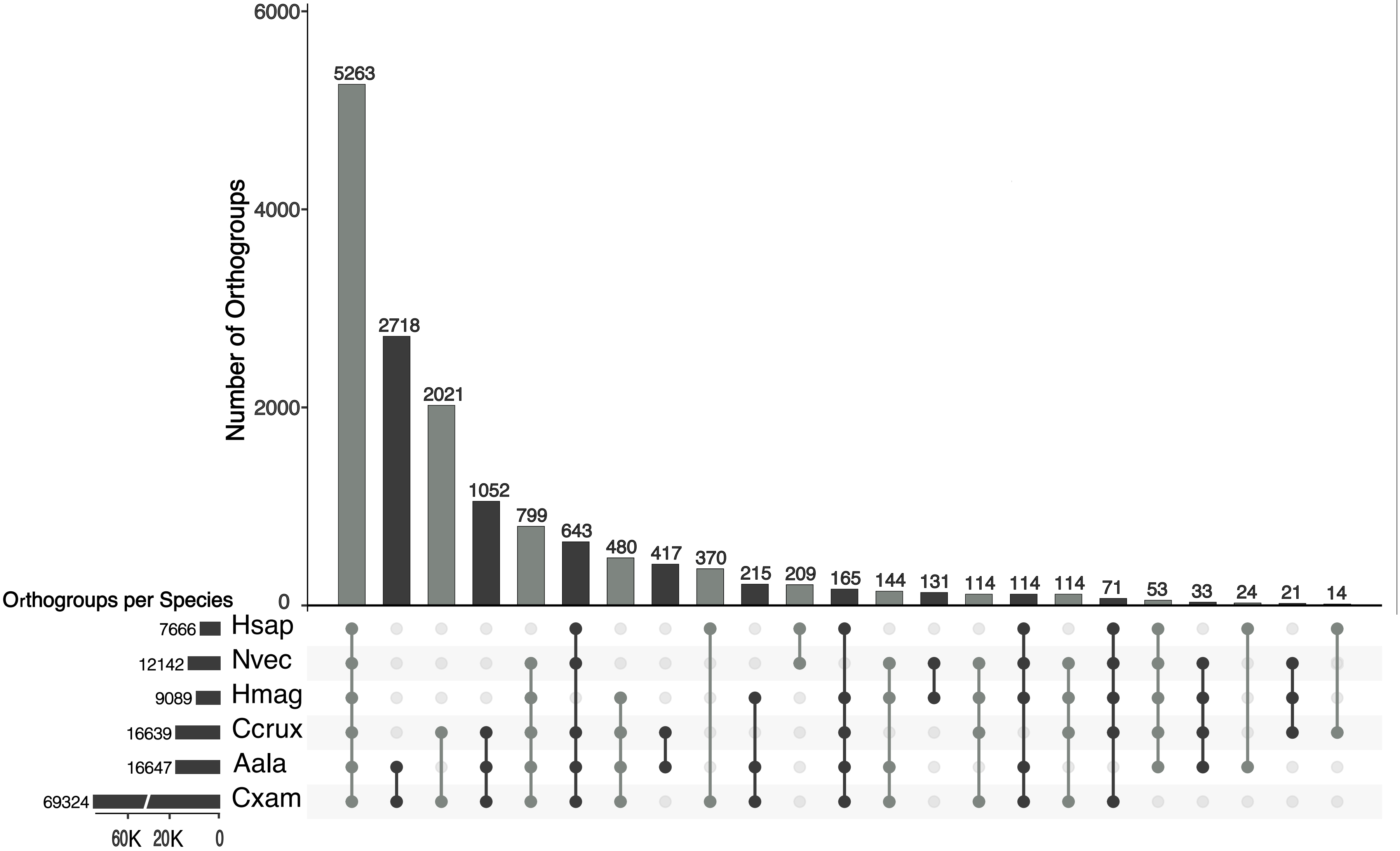

Number of Orthogroups
6000
4000
2000
0
5263
2718
2021
1052
799
643
480
417
370
215
209
165
144
131
114
114
114
71
53
33
24
21
14
Orthogroups per Species
7666
12142
9089
16639
16647
69324
Hsap
Nvec
Hmag
Ccrux
Aala
Cxam
60K
20K
0

Click here to download Figure Fig

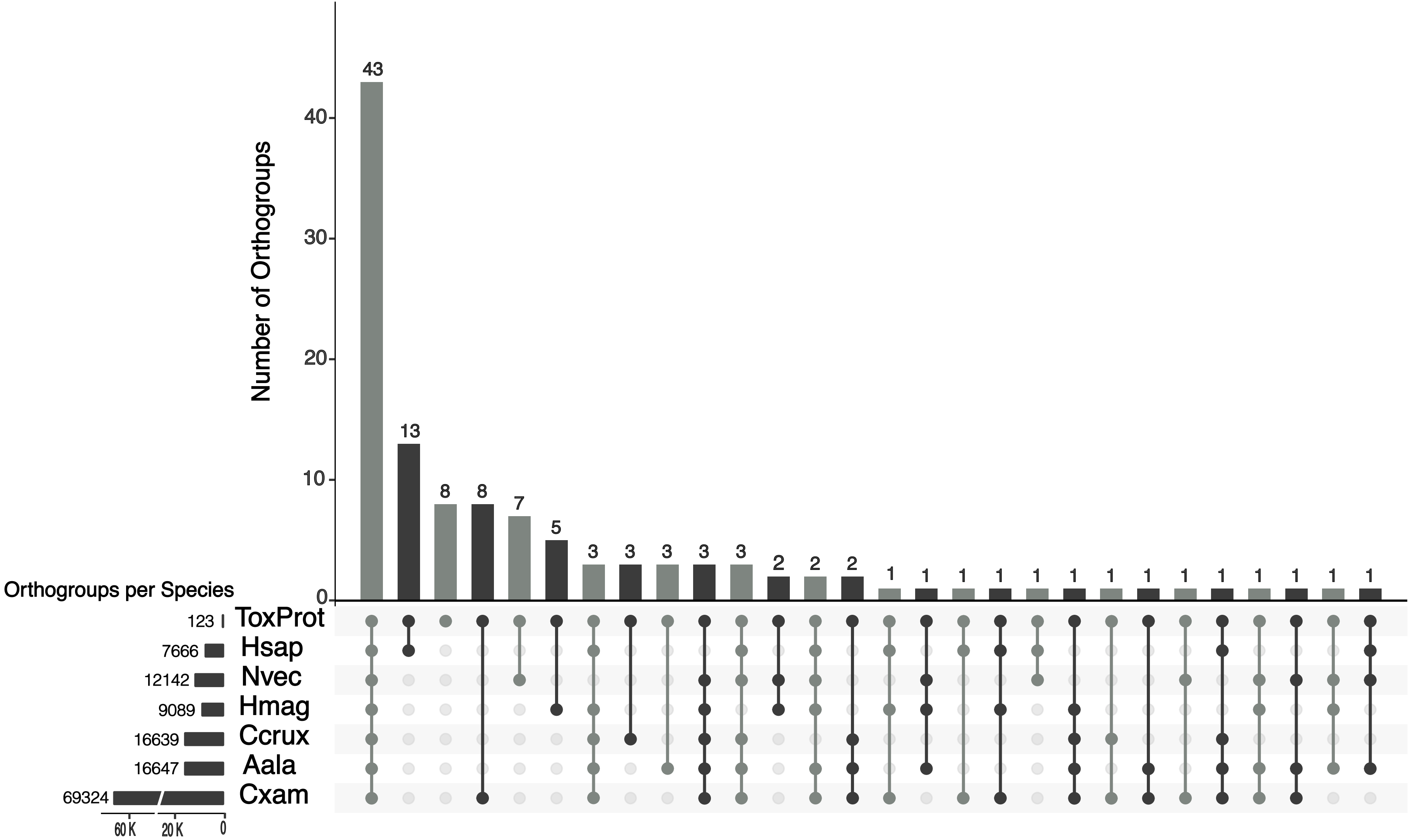

Figure 4

Click here to download Figure Figure4.jpg

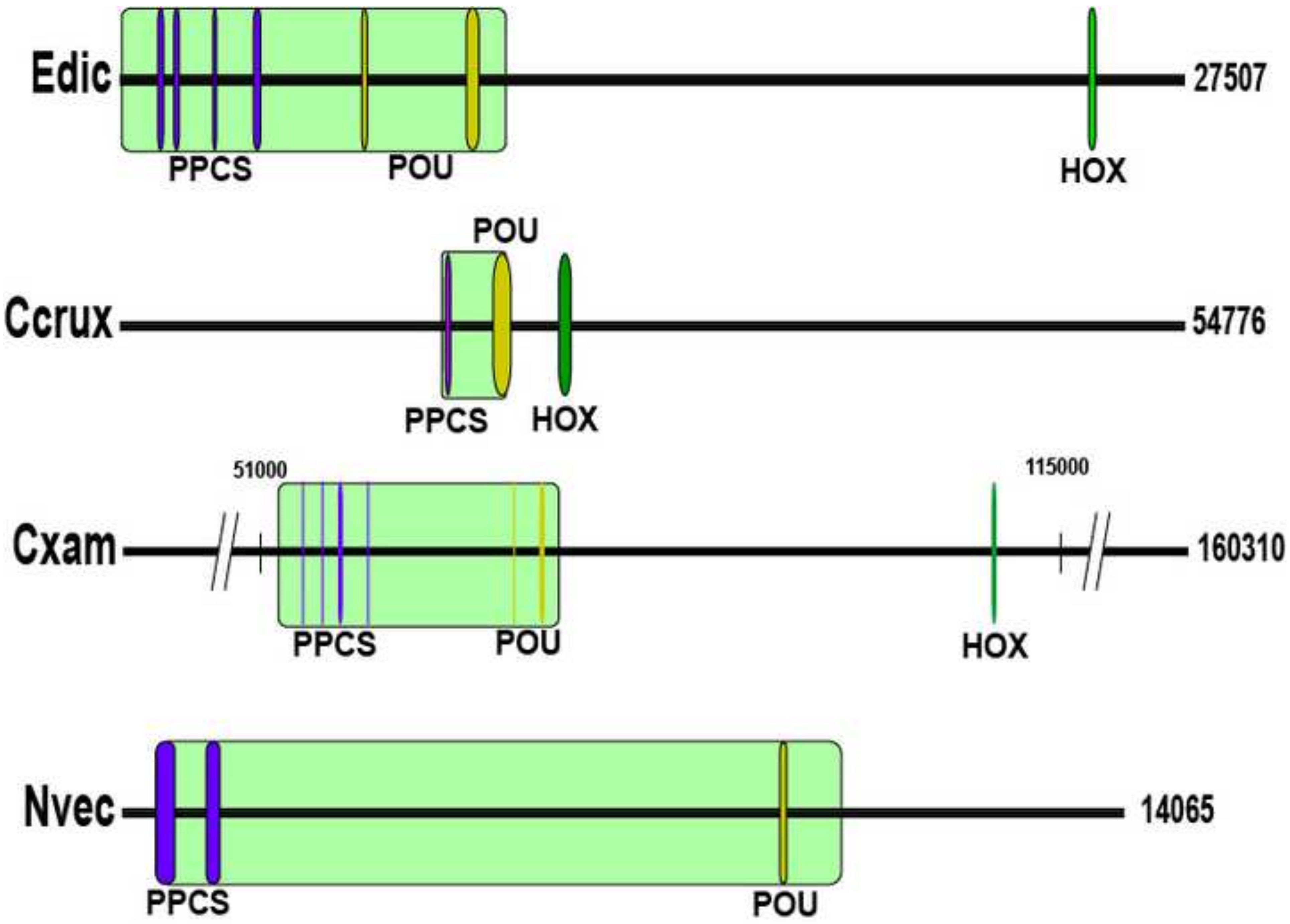

Ccrux_Figure1A

Click here to download Figure luc_crux_17-09-12_1.jpg

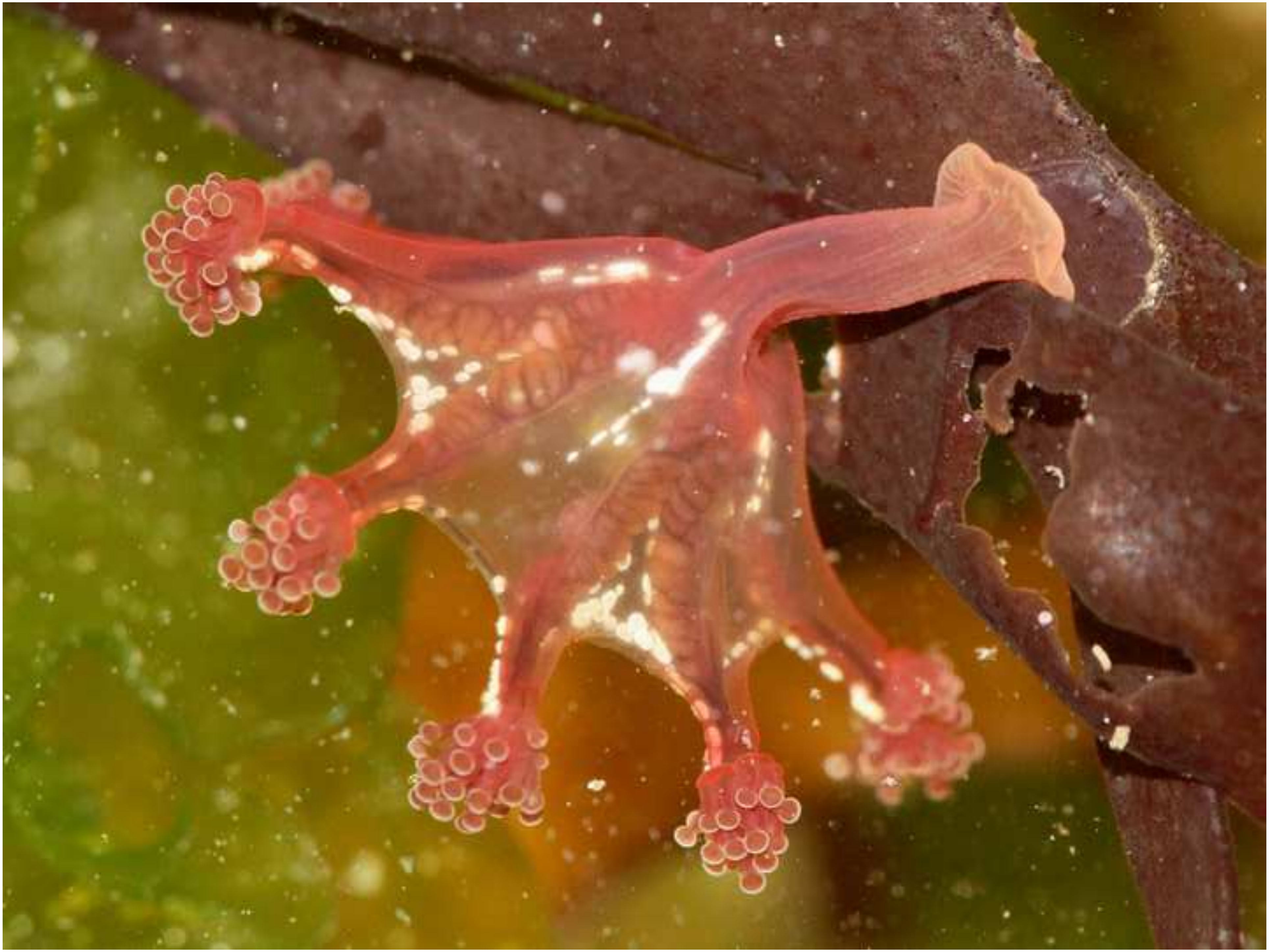

Aala_Figure1B

Click here to download Figure Alatina_alata_AGC_8965_on_black.jpg

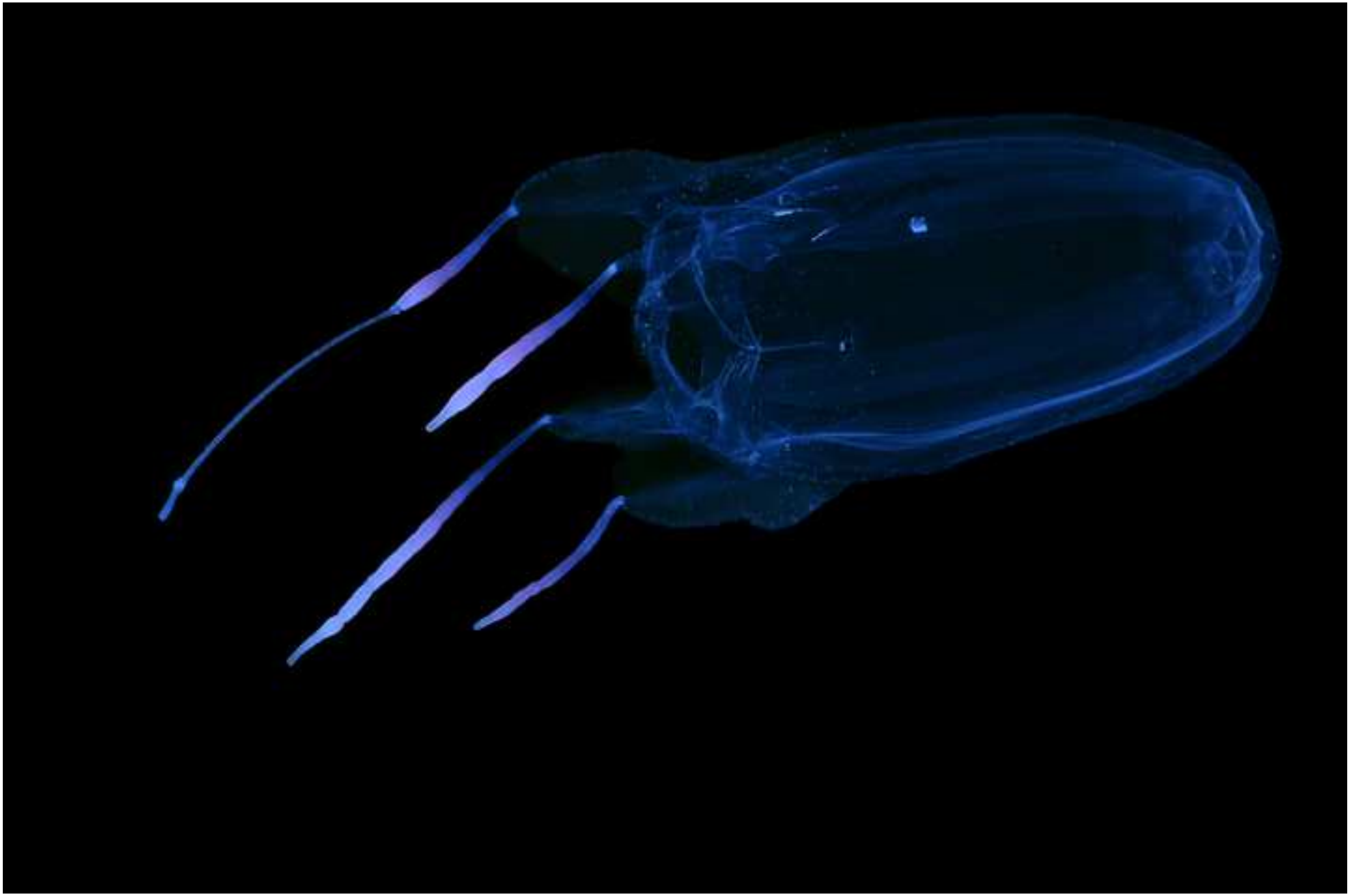

Cxam_Figure1C

Click here to download Figure Cassiopea.jpg

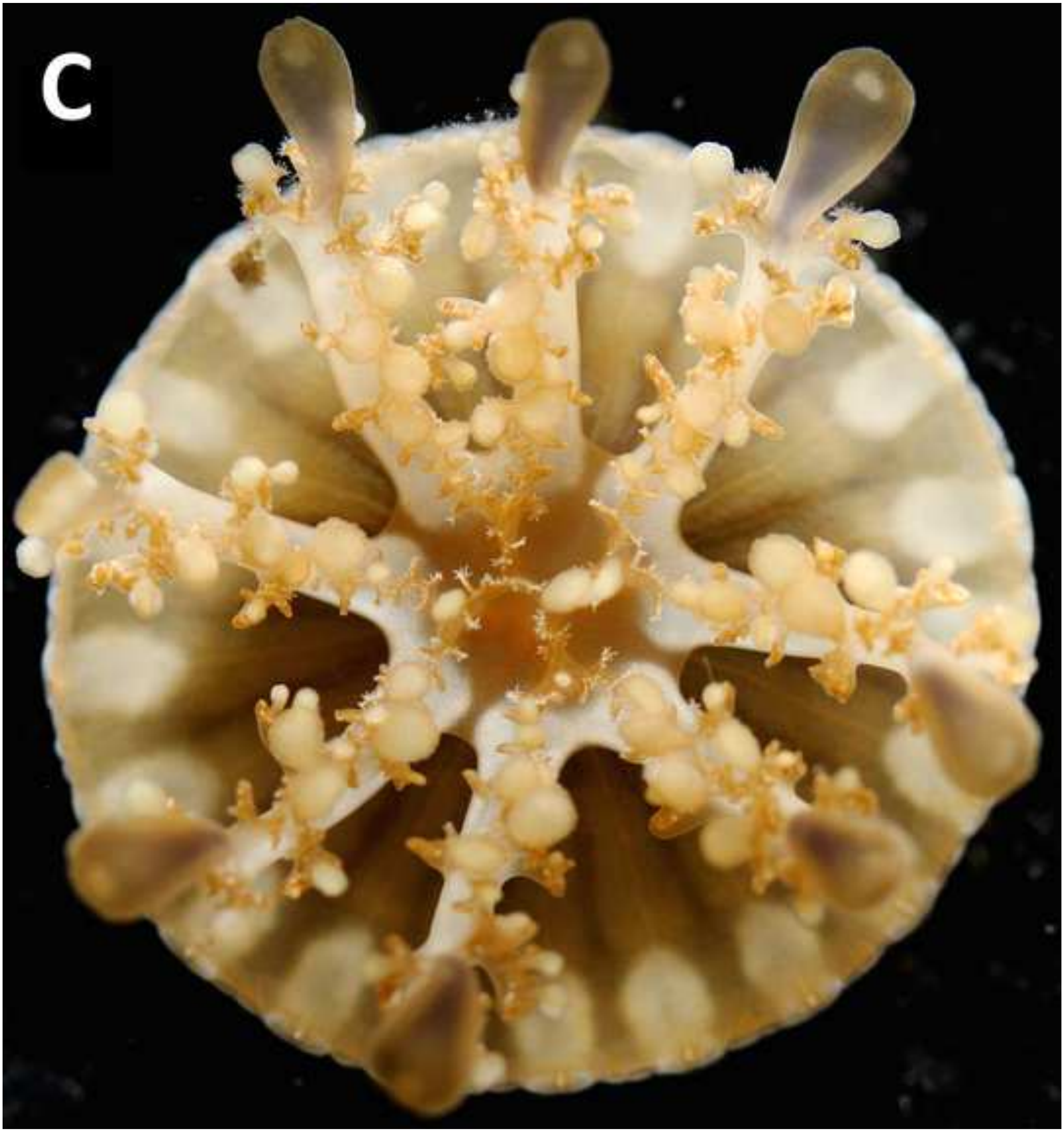

Supplement: giz069_GIGA-D-18-00115_Original_Submission [file giz069_giga-d-18-00115_original_submission.pdf]
