# Supplementary material for: Box, stalked, and upside-down? Draft genomes from diverse jellyfish (Cnidaria, Acraspeda) lineages: Alatina alata (Cubozoa), Calvadosia cruxmelitensis (Staurozoa), and Cassiopea xamachana (Scyphozoa)
Source: Gigascience. 2019 Jul 1;8(7):giz069. doi: 10.1093/gigascience/giz069 (PMC6599738; doi:10.1093/gigascience/giz069)
Supplement: giz069_GIGA-D-18-00115_Revision_1 [file giz069_giga-d-18-00115_revision_1.pdf]

GigaScience

Box, stalked and upside-down? Draft genomes from diverse jellyfish (Cnidaria, Acraspeda) lineages: Alatina alata (Cubozoa), Calvadosia cruxmelitensis (Staurozoa), and Cassiopea xamachana (Scyphozoa)

--Manuscript Draft--

**Manuscript Number:**	GIGA-D-18-00115R1
**Full Title:**	Box, stalked and upside-down? Draft genomes from diverse jellyfish (Cnidaria, Acraspeda) lineages: Alatina alata (Cubozoa), Calvadosia cruxmelitensis (Staurozoa), and Cassiopea xamachana (Scyphozoa)
**Article Type:**	Data Note
**Funding Information:**	University of Florida (US) DSP Research Strategic Initiatives and the Office of the Provost (00114464) — Dr. Joseph F Ryan<br>University of Maryland, Howard J. Brinkley & Eugenie Clark Scholarships — Dr Cheryl Lewis Ames<br>Oakridge Institute for Science and Education's Scientific Visitor Program — Dr Cheryl Lewis Ames, Mr Sean La<br>Pennsylvania State University (Start Up) — Dr Mónica Medina<br>Iridian Genomes — Dr Stacy Pirro<br>National Science Foundation (US), Division of Ocean Sciences (OCE) (1442306) — Dr Mónica Medina<br>National Science Foundation (OCE 1442206) — Dr Mónica Medina
**Abstract:**	Anthozoa, Endocnidozoa, and Medusozoa comprise the three major clades of Cnidaria. Medusozoa is further divided into four clades, Hydrozoa, Staurozoa, Cubozoa, and Scyphozoa—the latter three lineages make up the clade Acraspeda. Acraspeda encompasses extraordinary diversity in terms of life history, numerous nuisance species, taxa with complex eyes rivaling other animals, and some of the most venomous organisms on the planet. Genomes have recently become available within Scyphozoa, but there are currently no published genomes within Staurozoa and Cubozoa. Here we present three new draft genomes of Calvadosia cruxmelitensis (Staurozoa), Alatina alata (Cubozoa), and Cassiopea xamachana (Scyphozoa) for which we provide a preliminary orthology analysis that includes an inventory of their respective venom-related genes. Additionally, we identify synteny between POU and Hox genes that had previously been reported in a hydrozoan, suggesting this linkage is highly conserved, possibly dating back to at least the last common ancestor of Medusozoa, yet likely independent of vertebrate POU-Hox linkages. These draft genomes provide a valuable resource for studying the evolutionary history and biology of these extraordinary animals, and for identifying genomic features underlying venom, vision, and life history traits in Acraspeda.
**Corresponding Author:**	Joseph F. Ryan<br>Whitney Laboratory for Marine Bioscience<br>UNITED STATES
**Corresponding Author Secondary Information:**	
**Corresponding Author's Institution:**	Whitney Laboratory for Marine Bioscience
**Corresponding Author's Secondary Institution:**	
**First Author:**	Aki Hammond Ohdera
**First Author Secondary Information:**	
**Order of Authors:**	Aki Hammond Ohdera

	Cheryl Lewis Ames
	Rebecca B Dikow
	Ehsan Kayal
	Marta Chiodin
	Ben Busby
	Sean La
	Stacy Pirro
	Allen G Collins
	Mónica Medina
	Joseph F Ryan
**Order of Authors Secondary Information:**	
**Response to Reviewers:**	Reviewer reports:<br>Reviewer #1: This is a straightforward report on the generation of 3 acraspedan genomes, the initial annotation steps and the potential further uses of the genomic resources. I believe the genomes were appropriately sequenced from carefully selected samples and I see no major issues with the manuscript at all. The genomes will be immensely important to understand not only patterns of evolution within Cnidaria but also to help clarify other issues in early-splitting lineages. I have only a couple of minor suggestions to the authors and a recommendation. But I believe this manuscript is almost ready for publication.<br><br>1. Please add the information about number of reads and volume of data for Calvadosia<br>- we've added this information to line 197<br><br>2. Their completeness analyses give relatively good numbers for Cassiopea and Calvadosia but very low recovery of eukaryotic and metazoan core genes in Alatina. I think the manuscript would benefit from a comment on why they think they recovered so little.<br>- we've added the following sentence on line 241<br>"The low recovery rates for conserved genes in the A. alata genome are likely due to the considerably larger size of the genome, which tends to be coupled with long introns, and therefore higher rates of gene fragmentation in a draft assembly [80-82].."<br><br>3. Although preliminary, the most interesting aspect of their analyses is the orthology. They detect potential orthologous groups that may represent lineage-specific gains, but they give no details about the genes themselves. Were they identifiable genes in terms of affinity with any other known gene? Could they identify conserved domains? I understand that would deserve a paper themselves, but I think the reader would appreciate more information to understand the importance of these resources.<br>- we've added some gene ontology analysis to give readers some context for the genes that were found (see figures 3-5, supplementary figures 1-2, and line 265-312. We agree with the reviewer that going into more detail is perhaps outside the scope of the giganote.<br><br>Reviewer #2: This manuscript provides a brief description of three draft genomes from medusozoan cnidarians. The genomic resources generated from this study will be very useful to the cnidarian research community. The paper is relatively straightforward, but it lacks a few details that could improve the quality of the contribution. My minor comments are listed below.<br><br>* The introduction could be improved by focusing more on the specific taxa (instead of all of cnidaria), the questions specifically addressed (venom composition, synteny), and the value of these data to the community (i.e. phylogenetic position, filling phylogenetic gaps), instead of a textbook-like introduction to Cnidaria.

	We've added a paragraph which provides details of each taxa with references starting on line 119 - 130.<br><br>* The assembly methods for the three genomes needs to be standardized to include the same information for each of the genomes. For example, why were methods given for mit. Genome assembly for Calvadosia and not the others? Is this relevant to the paper?<br>The genomes were assembled by different team members at different times with different sequence data. Calvadosia was a particularly early assembly and at the time we were experimenting with different ideas on how to improve the assembly. One hypothesis was that the presence of mitochondrial reads, which can be present at much higher levels than nuclear genome, might improve kmer counting methods. This was fairly successful with Calvadosia, but had little effect with several other assemblies we attempted later. We therefore did not apply to Alatina and Cassiopea, and therefore did not assemble those mitochondrial genomes from our data. In addition, MT genomes from Alatina and Cassiopea have been described previously (https://academic.oup.com/gbe/article/4/1/1/536978#89413791). We have added a more brief justification to the text starting on line 141 describing why the methodology is different for each genome.<br><br>* Lines 177 and 210 - were the units for N50 supposed to be kb or was this meant to be bp? What about the N50 unit for Alatina in line 229 (kb or bp?). Also, add the unit to the Table 1.<br>- The units were supposed to be bp. We have corrected this error, and they should now all have bp as the units.<br><br>* More detail for the Alatina assembly is needed. How were the Illumina and PacBio reads treated and combined?<br>- The Alatina reads were assembled by Masurca, which instructs users not to pre-process reads (in the manual). Masurca takes in both long and short-reads for the final assembly. We have added the following sentence starting on line 141: "These three genomes were sequenced over a five-year period, each by different authors, using various technologies and various assembly methods. As such, the methods for each assembly are unique."<br><br>The output of Masurca included a fair amount of adapter that was caught by NCBI during sequence submission. We now mention this in the manuscript starting on line 230: "We conducted hybrid assembly of Illumina short-reads and PacBio long-reads using MaSuRCA 3.2.2 [79] (which includes an error correction step for paired-end reads) that resulted in an assembly of 291,445 contigs and an N50 of 7,049 bp (NCBI Accession PUGI00000000). We did not perform adapter trimming prior to assembly because the MaSuRCA manual advises against preprocessing of reads, including adapter removal. Nevertheless, we identified considerable adapter contamination in our final assembly."<br><br>* Some justification for the assembly methods would be warranted. Why was PacBio performed for Alatina and not the others? And how did (or didn't) PacBio affect the scaffolding? Why was artificial MP performed on Calvadosia and not the others? How did this affect assembly?<br>- The differences in assembly methods are justified now by the statement explaining that sequencing and assembly were performed by different authors. We feel that each of the assembly methods applied are sound and that we do a thorough job of describing each method. We also feel that analyzing these genomes together is worth the slight awkwardness of the assembly-method heterogeneity.<br><br>* A general discussion comparing assembly depth, scaffold # and size and genome completeness of all three genomes would be helpful. What sequencing, assembly methods and scaffolding proved most useful?<br>- We have added a section "Comparison of Assemblies" starting on line 245.<br><br>* Table 2 should be summarized into GO pie-charts or equivalent.<br>- We have moved Table 2 to the supplement and have added Figure 6<br><br>* Figure 2 is a bit difficult to read. It would be helpful it a Venn diagram accompanied

	this figure showing Cnidaria-specific, Medusozoan-specific and Acraspeda-specific orthologous groups.<br>- A six-way comparison will be a challenge to digest in a venn or in the current format. We have added color to group-specific combinations for visual clarification for Figures 2 and 7.<br><br>* An estimate of genomes sizes would be useful information to include.<br>- We now include "estimated genome size" based on the k-mer-based estimates produce by AllPaths-LG in Table 1.<br><br>* Line 312 - the 630-850 million year origin for Medusozoan is not a commonly held estimate. Please use a more reasonable estimate and citation.<br>- We have changed this to "likely lived more than 500 million years."<br><br>* Line 344 - change specious to species-rich (specious means something else).<br>- done.<br><br>* Please cite other recent papers (in addition to Kayal et al, 2018) on cnidarian phylogenomics that contributed much of the data from Kayal et al., 2018.<br>- We now mention Chang et al. (2015) and Zapata et al. (2015) on line 65.
**Additional Information:**	
**Question**	**Response**
Are you submitting this manuscript to a special series or article collection?	No
**Experimental design and statistics**<br><br>Full details of the experimental design and statistical methods used should be given in the Methods section, as detailed in our Minimum Standards Reporting Checklist. Information essential to interpreting the data presented should be made available in the figure legends.<br><br>Have you included all the information requested in your manuscript?	Yes
**Resources**<br><br>A description of all resources used, including antibodies, cell lines, animals and software tools, with enough information to allow them to be uniquely identified, should be included in the Methods section. Authors are strongly encouraged to cite Research Resource Identifiers (RRIDs) for antibodies, model organisms and tools, where possible.<br><br>Have you included the information	Yes

requested as detailed in our Minimum Standards Reporting Checklist?	
**Availability of data and materials**<br><br>All datasets and code on which the conclusions of the paper rely must be either included in your submission or deposited in publicly available repositories (where available and ethically appropriate), referencing such data using a unique identifier in the references and in the “Availability of Data and Materials” section of your manuscript.<br><br>Have you have met the above requirement as detailed in our Minimum Standards Reporting Checklist?	Yes

Powered by Editorial Manager® and ProduXion Manager® from Aries Systems Corporation

Manuscript
Click here to view linked References
Click here to access/download;Manuscript;GigaNote.manuscript_032419.do

Box, stalked and upside-down? Draft genomes from diverse jellyfish (Cnidaria, Acraspeda) lineages: *Alatina alata* (Cubozoa), *Calvadosia cruxmelitensis* (Staurozoa), and *Cassiopea xamachana* (Scyphozoa)

Aki Ohdera[1], Cheryl L. Ames[2,3], Rebecca B. Dikow[4], Ehsan Kayal[2], Marta Chiodin[5], Ben Busby[3], Sean La[3,6], Stacy Pirro[7], Allen G. Collins[2,9], Mónica Medina[1*], Joseph F. Ryan[4*]

1. Department of Biology, Pennsylvania State University, University Park, PA, USA

2. Department of Invertebrate Zoology, National Museum of Natural History, Smithsonian Institution, Washington D.C., USA

3. National Center for Biotechnology Information, Bethesda, MD, USA

4. Data Science Lab, Office of the Chief Information Officer, Smithsonian Institution, Washington D.C., USA

5. Whitney Laboratory for Marine Bioscience, University of Florida, St. Augustine, FL, USA

6. Department of Mathematics, Simon Fraser University, British Columbia, BC, Canada

7. Iridian Genomes, Inc. Bethesda, MD, USA

8. UPMC, CNRS, FR2424, ABiMS, Station Biologique Roscoff, France

9. National Systematics Laboratory of NOAA's Fisheries Service, Washington, DC, USA

* Corresponding authors

Abstract

Anthozoa, Endocnidozoa, and Medusozoa comprise the three major clades of Cnidaria. Medusozoa is further divided into four clades, Hydrozoa, Staurozoa, Cubozoa, and Scyphozoa—the latter three lineages make up the clade Acraspeda. Acraspeda encompasses extraordinary diversity in terms of life history, numerous nuisance species, taxa with complex eyes rivaling other animals, and some of the most venomous organisms on the planet. Genomes have recently become available within Scyphozoa, but there are currently no published genomes within Staurozoa and Cubozoa. Here we present three new draft genomes of *Calvadosia cruxmelitensis* (Staurozoa), *Alatina alata* (Cubozoa), and *Cassiopea xamachana* (Scyphozoa) for which we provide a preliminary orthology analysis that includes an inventory of their respective venom-related genes. Additionally, we identify synteny between POU and Hox genes that had previously been reported in a hydrozoan, suggesting this linkage is highly conserved, possibly dating back to at least the last common ancestor of Medusozoa, yet likely independent of vertebrate POU-Hox linkages. These draft genomes provide a valuable resource for studying the evolutionary history and biology of these extraordinary animals, and for identifying genomic features underlying venom, vision, and life history traits in Acraspeda.

Keywords: Staurozoa, Scyphozoa, Cubozoa, Acraspeda, Cnidaria, Medusozoa

Introduction

Some of the most fascinating and outstanding mysteries related to the genomic underpinnings of metazoan biology are centered around cnidarians [1]. Active areas of research include the basis of venom evolution and diversification [2-4], mechanisms of independent evolution of image-forming vision (lens eyes) [5-7], and the emergence of a pelagic adult stage within a biphasic (or multiphasic) life cycle [8]. Cnidaria encompasses three major clades: Anthozoa, Endocnidozoa, and Medusozoa [9-12]. Anthozoa comprises Hexacorallia and Octocorallia. Hexacorallia includes scleractinian corals, anemones, and zooanthids, and is characterized by a six-fold symmetry, with species exhibiting both colonial and solitary forms. Octocorallia includes sea fans, gorgonians, and soft corals; these animals are characterized by pinnate tentacles in eight-fold symmetry. Endocnidozoa, comprising the parasitic lineages Myxozoa and Polypodiozoa, was only recently properly classified as Cnidaria [13-15]. Medusozoans are characterized by the emergence of a medusa life history stage within some taxa of the clade, their high diversity in regards to life history and morphology, the presence of a linear mitochondrial genome (with a variable number of chromosomes), and by the presence of a hinged cap at the apex of the cnidocyst (cnidarian stinging organelles) [8, 16, 17].

There are approximately 3900 described species within Medusozoa, classified into four diverse lineages: Hydrozoa (hydroids, hydromedusae, siphonophores), Staurozoa (stalked jellyfish), Cubozoa (box jellyfish), and Scyphozoa (true jellyfish) (Figure 1A-C) [1, 11]. There exists much debate regarding the phylogenetic relationships among these lineages [10, 16, 18-20]. Recent phylogenomic analyses have placed Staurozoa as the sister to a clade that contains Cubozoa and Scyphozoa, reuniting these lineages into a group called Acraspeda (Figure 1D) [9, 15, 21]. Given the extensive morphological diversity within Cnidaria, understanding the evolutionary relationships and mechanisms leading to lineage specific innovations has been of considerable interest, but has been fraught with challenges. In

particular, the evolution and subsequent loss of the medusoid form in some lineages hints of a complex evolutionary history within Medusozoa [22].

The mechanisms of medusa formation are variable amongst medusozoans: often involving two phenotypically distinct life stages - polyp and medusa - that are genotypically identical (reviewed in Lewis Ames 2018). Cubozoan polyps undergo partial or complete metamorphosis and develop into the adult medusoid form capable of sexual reproduction, although in some cases a polyp rudiment remains [23]. Scyphozoan polyps (scyphistomae) undergo a transition known as strobilation, in which the upper calyx proceeds through metamorphosis and transverse fission to produce a medusa [24]. Unlike other medusozoans, staurozoans lack a free-swimming medusa stage but exhibit medusa-associated characters that are present in other medusozoans [25]. The basal portion of the adult forms a stalk, or peduncle, while coronal muscles and gastric filaments, among other features, characterizes the apical portion (calyx) of the adult [20, 25-27]. Hydrozoans exhibit the greatest variation in life history strategies and often lack a medusa form [1]. Species that give rise to the medusoid form do so via lateral buds generated by asexual polyps, while others possess sexual polyps without a free-swimming stage [28, 29]. Elsewhere within Cnidaria, Anthozoa and the parasitic Endocnidozoa lack the medusa stage or medusoid characters entirely. Research on medusa development has shown similar gene expression patterns between hydrozoans and scyphozoans, with developmental genes co-opted for patterning the medusa body plan [30, 31]. Interestingly, strobilation in scyphozoans was recently shown to be under the control of the retinoic acid pathway [32-34]; these same genes are involved in metamorphosis of insects and amphibians, hinting towards regulation of metamorphosis being a conserved function in metazoans [35]. The study also found that potential lineage-specific genes were involved in controlling strobilation, suggestive of genomic innovations

within Medusozoa playing a role in medusa morphogenesis, or more specifically within Scyphozoa.

Hox genes, which control body formation during early embryonic development, predate the emergence of both Bilateria and Cnidaria, and the evolution of these genes played a crucial role in the diversification of these lineages [36-38]. In particular, clustering and synteny with non-hox genes has been shown to be important in bilaterians [39], but also in some cnidarians, such as the anthozoan *Nematostella vectensis* [36, 40, 41], and in several hydrozoan species [42-46]. Other than an initial characterization of select Hox genes in *Cassiopea xamachana* [47], information about Hox genes and Hox-gene clustering in Acraspeda species has been limited. In hydrozoans and vertebrates, Hox genes were shown to be linked to another class of homeobox genes, the POU genes [48], but this linkage has not been demonstrated in any other cnidarian lineages. These new Acraspeda genomes provide us with an opportunity to investigate the evolutionary history of the POU-Hox linkage in more detail.

Genomic resources necessary to understand medusozoan evolution have been lacking, with genomes predominantly available for anthozoans and hydrozoans [49-57]. Recently, the genome of *Aurelia aurita* was sequenced, adding to the still-insufficient number of sequenced medusozoan genomes [58]. While the majority of Medusozoa species are represented by hydrozoans (>90%), both cubozoans and scyphozoans garner significant attention as a result of their impact on economy and tourism [1, 59]. Largely due to venom being employed as a mechanisms of defense and prey capture, the inherent risk of jellyfish sting has been exacerbated by uncertainty about how cnidarians will respond to modern-day anthropogenic disturbances [60, 61]. Despite these risks, relatively little is known about cnidarian venom, as compared to snakes, cone snails, and other venomous organisms. Given the great phylogenetic distance between cnidarians and these well-studied venomous organisms, a

better understanding of the cnidarian venom repertoire can provide insight into the evolution of venom and venom-encoding genes.

Here we present three new genomes for species of the three major Acraspeda lineages: *Calvadosia cruxmelitensis* (formerly *Lucernariopsis cruxmelitensis*)(Staurozoa), *Alatina alata* (Cubozoa), and *Cassiopea xamachana* (Scyphozoa). The winged box jellyfish *Alatina alata* (Cnidaria: Cubozoa: Carybdeida: Alatinidae) has been of interest due to its unusual circumtropical distribution [62], extraordinarily rapid gonad development [63], and its reputation as a potent stinger, earning it the honor of being the only jellyfish species to have its own category in US weather reports [64]. The stalked jellyfish *C. cruxmelitensis* has been the recent subject of detailed anatomic [20] and biodiversity studies [27]. The upside-down jellyfish *Cassiopea xamachana* is an established model for understanding cnidarian-dinoflagellate endosymbiosis [65] and, with its ease of culturing and tractability in the laboratory setting, is poised as a model system for evo-devo research and other laboratory-based studies [66].

The genomes and corresponding gene annotations from these three lineages will serve as useful resources aimed at sparking investigative research into the evolution and diversification of life history strategies across cnidarians. Furthermore, future studies examining cnidarian venom evolution, and phylogeographic patterns of venomous jellyfish, may provide opportunities for development of jellyfish-derived therapeutic drugs, and countless additional novel biopharmaceuticals (reviewed in Lewis Ames 2018).

Data Description

Genome Sampling, Sequencing, and Assembly

These three acraspedan genomes were assembled at different times throughout a five-year period as part of several independent projects overseen by the coauthors, using separate methods for collection, extraction, sequencing, and assembly (see below). This valuable resource to the scientific community is the culmination of an extensive collaborative effort to respond to the need for model medusozoan systems in a plethora of research fields.

Cassiopea xamachana Sample Collection and DNA extraction

We propagated *C. xamachana* polyps from a single polyp via asexual budding (Line T1-A). Polyps were maintained symbiont-free at 26 °C, and fed 3 times weekly with *Artemia* nauplii. To avoid the possibility of food-source contaminates interfering with downstream bioinformatic analysis, we starved the polyps for seven days in antibiotic-treated seawater prior to preservation in 95% ethanol; any *Artemia* cysts retained within the gut were manually removed before preservation. We extracted genomic DNA from the apo-symbiotic (lacking endosymbionts) polyps using a CTAB (cetyl trimethylammonium bromide) phenol chloroform extraction, first performing an overnight digestion of whole polyp tissue with proteinase K (20 mg/ml) in CTAB buffer before proceeding with the standard protocol. DNA extract was stored at -20 °C until further processing.

Calvadosia cruxmelitensis Sample Collection and DNA extraction

We collected adult specimens of *C. cruxmelitensis* in January 2013 at Chimney Rock, off the coast of Penzance, Cornwall, England. Specimens were immediately preserved in ethanol and stored at -20 °C until further processing. We extracted genomic DNA using a phenol-choloroform protocol in an Autogen mass extractor, and stored the DNA extract at -20 °C until further processing.

***Alatina alata* Sample Collection and DNA extraction**

We collected *A. alata* material during a spermcasting aggregation in Bonaire, The Netherlands (April, 2014, 22:00-01:00) according to the methods in Lewis Ames et al. [7]. We selected a single live spermcasting male medusa from the same cohort as the female medusa used previously published RNA-Seq studies (Genbank Accession: GEUJ01000000) [7, 9]. The medusa was divided into four longitudinal sections, and one quarter was placed into a 15 ml tube with pure (99%) ethanol, flash-frozen at -180 °C (using a dry shipper), and subsequently transported to the Smithsonian NMNH and stored at -20 °C. We extracted genomic DNA using a DNeasy Blood & Tissue Kit (Qiagen), following the manufacturer's protocol. DNA extract was stored at -20 °C until further processing.

***Cassiopea xamachana* Sequencing and Assembly**

Library construction and sequencing was performed at HudsonAlpha Institute for Biotechnology. Four 350 bp paired-end linear libraries with insert sizes of 500 bp were generated with Illumina TruSeq DNA PCR-Free LT Prep Kits and sequenced on the Illumina Hiseq2000. Approximately 634 million reads totaling 117.6 Gb of high-quality paired-end sequence data were generated. We performed adaptor trimming and quality filtering using Trimmomatic v0.36 [67] with default settings, followed by genome size estimation and error correction with Allpaths-LG version 52488 [68]. We removed mitochondrial reads using FastqSifter v1.1.1 (https://doi.org/10.5281/zenodo.1211357) using the *C. xamachana* mitochondrial genome as a reference (NCBI NC_016466.1). We performed *de novo* genome assemblies using ABySS 2.0.1 with default settings [69], SPAdes genome assembler v3.10.0 [70], and Platanus version 1.2.1 (with default parameters, k=89) [71] (Table 1). We used a custom Perl script, plat.pl, (https://github.com/josephryan/Ohdera_et_al_2018) to invoke the Platanus commands for assembly, scaffolding, and gap closing. Of the three assembly methods, Platanus produced the best draft assembly with 93,483 scaffolds measuring a total

of 393.5 Mb with an N50 of 15,563 bp (Table 1) (ENA Accession OLMO01000000). We recovered 82.66% (53.63 % complete and 29.03 % partial) of the core eukaryotic genes and 66.97% (58.59 % complete and 8.38 % partial) of the core metazoan genes with CEGMA ver. 2.5 [72] and BUSCO ver.2.01 [73], respectively, through the gVolante web server [74] (Table 1).

***Calvadosia cruxmelitensis* Sequencing and Assembly**

Library construction and sequencing for *C. cruxmelitensis* were performed at the University of Florida Interdisciplinary Center for Biotechnology Research. Four 150 bp paired-end linear libraries and four 150 bp single-end linear libraries with insert size of 300 bp were generated and sequenced on the Illumina NextSeq 500, generating 291,944,064 paired-end reads and 141,911,072 single-end reads. We performed adaptor trimming and quality filtering using Trimmomatic-0.32 [67] with default settings, followed by genome size estimation error correction using Allpaths-LG v.44837 [68]. We removed mitochondrial sequences to improve the final assembly with FastqSifter v1.1.1 (https://github.com/josephryan/FastqSifter, https://doi.org/10.5281/zenodo.1211357), using a *de novo* assembly of the *C. cruxmelitensis* mitochondrial genome. We assembled the *C. cruxmelitensis* mitochondrial genome by capturing contigs from an initial assembly using available staurozoan mitochondrial DNA sequences from NCBI as a reference, following the methods presented in Kayal et al. [75], using Geneious v9.0 to generate the final mitochondrial assembly. We checked completeness of the mitochondrial genome using NCBI BLAST against the nr database in addition to a manually generated set of medusozoan genes, annotated tRNA genes separately by using tRNAscan-SE [76] and Arwen [77], and checked the integrity of the assembly by aligning the reads to the completed mitochondrial genome. With the mitochondrial sequences removed, we generated two "sub-optimal" assemblies using Platanus v1.2.1 with kmer size of 32 bp and 45 bp and default settings. Subsequently,

we used these "sub-optimal" assemblies to construct artificial mate-pair libraries for 9 insert sizes (1000, 2000, 3000, 4000, 5000, 7500, 10000, 15000, 20000) with MateMaker (https://github.com/josephryan/matemaker). We used the artificial mate-pair libraries to scaffold the optimal assembly (generated using Platanus kmer=45) with SSPACE Standard v3.0 [78]. This process produced a draft assembly with 417,008 scaffolds measuring a total of 209.3 Mb with an N50 of 16,443 bp (Table 1) (ENA Accession OFHS01000000). We recovered 91.94 % (61.29 % complete and 30.65 % partial) of the core eukaryotic genes and 85.07% (70.86 % complete and 14.21 % partial) of the core metazoan genes with CEGMA and BUSCO, respectively.

***Alatina alata* Sequencing and Assembly**

Illumina library prep and sequencing was conducted at the University of Kansas Genome Sequencing Core. Libraries were generated with the Illumina Nextera Library Preparation kit and sequenced twice on the Illumina HiSeq 2500. The two different runs were performed on the same library: one with 100 bp paired-end, and one with 150 bp paired-end sequencing, resulting in 564 million reads totaling 148.6 Gb of paired-end sequence data. PacBio library prep and sequencing were completed at the University of Washington Northwest Genomics Center. We constructed the libraries with unsheared DNA with end-cleanup only, with an average insert size of 6000 bp. Sequencing was completed on the PacBio RS II platform, resulting in 486,000 long-reads totaling 990.2 Mb of data. We conducted hybrid assembly of Illumina short-reads and PacBio long-reads using MaSuRCA 3.2.2 [79] (which includes an error correction step for paired-end reads) that resulted in an assembly of 291,445 contigs and an N50 of 7,049 bp (NCBI Accession PUGI00000000). We did not perform adapter trimming prior to assembly because the MaSuRCA manual advises against preprocessing of reads, including adapter removal. Nevertheless, we identified considerable adapter contamination in our final assembly. Subsequently, we used a custom

script (remove_adapters_and_200.pl https://github.com/josephryan/Ohdera_et_al_2018) to remove adapters and sequences shorter than 200 nucleotides. The total length of the assembly was 851.1 Mb. We recovered 29.84 % (8.06 % complete and 21.78 % partial) of the core eukaryotic genes and 32.11% (18.30 % and 13.81 % partial) of the core metazoan genes with CEGMA and BUSCO, respectively. The low recovery rates for conserved genes in the *A. alata* genome are likely due to the considerably larger size of the genome, which tends to be coupled with long introns, and therefore higher rates of gene fragmentation in a draft assembly [80-82].

Comparison of Assemblies

A comparison of the draft genomes assembled in this study reveals that the genome of *Alatina alata* is four times the size of that of *C. cruxmelitensis* and almost twice the size of the genome of *Cassiopea xamachana*. The apparent contiguity of the assemblies is reflected in this size difference, with the N50 of the *A. alata* genome (7,049 bp) being considerably smaller for both *C. cruxmelitensis* (16,443 bp) and *Cassiopea xamachana* (15,563 bp). The N50, the minimum length of at least half the contigs/scaffolds in an assembly, tends to scale with the level of completeness as measured by CEGMA and BUSCO. For example, CEGMA recovered 91.94 % of 248 conserved eukaryotic genes (complete + partial) in *C. cruxmelitensis* and 82.66 % in *Cassiopea xamachana*, and 29.84 % in *A. alata* (Table 1).

Gene Model Prediction

We predicted genes for all three genomes using Augustus v3.2.2 [83], with the *Homo sapiens* training set and hits generated with BLAT [84] alignments of published transcriptome data (*C. cruxmelitensis* ENA accession= HAHC01000000; *C. xamachana* ENA accession= PRJEB21012; *A. alata* accession= PRJNA312373) to the genome assemblies of the respective taxa [11]. The *Homo sapiens* training set was used because

Augustus gene predictions using the *Nematostella vectensis* v1.0 training set failed to detect intronic regions within predicted genes, thereby resulting in predicted proteins consisting of single exons. We generated 66,156 gene models for *A. alatina*, 26,258 for *C. cruxmelitensis* and 31,459 for *Cassiopea xamachana*.

Gene Orthology and Lineage-Specific Gene Ontology

We used OrthoFinder v1.1.4 [85] to construct orthologous groups between gene models of *A. alatina*, *C. cruxmelitensis*, *Cassiopea xamachana*, *N. vectensis*, *Hydra magnipapillata,* and *Homo sapiens.* We also included translated transcriptome assemblies for *A. alatina*, *C. cruxmelitensis*, and *Cassiopea xamachana* in these ortholog analyses, as well as an additional transcriptome of the apo-symbiotic polyp stage of *C. xamachana*, which was assembled using Trinity v2.4.0 [86] with default settings (ENA Project Accession: PRJEB23739). All transcriptomes were translated using TransDecoder 3.0.0 [87] with minimum protein length (-m) set to 50 and all other settings as default. We annotated orthogroups by BLASTing a representative species against the Uniprot/Swiss-Prot database [88]. Orthogroups with annotations were further mapped to Gene Ontology terms, and analyzed for putative enrichment related to biological function using ClusterProfiler [89], against a *C. xamachana* annotation database generated using AnnotationForge [90].

Our OrthoFinder analysis generated a total of 80,482 orthogroups for the combined genomic datasets. Using a custom script, we identified 756 Cnidaria-specific orthogroups, another 562 medusozoan-specific orthogroups, and yet another 1091 Acraspeda-specific orthogroups (Figure 2); genes in each taxon-specific orthogroup were non-overlapping. Of these unique orthogroups, we were able to retrieve Swiss-Prot annotations for 57% of Cnidaria-specific orthogroups, 32% of Medusozoa-specific orthogroups, and 55% of Acraspeda-specific orthogroups (Table S1-S3). Unannotated orthogroups may represent taxonomically restricted genes or genes no longer discernable as such due to possibly

extensive genetic mutation experienced in evolutionary history. Using this framework, we identified enriched GO (gene ontology) terms, uncovering 123 terms corresponding to biological process that appear to be enriched within Cnidaria: 107 for Medusozoa, and 14 for Acraspeda (adjusted p-value < 0.01). We used ReViGO to remove redundant GO terms from these initial lists and grouped them further through k-means clustering by Euclidean distance. The optimal number of clusters was predicted using the R package NbClust v3.0.

Our ReViGO analysis reduced the 123 Cnidaria-specific GO terms for biological processes to 59 non-redundant ReViGO terms comprising 5 clusters (Figure 4 Table S1, Figure S1). Within the five clusters, many genes putatively encoding proteins for the cnidarian nerve net were represented (e.g., development and sensory perception) indicative of a system exhibiting a complex response to physical and chemical stimuli. Additionally, terms related to transport (ion, amines, carbon compounds) and the extracellular matrix were also represented. Genes associated with the extracellular matrix are possibly linked to the cnidarian novelty, the mesoglea (the proteinaceous layer between the endoderm and ectoderm in these diploblastic animals). 107 Medusozoa-specific GO terms were reduced to 41 non-redundant ReViGO terms, which when grouped by k-means formed 3 clusters (Figure 7, Table S2, Figure S2). Similar to terms represented within Cnidaria, Medusozoa terms were also associated with response to stimuli and neural. Unique terms seemingly important to medusozoan biology were those related to wound healing and tissue migration, as well as apoptotic signaling regulation. These terms are possibly associated with unique asexual reductive traits (budding, fission, strobilation, etc.) seen within the medusozoan lineage. Despite initially identifying 1091 orthogroups unique to Acraspeda, only 14 GO terms were enriched (highly abundant); this number was further reduced to 8 non-redundant ReViGO terms (Figure 5, Table S3,). The apparently low enrichment may reflect an under-

representation of Acraspeda genes within the reference database. Interestingly, half of the terms were associated with DNA recombination.

Venom Analysis

We identified potential venom-encoding genes within the cnidarian transcriptomes using the venomix database (a publicly available curated set of 6,622 venom-related proteins) and associated pipeline [91]. Additional venom-encoding genes were identified by BLASTing the >6000 venom-related protein sequences of the venomix database to the Augustus protein predictions (BLAST v2.2.31+ e-value = 10^{-6}) [92]. By combining the results of both approaches, we identified 93 types of venom-encoding genes in *C. cruxmelitensis*, 93 in *A. alata*, 97 in *Cassiopea xamachana*, 96 in *H. magnipapillata*, and 91 in the *N. vectensis*. In total, we identified 117 types of putative venom proteins, organized into 32 families, that were present in at least one of the five cnidarian taxa (Figure 6, Table S4). To attempt to reconstruct evolutionary relationships among venom proteins in cnidarians, we added the venomix database to our initial set of input protein sequences and reran our OrthoFinder pipeline. Using this process, we identified 124 orthogroups encoding venom genes in the five cnidarian genomes and the human genome (Figure 7). Of the 124 venom orthogroups, few were found to be specific to any one cnidarian lineage, with five orthogroups present across all cnidarians, two spanning medusozoans, and one shared between Acraspeda. Most of the proteins in the venomix database were identified first in bilaterian animals, and properly curated based on extensive supporting data, whereas putative toxins identified in non-model cnidarians often lack robust evidence to support annotations, precluding their entry into curated databases; hence the limited number of proteins returned in our homology search. However, we were successful in identifying nine cnidarian-specific toxin proteins [4, 93-96]. Four of these proteins (potassium channel toxin BcsTx, peptide toxin Am-1, AvTx, MsepPTx) were found exclusively in *N. vectensis*, while CqTX was

exclusive to the genome of *A. alatina*. Interestingly, CrTx, a toxin previously identified in Cubozoa, including *A. alatina* [95, 97], was also found in *C. xamachana* and *H. magnipapillata.* While the genome of *A. alatina* appears to possess five copies of the CrTx gene, we identified three putative copies in *C. xamachana* and one copy in *H. magnipapillata* genomes, respectively. However, CrTx was absent from both the *C. cruxmelitensis* genome and transcriptome, suggesting this gene may have been lost from the staurozoan lineage. Additionally, the pore-forming toxins (PFT) sticholysin and hydralysin [93, 98] were found only in the *H. magnipapillata* genome in our analysis. Sticholysin was originally identified in the sea anemone *Stichodactyla helianthus*, but its absence from the *N. vectensis* genome may indicate that it is not an Anthozoa-specific protein, but rather variably distributed in Cnidaria.

Hox-POU synteny analysis

In the hydrozoan *Eleutheria dichotoma*, a POU6 class homeobox gene is fused with a phosphopantothenoylcysteine-synthetase (PPCS), and this PPCS/POU6 fusion is linked to a Hox class homeobox gene, Cnox5 [48]. The PPCS/POU6 fusion is known only in Cnidaria and its presence in the anthozoan *Nematostella vectensis* suggests it was likely present in the last common cnidarian ancestor. On the other hand, PPCS/POU6 fusion is not linked to a Hox class gene in *N. vectensis* (cf. Putnam et al. 2007 assembly [56]) suggesting POU-Hox linkage might be a more recent event. Of the Acraspeda genomes we find the PPCS/POU6 fusion gene linked to a Cnox5 ortholog in *C. cruxmelitensis* and *Cassiopea xamachana* (Figure 8, Figure S3). Considering that the last common medusozoan ancestor likely lived more than 500 million years ago [99], it is reasonable to conclude that a functional constraint has led to conserved synteny for PPCS/POU6 fusion. However, the linkage to Cnox5 was not recovered in the *A. alata* genome, preventing further speculation about whether POU-Hox linkage was present in the last common acraspedan ancestor.

Based on the well-established linkage of a POU class homeobox gene to Hox clusters in vertebrates [48], it had been suggested that a POU-Hox linkage may have been present in the last common ancestor of cnidarians and bilaterians. To check this, we searched several additional anthozoan genomes: *Stylophora pistillata* [54], *Acropora digitifera* [100] as well as several invertebrate bilaterian genomes: *Capitella teleta* (Polychaeta) [101], *Strigamia maritima* (Chilopoda) [102], *Octopus bimaculoides* (Cephalopoda) [103], *Mizuhopecten yessoensis* (Bivalvia) [104], and *Ciona intestinalis* (Ascidiacea) [105] that were not available at the time of the original study. We found no evidence for ancient POU-Hox synteny in these anthozoans nor in the invertebrate bilaterian genomes, suggesting that the POU-Hox linkage in medusozoans was achieved independently from the vertebrate POU-Hox linkage (Figure 8, Figure S3). These findings demonstrate how the three new medusozoan genomes allow us to address questions pertaining to molecular evolution, as well as the synergistic benefit of increased genomic-level taxon sampling when testing hypotheses about ancestral states.

Conclusions

In this note we describe draft genomes for three species of the medusozoan sub-group Acraspeda (Cnidaria) – *Calvadosia cruxmelitensis* (Staurozoa), *Alatina alata* (Cubozoa), and *Cassiopea xamachana* (Scyphozoa) – and our corresponding bioinformatics workflows for their assemblies and partial annotations. The findings of our preliminary orthology analyses and annotation of Hox-linked and venom-related genes provide a glimpse into genetic components underlying the evolution of certain traits in these early metazoans. Coupled with appropriate bioinformatics tools and data management pipelines, researchers across a broad range of scientific fields can utilize these resources to investigate the genetic basis of defense, reproduction, and communication in this ancient and species-rich group that encompasses a diversity of life histories, some of which exhibit pelagic life stages. Furthermore, cnidarian

genomes offer strategic opportunities to investigate possible genetic links to any number of ecological issues related to jellyfish that are frequently reported in the scientific literature, or in news media.

These medusozoan genomes will be useful resources in developing functional constructs (e.g. CRISPR/Cas9 guide RNAs) that can be employed to understand the genomic basis for some of the captivating biological innovations of these animals, and eventually for the design of probes for target-capture DNA sequencing. Lastly, the availability of these genomic-level sequence data is an important step forward in the pursuit to elucidate evolutionary events that may have shaped Medusozoa, and in reconstructing the last common ancestor of Cnidaria and Bilateria. Therefore, we are confident that these new genomes will prove valuable for understanding the biology of these fascinating creatures, and for exploring key genomic events that were formative in the early evolution of animals.

Availability of supporting Data

Accession numbers for raw sequencing reads and assemblies are available in Table 1. Custom scripts and parameters used for the analyses are available at https://github.com/josephryan/Ohdera_et_al_2018.

Author Contributions

Alatina alata samples were collected by CLA, AGC and SP. *Alatina alata* assembly: RD and CLA with BB and SL; *C. xamachana* assembly: AO and JFR; *Calvadosia cruxmelitensis* assembly: MC and JR. *Calvadosia cruxmelitensis* mitochondrial genome assembly: EK. Orthology analyses: AO and JR. The manuscript was drafted by AO with substantial contributions from CLA, AGC, and JFR. All coauthors read and provided feedback on the final manuscript. SP, AGC, MM, and JR oversaw the project from start to finish.

Funding

The sequencing of *Alatina alata* was funded by Iridian Genomes. AO acknowledges funding from NSF Dimensions Grant (OCE 1442206); CLA acknowledges funding from The University of Maryland Eugenie Clark Scholarship and a fellowship from Oak Ridge Institute for Science and Education (ORISE); startup funds from the University of Florida DSP Research Strategic Initiatives #00114464 and University of Florida Office of the Provost Programs to J.F.R..

References

1. Lewis Ames C. Medusa: A Review of an Ancient Cnidarian Body Form. In: M. K and J. K, editors. Marine Organisms as Model Systems in Biology and Medicine Results and Problems in Cell Differentiation. Cham, Switzerland: Springer; 2018. p. 105-36.
2. Brinkman DL and Burnell JN. Biochemical and molecular characterisation of cubozoan protein toxins. Toxicon. 2009;54 8:1162-73. doi:10.1016/j.toxicon.2009.02.006.
3. Jouiaei M, Sunagar K, Federman Gross A, Scheib H, Alewood PF, Moran Y, et al. Evolution of an Ancient Venom: Recognition of a Novel Family of Cnidarian Toxins and the Common Evolutionary Origin of Sodium and Potassium Neurotoxins in Sea Anemone. Molecular Biology and Evolution. 2015;32 6:1598-610. doi:10.1093/molbev/msv050.
4. Jouiaei M, Yanagihara AA, Madio B, Nevalainen TJ, Alewood PF and Fry BG. Ancient Venom Systems: A Review on Cnidaria Toxins. Toxins (Basel). 2015;7 6:2251-71. doi:10.3390/toxins7062251.
5. Coates MM. Visual ecology and functional morphology of cubozoa (cnidaria). Integr Comp Biol. 2003;43 4:542-8. doi:10.1093/icb/43.4.542.
6. Liegertova M, Pergner J, Kozmikova I, Fabian P, Pombinho AR, Strnad H, et al. Cubozoan genome illuminates functional diversification of opsins and photoreceptor evolution. Sci Rep. 2015;5:11885. doi:10.1038/srep11885.
7. Lewis Ames C, Ryan JF, Bely AE, Cartwright P and Collins AG. A new transcriptome and transcriptome profiling of adult and larval tissue in the box jellyfish Alatina alata: an emerging model for studying venom, vision and sex. BMC Genomics. 2016;17:650. doi:10.1186/s12864-016-2944-3.
8. Collins AG. Phylogeny of Medusozoa and the evolution of cnidarianlife cycles. Journal of Evolutionary Biology. 2002;15:418-32.

9. Zapata F, Goetz FE, Smith SA, Howison M, Siebert S, Church SH, et al. Phylogenomic Analyses Support Traditional Relationships within Cnidaria. PLoS One. 2015;10 10:e0139068. doi:10.1371/journal.pone.0139068.
10. Marques AC and Collins AG. Cladistic analysis of Medusozoa and cnidarian evolution. Invertebrate Biology. 2004;123 1:23-42.
11. Kayal E, Bentlage B, Sabrina Pankey M, Ohdera AH, Medina M, Plachetzki DC, et al. Phylogenomics provides a robust topology of the major cnidarian lineages and insights on the origins of key organismal traits. BMC Evolutionary Biology. 2018;18 1 doi:10.1186/s12862-018-1142-0.
12. Holzer AS, Bartosova-Sojkova P, Born-Torrijos A, Lovy A, Hartigan A and Fiala I. The joint evolution of the Myxozoa and their alternate hosts: A cnidarian recipe for success and vast biodiversity. Mol Ecol. 2018;27 7:1651-66. doi:10.1111/mec.14558.
13. Siddall ME, Martin DS, Bridge D, Desser SS and Cone DK. The demise of a phylum of protists: phylogeny of Myxozoa and other parasitic cnidaria. J Parasitol. 1995;81 6:961-7.
14. Holland JW, Okamura B, Hartikainen H and Secombes CJ. A novel minicollagen gene links cnidarians and myxozoans. Proc Biol Sci. 2011;278 1705:546-53. doi:10.1098/rspb.2010.1301.
15. Chang ES, Neuhof M, Rubinstein ND, Diamant A, Philippe H, Huchon D, et al. Genomic insights into the evolutionary origin of Myxozoa within Cnidaria. Proc Natl Acad Sci U S A. 2015;112 48:14912-7. doi:10.1073/pnas.1511468112.
16. Bridge D, Cunningham CW, Schierwater B, DeSalle R and Buss LW. Class-level relationships in the phylum Cnidaria- Evidence from mitochondrial genome structure. Proceedings of the National Academy of Science. 1992;89:8750-3.
17. Reft AJ and Daly M. Morphology, Distribution, and Evolution of Apical Structure of Nematocysts in Hexacorallia. Journal of Morphology. 2012;273:121-36.
18. von Salvini-Plawen L. On the origin and evolution of the lower Metazoa. Zeitscrhift Fur Zoologische Systematik und Evolutionsforschung. 1978;16:40-88.
19. Ortman BD, Bucklin A, Pagès F and Youngbluth M. DNA Barcoding the Medusozoa using mtCOI. Deep Sea Research Part II: Topical Studies in Oceanography. 2010;57 24-26:2148-56. doi:10.1016/j.dsr2.2010.09.017.
20. Miranda LS, Hirano YM, Mills CE, Falconer A, Fenwick D, Marques AC, et al. Systematics of stalked jellyfishes (Cnidaria: Staurozoa). PeerJ. 2016;4:e1951. doi:10.7717/peerj.1951.
21. Kayal E, Roure B, Philippe H, Collins AG and Lavrov DV. Cnidarian phylogenetic relationships as revealed by mitogenomics. BMC Evolutionary Biology. 2013;13 5:1.
22. Cartwright P and Nawrocki AM. Character evolution in Hydrozoa (phylum Cnidaria). Integrative and comparative biology. 2010;50 3:456-72. doi:10.1093/icb/icq089.
23. Toshino S, Miyake H, Ohtsuka S, Adachi A, Kondo Y, Okada S, et al. Monodisc strobilation in Japanese giant box jellyfish Morbakka virulenta (Kishinouye, 1910): a strong implication of phylogenetic similarity between Cubozoa and Scyphozoa. Evolution & Development. 2015;17 4:231-9. doi:10.1111/ede.12127.
24. Helm RR. Evolution and development of scyphozoan jellyfish. Biological Reviews. 2018;93 2:1228-50. doi:10.1111/brv.12393.

25. Miranda LS, Collins AG, Hirano YM, Mills CE and Marques AC. Comparative internal anatomy of Staurozoa (Cnidaria), with functional and evolutionary inferences. PeerJ. 2016;4:e2594. doi:10.7717/peerj.2594.
26. Kikinger R and von Salvini-Plawen L. Development From Polyp to Stauromedusa in Stylocoronella (Cnidaria: Scyphozoa). Journal of the Marine Biological Association of the United Kingdom. 2009;75 04:899. doi:10.1017/s0025315400038236.
27. Miranda LS, Mills CE, Hirano YM, Collins AG and Marques AC. A review of the global diversity and natural history of stalked jellyfishes (Cnidaria, Staurozoa). Marine Biodiversity. 2017; doi:10.1007/s12526-017-0721-4.
28. Boero F, Bouillon J, Piraino S and Schmid V. Diversity of hydroidomedusan life cycles- ecological implications and evolutionary patterns. Proceedings of the 6th International Conference on Coelenterate Biology. 1997:56-62.
29. Bentlage B, Osborn KJ, Lindsay DJ, Hopcroft RR, Raskoff KA and Collins AG. Loss of metagenesis and evolution of a parasitic life style in a group of open ocean jellyfish. Molecular Phylogenetics and Evolution. 2018;123:50-9. doi:10.1016/j.ympev.2018.02.030.
30. Reber-Muller S, Streitwolf-Engel R, Yanze N, Schmid V, Stierwald M, Erb M, et al. BMP2/4 and BMP5-8 in jellyfish development and transdifferentiation. The Internatonal Journal of Developmental Biology. 2006;50 4:377-84. doi:10.1387/ijdb.052085sr.
31. Kraus JE, Fredman D, Wang W, Khalturin K and Technau U. Adoption of conserved developmental genes in development and origin of the medusa body plan. Evodevo. 2015;6:23. doi:10.1186/s13227-015-0017-3.
32. Fuchs B, Wang W, Graspeuntner S, Li Y, Insua S, Herbst EM, et al. Regulation of polyp-to-jellyfish transition in *Aurelia aurita*. Current biology : CB. 2014;24 3:263-73. doi:10.1016/j.cub.2013.12.003.
33. Brekhman V, Malik A, Haas B, Sher N and Lotan T. Transcriptome profiling of the dynamic life cycle of the scypohozoan jellyfish Aurelia aurita. BMC Genomics. 2015;16:74. doi:10.1186/s12864-015-1320-z.
34. Ge J, Liu C, Tan J, Bian L and Chen S. Transcriptome analysis of scyphozoan jellyfish Rhopilema esculentum from polyp to medusa identifies potential genes regulating strobilation. Dev Genes Evol. 2018;228 6:243-54. doi:10.1007/s00427-018-0621-z.
35. Yamakawa S, Morino Y, Honda M and Wada H. The role of retinoic acid signaling in starfish metamorphosis. EvoDevo. 2018;9 1 doi:10.1186/s13227-018-0098-x.
36. Ryan JF, Mazza ME, Pang K, Matus DQ, Baxevanis AD, Martindale MQ, et al. Pre-bilaterian origins of the Hox cluster and the Hox code: evidence from the sea anemone, Nematostella vectensis. PLoS One. 2007;2 1:e153. doi:10.1371/journal.pone.0000153.
37. Ryan JF, Burton PM, Mazza ME, Kwong GK, Mullikin JC and Finnerty JR. The cnidarian-bilaterian ancestor possessed at least 56 homeoboxes: evidence from the starlet sea anemone, Nematostella vectensis. Genome Biol. 2006;7 7:R64. doi:10.1186/gb-2006-7-7-R64.
38. Chourrout D, Delsuc F, Chourrout P, Edvardsen RB, Rentzsch F, Renfer E, et al. Minimal ProtoHox cluster inferred from bilaterian and cnidarian Hox complements. Nature. 2006;442 7103:684-7. doi:10.1038/nature04863.
39. <PKS from Bacteria to Filamentous Ascomycota.pdf>.

40. Finnerty JR, Pang K, Burton P, Paulson D and Martindale MQ. Origins of bilateral symmetry: Hox and dpp expression in a sea anemone. Science. 2004;304 5675:1335-7. doi:10.1126/science.1091946.
41. He S, Del Viso F, Chen CY, Ikmi A, Kroesen AE and Gibson MC. An axial Hox code controls tissue segmentation and body patterning in Nematostella vectensis. Science. 2018;361 6409:1377-80. doi:10.1126/science.aar8384.
42. Schummer M, Scheurlen I, Schaller C and Galliot B. HOM/HOX homeobox genes are present in hydra (Chlorohydra viridissima) and are differentially expressed during regeneration. EMBO J. 1992;11 5:1815-23.
43. Gauchat D, Mazet F, Berney C, Schummer M, Kreger S, Pawlowski J, et al. Evolution of Antp-class genes and differential expression of Hydra Hox/paraHox genes in anterior patterning. Proc Natl Acad Sci U S A. 2000;97 9:4493-8.
44. Chiori R, Jager M, Denker E, Wincker P, Da Silva C, Le Guyader H, et al. Are Hox genes ancestrally involved in axial patterning? Evidence from the hydrozoan *Clytia hemisphaerica* (Cnidaria). PLoS One. 2009;4 1:e4231. doi:10.1371/journal.pone.0004231.
45. Shenk MA, Bode HB and Steele RE. Expression of Cnox-2, a HOM:HOX homeobox gene in hydra, is correlated with axial pattern formation. Development. 1993;117:657-67.
46. Reddy PC, Unni MK, Gungi A, Agarwal P and Galande S. Evolution of Hox-like genes in Cnidaria: Study of Hydra Hox repertoire reveals tailor-made Hox-code for Cnidarians. Mech Dev. 2015;138 Pt 2:87-96. doi:10.1016/j.mod.2015.08.005.
47. Kuhn K, Streit B and Schierwater B. Isolation of Hox Genes From the Scyphozoan *Cassiopeia xamachana*: Implications for the Early Evolution of Hox Genes. Journal of Experimental Zoology. 1999;285:63-75.
48. Kamm K and Schierwater B. Ancient linkage of a POU class 6 and an anterior Hox-like gene in cnidaria: implications for the evolution of homeobox genes. Journal of Experimental Zoology. 2007;308 6:777-84. doi:10.1002/jez.b.21196.
49. Chapman JA, Kirkness EF, Simakov O, Hampson SE, Mitros T, Weinmaier T, et al. The dynamic genome of Hydra. Nature. 2010;464 7288:592-6. doi:10.1038/nature08830.
50. Shinzato C, Mungpakdee S, Satoh N and Shoguchi E. A genomic approach to coral-dinoflagellate symbiosis: studies of Acropora digitifera and Symbiodinium minutum. Front Microbiol. 2014;5:336. doi:10.3389/fmicb.2014.00336.
51. Cunning R, Bay RA, Gillette P, Baker AC and Traylor-Knowles N. Comparative analysis of the Pocillopora damicornis genome highlights role of immune system in coral evolution. Sci Rep. 2018;8 1:16134. doi:10.1038/s41598-018-34459-8.
52. Jiang J, Quattrini AM, Francis WR, Ryan JF, Rodriguez E and McFadden CS. Hybrid de novo Assembly of the Sea Pansy (Renilla muelleri) Genome. bioRxiv. 2018; doi:10.1101/424614.
53. Ying H, Cooke I, Sprungala S, Wang W, Hayward DC, Tang Y, et al. Comparative genomics reveals the distinct evolutionary trajectories of the robust and complex coral lineages. Genome Biol. 2018;19 1:175. doi:10.1186/s13059-018-1552-8.
54. Voolstra CR, Li Y, Liew YJ, Baumgarten S, Zoccola D, Flot JF, et al. Comparative analysis of the genomes of Stylophora pistillata and Acropora digitifera provides evidence for extensive differences between species of corals. Sci Rep. 2017;7 1:17583. doi:10.1038/s41598-017-17484-x.

55. Leclère L, Horin C, Chevalier S, Lapébie P, Dru P, Peron S, et al. The genome of the jellyfish Clytia hemisphaerica and the evolution of the cnidarian life-cycle. bioRxiv. 2018:1-41. doi:10.1101/369959.

56. Putnam NH, Srivastava M, Hellsten U, Dirks B, Chapman J, Salamov A, et al. Sea anemone genome reveals ancestral eumetazoan gene repertoire and genomic organization. Science. 2007;317 5834:86-94. doi:10.1126/science.1139158.

57. Leclère L. The genome of the jellyfish Clytia hemisphaerica and the evolution of the cnidarian life-cycle. Nature Ecology & Evolution. 2019; doi:10.1038/s41559-019-0833-2.

58. Gold DA, Katsuki T, Li Y, Yan X, Regulski M, Ibberson D, et al. The genome of the jellyfish Aurelia and the evolution of animal complexity. Nature Ecology & Evolution. 2018; doi:10.1038/s41559-018-0719-8.

59. Nastav B, Malej M, Malej Jr A and Malej A. Is it possible to determine the economic impact of jellyfish outbreaks on fisheries? A Case Study – Slovenia. Mediterranean Marine Science. 2013;14 1:214. doi:10.12681/mms.382.

60. Purcell JE and Arai MN. Interactions of pelagic cnidarians and ctenophores with fish: a review. Hydrobiologia. 2001;451 1-3:27-44. doi:Doi 10.1023/A:1011883905394.

61. Purcell JE, Uye S and Lo W. Anthropogenic causes of jellyfish blooms and their direct consequences for humans: a review. Marine Ecology Progress Series. 2007;350:153-74. doi:10.3354/meps07093.

62. Lawley JW, Ames CL, Bentlage B, Yanagihara A, Goodwill R, Kayal E, et al. Box Jellyfish Alatina alata Has a Circumtropical Distribution. Biol Bull. 2016;231 2:152-69. doi:10.1086/690095.

63. Garcia-Rodriguez J, Lewis Ames C, Marian J and Marques AC. Gonadal histology of box jellyfish (Cnidaria: Cubozoa) reveals variation between internal fertilizing species *Alatina alata* (Alatinidae) and *Copula sivickisi* (Tripedaliidae). J Morphol. 2018;279 6:841-56. doi:10.1002/jmor.20815.

64. Corw GL, Chiaverano LM, Crites J, Khramov MA and Holland BS. Box Jellyfish (Cubozoa: Carybdeida) in Hawaiian Waters, and the First Record of Tripedalia cystophora in Hawaiʻi. Bishop Museum Bulletin in Zoology. 2015;9:93-108.

65. Lampert KP. Cassiopea and Its Zooxanthellae. In: Goffredo S and Dubinsky Z, editors. The Cnidaria, Past, Present and Future: The world of Medusa and her sisters. Switzerland: Springer International Publishing; 2016. p. 415-23.

66. Ohdera AH, Abrams MJ, Ames CL, Baker DM, Suescún-Bolívar LP, Collins AG, et al. Upside-Down but Headed in the Right Direction: Review of the Highly Versatile Cassiopea xamachana System. Frontiers in Ecology and Evolution. 2018;6 doi:10.3389/fevo.2018.00035.

67. Bolger AM, Lohse M and Usadel B. Trimmomatic: a flexible trimmer for Illumina sequence data. Bioinformatics. 2014;30 15:2114-20. doi:10.1093/bioinformatics/btu170.

68. Gnerre S, MacCallum I, Przybylski D, Ribeiro FJ, Burton JN, Walker BJ, et al. High-quality draft assemblies of mammalian genomes from massively parallel sequence data. Proceedings of the National Academy of Sciences. 2011;108 4:1513-8. doi:10.1073/pnas.1017351108.

69. Simpson JT, Wong K, Jackman SD, Schein JE, Jones SJ and Birol I. ABySS: a parallel assembler for short read sequence data. Genome Res. 2009;19 6:1117-23. doi:10.1101/gr.089532.108.

70. Bankevich A, Nurk S, Antipov D, Gurevich AA, Dvorkin M, Kulikov AS, et al. SPAdes: a new genome assembly algorithm and its applications to single-cell sequencing. J Comput Biol. 2012;19 5:455-77. doi:10.1089/cmb.2012.0021.
71. Kajitani R, Toshimoto K, Noguchi H, Toyoda A, Ogura Y, Okuno M, et al. Efficient de novo assembly of highly heterozygous genomes from whole-genome shotgun short reads. Genome Res. 2014;24 8:1384-95. doi:10.1101/gr.170720.113.
72. Parra G, Bradnam K and Korf I. CEGMA: a pipeline to accurately annotate core genes in eukaryotic genomes. Bioinformatics. 2007;23 9:1061-7. doi:10.1093/bioinformatics/btm071.
73. Simao FA, Waterhouse RM, Ioannidis P, Kriventseva EV and Zdobnov EM. BUSCO: assessing genome assembly and annotation completeness with single-copy orthologs. Bioinformatics. 2015;31 19:3210-2. doi:10.1093/bioinformatics/btv351.
74. Nishimura O, Hara Y and Kuraku S. gVolante for standardizing completeness assessment of genome and transcriptome assemblies. Bioinformatics. 2017;33 22:3635-7. doi:10.1093/bioinformatics/btx445.
75. Kayal E, Bentlage B, Cartwright P, Yanagihara AA, Lindsay DJ, Hopcroft RR, et al. Phylogenetic analysis of higher-level relationships within Hydroidolina (Cnidaria: Hydrozoa) using mitochondrial genome data and insight into their mitochondrial transcription. PeerJ. 2015;3:e1403. doi:10.7717/peerj.1403.
76. Lowe TM and Eddy SR. tRNAscan-SE: A program for improved detection of transfer RNA genes in genomic sequence. Nucleic Acids Research. 1997;25 5:955-64. doi:DOI 10.1093/nar/25.5.955.
77. Laslett D and Canback B. ARWEN: a program to detect tRNA genes in metazoan mitochondrial nucleotide sequences. Bioinformatics. 2008;24 2:172-5. doi:10.1093/bioinformatics/btm573.
78. Boetzer M, Henkel CV, Jansen HJ, Butler D and Pirovano W. Scaffolding pre-assembled contigs using SSPACE. Bioinformatics. 2011;27 4:578-9. doi:10.1093/bioinformatics/btq683.
79. Zimin AV, Marcais G, Puiu D, Roberts M, Salzberg SL and Yorke JA. The MaSuRCA genome assembler. Bioinformatics. 2013;29 21:2669-77. doi:10.1093/bioinformatics/btt476.
80. Vinogradov AE. Intron-genome size relationship on a large evolutionary scale. J Mol Evol. 1999;49 3:376-84.
81. Grau JH, Hackl T, Koepfli KP and Hofreiter M. Improving draft genome contiguity with reference-derived in silico mate-pair libraries. Gigascience. 2018;7 5 doi:10.1093/gigascience/giy029.
82. Miller DE, Staber C, Zeitlinger J and Hawley RS. Highly Contiguous Genome Assemblies of 15 Drosophila Species Generated Using Nanopore Sequencing. G3 (Bethesda). 2018;8 10:3131-41. doi:10.1534/g3.118.200160.
83. Stanke M and Waack S. Gene prediction with a hidden Markov model and a new intron submodel. Bioinformatics. 2003;19 Suppl 2:ii215-ii25. doi:10.1093/bioinformatics/btg1080.
84. Kent WJ. BLAT -- The BLASTlike Alignment Tool. Genome Research. 2002;12:656-64.
85. Emms DM and Kelly S. OrthoFinder: solving fundamental biases in whole genome comparisons dramatically improves orthogroup inference accuracy. Genome Biol. 2015;16:157. doi:10.1186/s13059-015-0721-2.

86. Grabherr MG, Haas BJ, Yassour M, Levin JZ, Thompson DA, Amit I, et al. Full-length transcriptome assembly from RNA-Seq data without a reference genome. Nat Biotechnol. 2011;29 7:644-52. doi:10.1038/nbt.1883.
87. Haas BJ, Papanicolaou A, Yassour M, Grabherr M, Blood PD, Bowden J, et al. De novo transcript sequence reconstruction from RNA-Seq: reference generation and analysis with Trinity. Nature protocols. 2013;8 8:10.1038/nprot.2013.084. doi:10.1038/nprot.2013.084.
88. Boutet E, Lieberherr D, Tognolli M, Schneider M and Bairoch A. UniProtKB/Swiss-Prot. In: Edwards D, editor. Plant Bioinformatics: Methods and Protocols. Totowa, NJ: Humana Press; 2007. p. 89-112.
89. Yu G, Wang LG, Han Y and He QY. clusterProfiler: an R package for comparing biological themes among gene clusters. OMICS. 2012;16 5:284-7. doi:10.1089/omi.2011.0118.
90. Carlson M and Pagès H. AnnotationForge: Code for Building Annotation Database Packages. R package version 1240. 2018; doi:10.18129/B9.bioc.AnnotationForge.
91. Macrander J, Panda J, Janies D, Daly M and Reitzel AM. Venomix: a simple bioinformatic pipeline for identifying and characterizing toxin gene candidates from transcriptomic data. PeerJ. 2018;6:e5361. doi:10.7717/peerj.5361.
92. Camacho C, Coulouris G, Avagyan V, Ma N, Papadopoulos J, Bealer K, et al. BLAST+: architecture and applications. BMC Bioinformatics. 2009;10:421. doi:10.1186/1471-2105-10-421.
93. Sher D, Fishman Y, Zhang M, Lebendiker M, Gaathon A, Mancheno JM, et al. Hydralysins, a new category of beta-pore-forming toxins in cnidaria. J Biol Chem. 2005;280 24:22847-55. doi:10.1074/jbc.M503242200.
94. Oliveira JS, Fuentes-Silva D and King GF. Development of a rational nomenclature for naming peptide and protein toxins from sea anemones. Toxicon. 2012;60 4:539-50. doi:10.1016/j.toxicon.2012.05.020.
95. Nagai H, Takuwa-Kuroda K, Nakao M, Oshiro N, Iwanaga S and Nakajima T. A novel protein toxin from the deadly box jellyfish (Sea Wasp, Habu-kurage) Chiropsalmus quadrigatus. Bioscience, biotechnology, and biochemistry. 2002;66 1:97-102. doi:10.1271/bbb.66.97.
96. Nagai H, Takuwa K, Nakao M, Ito E, Miyake M, Noda M, et al. Novel Proteinaceous Toxins from the Box Jellyfish (Sea Wasp) Carybdea rastoni. Biochemical and Biophysical Research Communications. 2000;275 2:582-8. doi:https://doi.org/10.1006/bbrc.2000.3353.
97. Nagai H, Takuwa K, Nakao M, Sakamoto B, Crow GL and Nakajima T. Isolation and characterization of a novel protein toxin from the Hawaiian box jellyfish (sea wasp) Carybdea alata. Biochemical and Biophysical Research Communications. 2000;275 2:589-94. doi:10.1006/bbrc.2000.3352.
98. Pedrera L, Fanani ML, Ros U, Lanio ME, Maggio B and Alvarez C. Sticholysin I-membrane interaction: an interplay between the presence of sphingomyelin and membrane fluidity. Biochim Biophys Acta. 2014;1838 7:1752-9. doi:10.1016/j.bbamem.2014.03.011.
99. Rogers AD. Cnidarians (Cnidaria). In: Hedges SB and Kumar S, editors. The Timetree of Life. Oxford University Press, USA; 2009. p. 233-8.
100. Shinzato C, Shoguchi E, Kawashima T, Hamada M, Hisata K, Tanaka M, et al. Using the Acropora digitifera genome to understand coral responses to environmental change. Nature. 2011;476 7360:320-3. doi:10.1038/nature10249.

101. Simakov O, Marletaz F, Cho SJ, Edsinger-Gonzales E, Havlak P, Hellsten U, et al. Insights into bilaterian evolution from three spiralian genomes. Nature. 2013;493 7433:526-31. doi:10.1038/nature11696.
102. Chipman AD, Ferrier DE, Brena C, Qu J, Hughes DS, Schroder R, et al. The first myriapod genome sequence reveals conservative arthropod gene content and genome organisation in the centipede Strigamia maritima. PLoS Biol. 2014;12 11:e1002005. doi:10.1371/journal.pbio.1002005.
103. Albertin CB, Simakov O, Mitros T, Wang ZY, Pungor JR, Edsinger-Gonzales E, et al. The octopus genome and the evolution of cephalopod neural and morphological novelties. Nature. 2015;524 7564:220-4. doi:10.1038/nature14668.
104. Wang S, Zhang J, Jiao W, Li J, Xun X, Sun Y, et al. Scallop genome provides insights into evolution of bilaterian karyotype and development. Nat Ecol Evol. 2017;1 5:120. doi:10.1038/s41559-017-0120.
105. Dehal P, Satou Y, Campbell RK, Chapman J, Degnan B, De Tomaso A, et al. The Draft Genome of Ciona intestinalis: Insights into Chordate and Vertebrate Origins. Science. 2002;298 5601:2157.

Figure 1. A) *Calvadosia cruxmelitensis* (Staurozoa), B) *Alatina alata* (Cubozoa), and C) *Cassiopea xamachana* (Scyphozoa). D) Phylogenetic relationship of major cnidarian lineages after Kayal et al. (2018), revealing Cubozoa and Scyphozoa as sister groups, united with Staurozoa to form Acraspeda.

Figure 2*. Gene Content Distribution in Cnidarian Lineages.* Filled circles in the bottom panel indicate shared orthogroups in those lineages. Bar graphs indicate the number of orthogroups corresponding to each filled-circle pattern. Numbers next to each species abbreviation indicate the total number of orthogroups identified for that species. Hsap = *Homo sapiens*; Nvec = *Nematostella vectensis*; Hmag = *Hydra magnipapillata*; Ccrux = *Calvadosia cruxmelitensis*; Aala = *Alatina alata*; Cxam = *Cassiopea xamachana*.

Figure 3. *Gene ontology biological processes over-enriched within Cnidaria specific orthogroups visualized using ReViGO*. Over-representation analysis was performed with ClusterProfiler, with a p-adjusted cutoff of 0.01. Color indicates Log10 transformed p-adjusted value. Terms are plotted within an x-y semantic space, in which similar terms are clustered within closer proximities. Color indicates p-value and circle size indicates frequency of GO term in the *Cassiopea* database.

Figure 4. *Gene ontology biological processes over-enriched within Medusozoa specific orthogroups visualized using ReViGO*. Over-representation analysis was performed with ClusterProfiler, with a p-adjusted cutoff of 0.01. Color indicates Log10 transformed p-adjusted value. Terms are plotted within an x-y semantic space, in which similar terms are clustered within closer proximities. Color indicates p-value and circle size indicates frequency of GO term in the *Cassiopea* database.

Figure 5. *Gene ontology biological processes over-enriched within Acraspeda specific orthogroups visualized using ReViGO*. Over-representation analysis was performed with ClusterProfiler, with a p-adjusted cutoff of 0.01. Color indicates Log10 transformed p-adjusted value. Terms are plotted within an x-y semantic space, in which similar terms are clustered within closer proximities. Color indicates p-value and circle size indicates frequency of GO term in the *Cassiopea* database.

Figure 6. : *Venom-encoding gene repertoire of five cnidarian genomes.* Venom-encoding genes were identified with the venomix database using OrthoFinder and BLAST. Numbers in parentheses indicate total venom types screened within each venom-associated family. A = *Calvadosia cruxmelitensis*, B = *Alatina alata*, C = *Cassiopea xamachana*, D = *Hydra magnipapillata*, and E = *Nematostella vectensis*

Figure 7. *Distribution of venom-related genes in cnidarian lineages*. Filled circles in the bottom panel indicate presence of shared venom-related genes in those lineages. Bar graphs indicate the number of venom-related orthogroups corresponding to each filled-circle pattern. Numbers next to each species abbreviation indicate the total number of venom-related orthogroups identified for that species. Hsap = *Homo sapiens*; Nvec = *Nematostella vectensis*; Hmag = *Hydra magnipapillata*; Ccrux = *Calvadosia cruxmelitensis*; Aala = *Alatina alata*; Cxam = *Cassiopea xamachana*.

Figure 8. *Evolution of PPCS/POU gene fusion and POU-Hox linkage*. A fusion event involving a POU and PPCS domain occurred in the stem of Cnidaria. The syntenic linkage of POU and Hox genes occurred at least twice in animal evolution: once in the stem of Medusozoa and once in the vertebrate lineage.

	Alatina alata	*Calvadosia cruxmelitensis*	*Cassiopea xamachana*
NCBI Taxa ID	1193083	1843192	12993
# of Sequences	291,445	50,999	93,483
Estimated Genome Size	2,673,604,203	230,957,924	361,689,769
Total Length (bp)	851,121,747	209,392,379	393,520,168
N50 (bp)	7,049	16,443	15,563
CEGMA (%Complete)	8.06	61.29	53.63
CEGMA (%Complete + Partial)	29.84	91.94	82.66
BUSCO (%Complete)	18.30	70.86	58.59
BUSCO (%Complete + Partial)	32.11	85.07	66.97
GC Content (%)	38.07	39.95	37.07
Assembly Accession	PUGI00000000	OFHS01000000	OLMO01000000
NCBI Raw Read Accession	PRJNA421156	PRJEB23739	PRJEB23739
Specimen Voucher ID	USNM 1248604	USNM 1286381	UF Cnidaria 12979

Table 1. Statistics of Alatina alata, Calvadosia cruxmelitensis, and Cassiopea xamachana genome assemblies**.**

Supplementary Material

Supplementary Figure S1. ReViGO output for Cnidaria genes clustered through k-means clustering by Euclidean distance. Number of optimal clusters predicted prior to clustering using NbClust.

Supplementary Figure S2. ReViGO output for Medusozoa genes clustered through k-means clustering by Euclidean distance. Number of optimal clusters predicted prior to clustering using NbClust.

*Supplementary Figure S3. Linkage of PPCS-POU Genes with Hox Genes in cnidarian Genomes. Genomic scaffolds for three Medusozoa lineages (*E. dichotoma, C cruxmelitensis, and Cassiopea xamachana*) show linkage of the PPCS-POU gene linked to a Hox gene (dark green). This linkage is not seen in Anthozoa (*N. vectensis*). The light green region indicates the transcribed portion of the scaffold, and exons are represented within by curved rectangles (PPCS exons = purple, POU exons = yellow). Scaffold length shown to the right of each bar. Edic =* Eleutheria dichotoma*; Ccrux = Calvadosia* cruxmelitensis*; Cxam =* Cassiopea xamachana*; Nvec =* Nematostella vectensis*.*

Table S1. Orthogroups specific to Cnidaria identified using OrthoFinder and annotated by a representative gene from the *Hydra magnipapillata* genome. Protein annotations were retrieved from Swiss-Prot.

Table S2. Orthogroups specific to Medusozoa identified using OrthoFinder and annotated by a representative gene from the *Hydra magnipapillata* genome. Protein annotations were retrieved from Swiss-Prot.

Table S3. Orthogroups specific to Acraspeda identified using OrthoFinder and annotated by a representative gene from the *Hydra magnipapillata* genome. Protein annotations were retrieved from Swiss-Prot.

Table S4: Venom-encoding gene repertoire of five cnidarian genomes identified via the venomix database and pipeline. Venom genes are categories by families (column 1). Both genomic and transcriptomic data were used, with transcriptomic isoforms counted as a single venom-encoding gene.

Ccrux_Figure1A

Click here to access/download;Figure;luc_crux_17-09-12_1.jpg

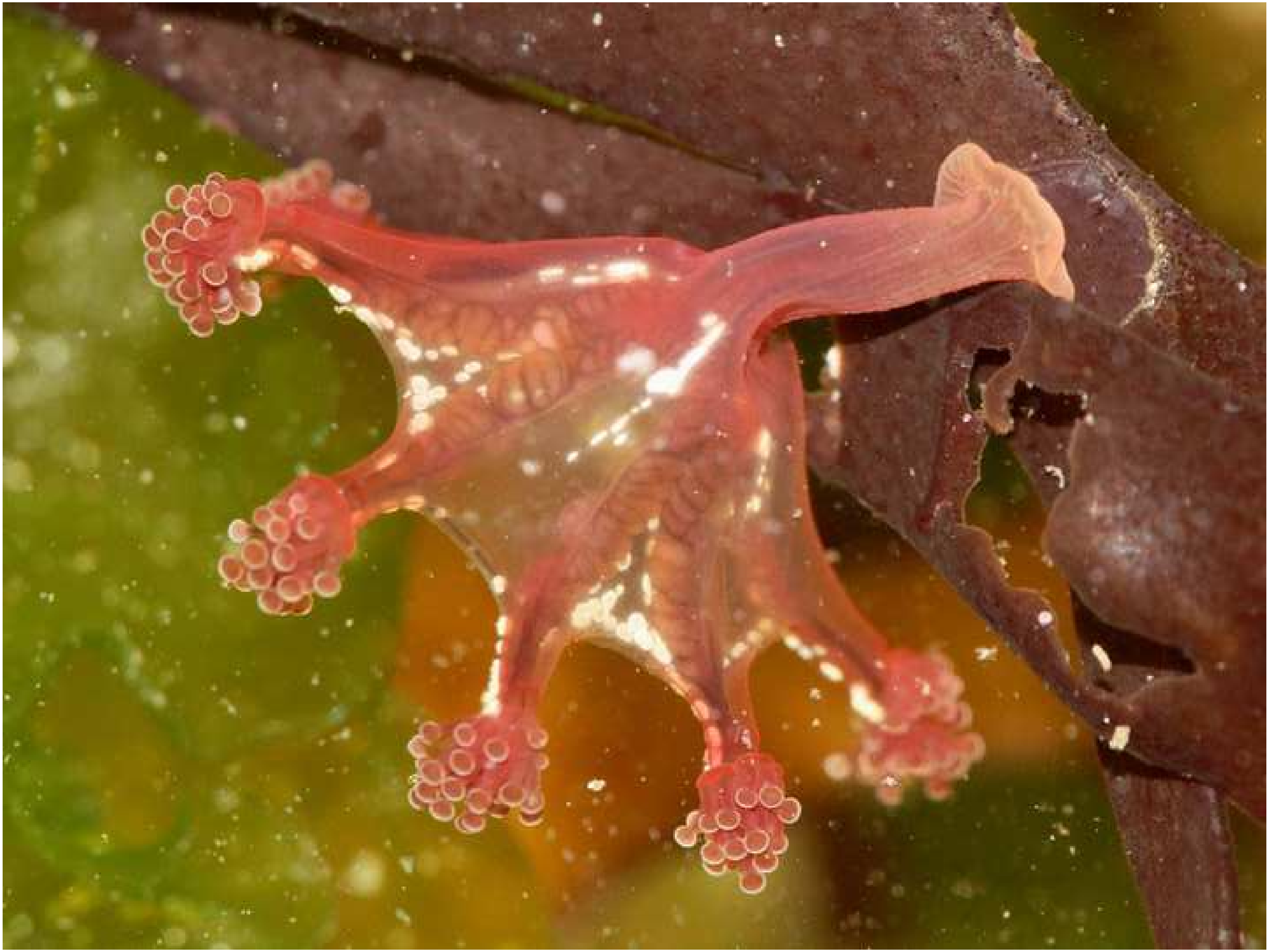

Aala_Figure1B

Click here to access/download;Figure;Alatina_alata_AGC_8965_on_black.jpg

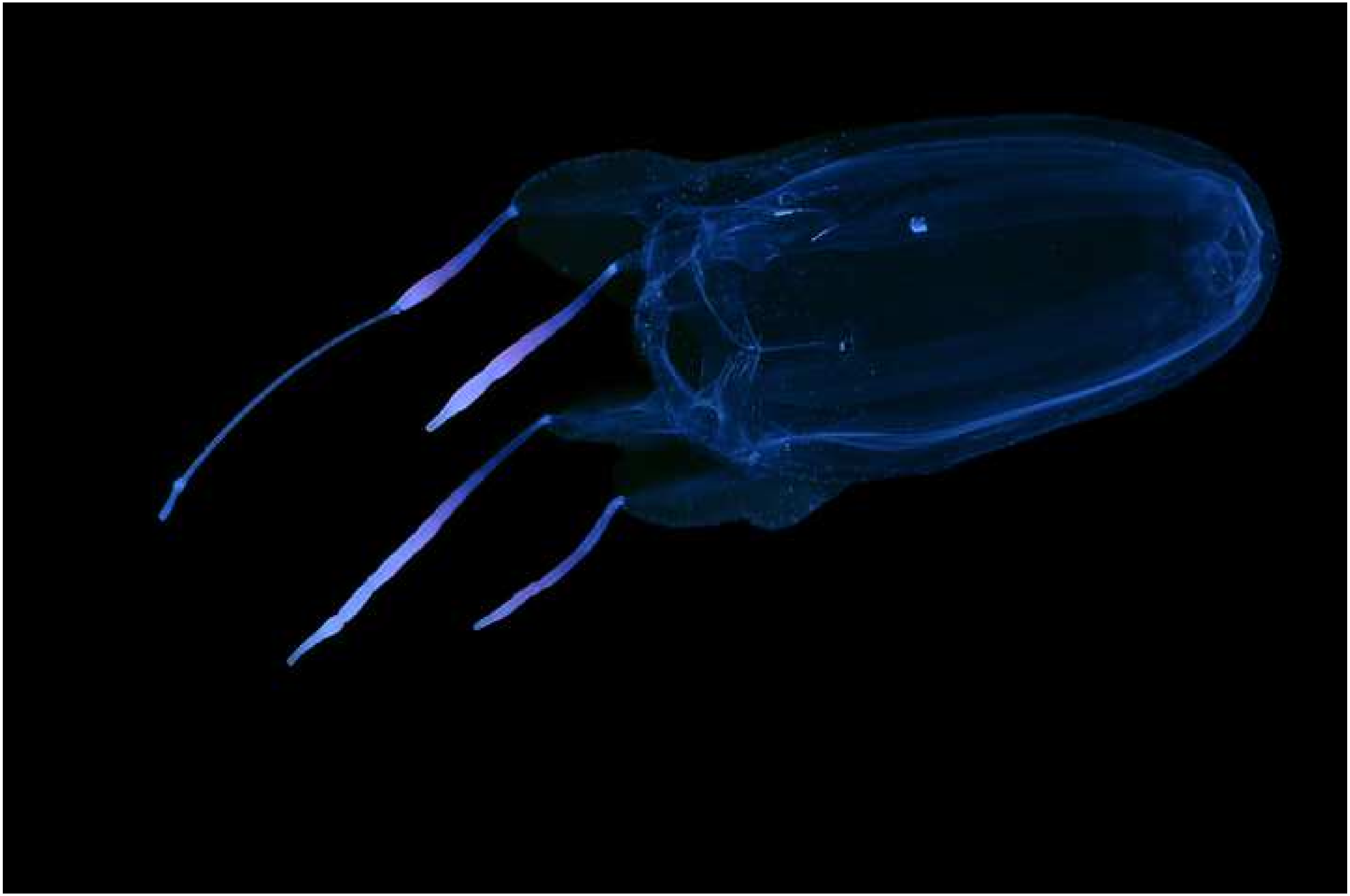

Cxam_Figure1C

Click here to access/download;Figure;Cassiopea.jpg

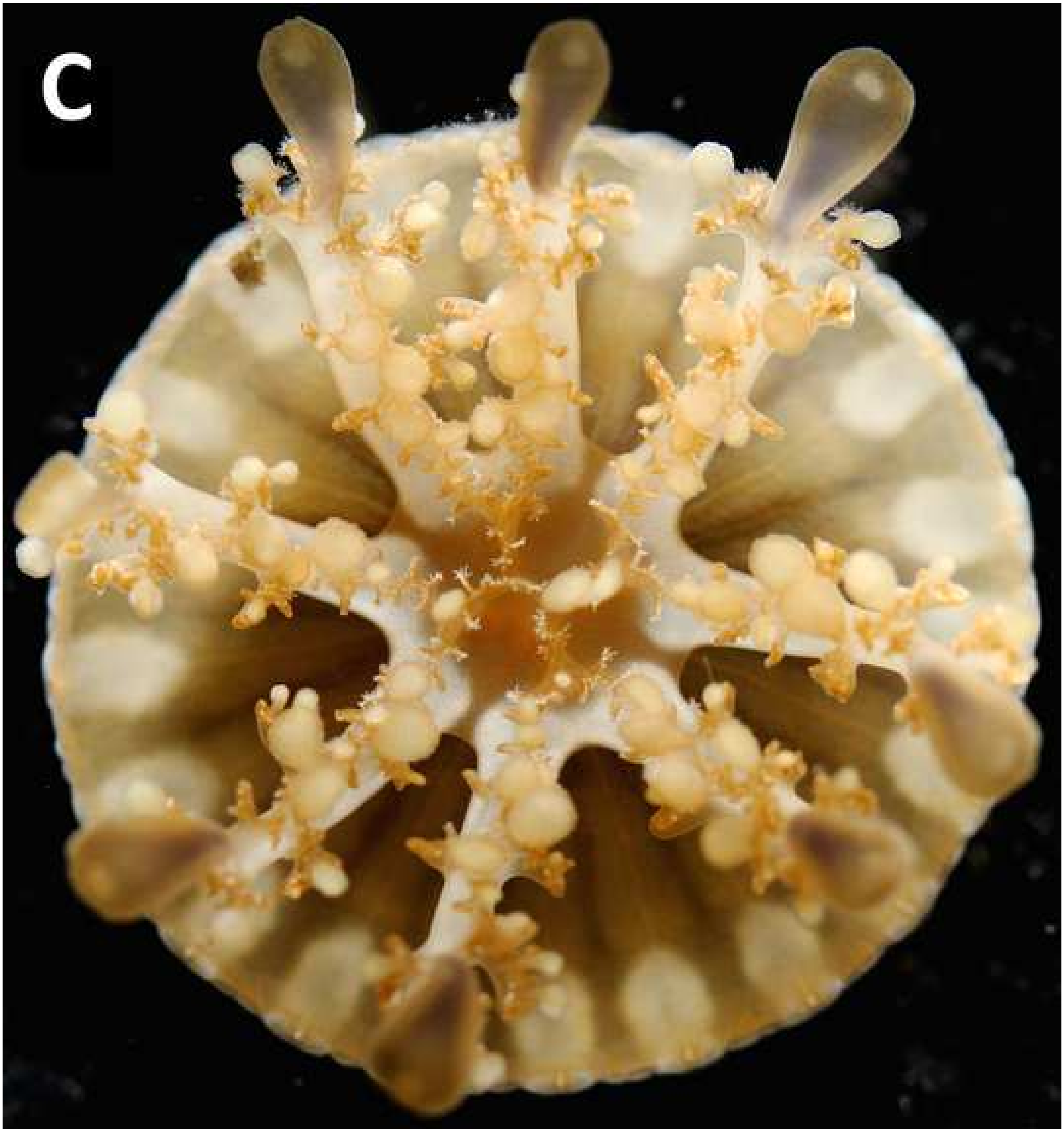

Figure4

Click here to access/download;Figure;Figure4.pdf

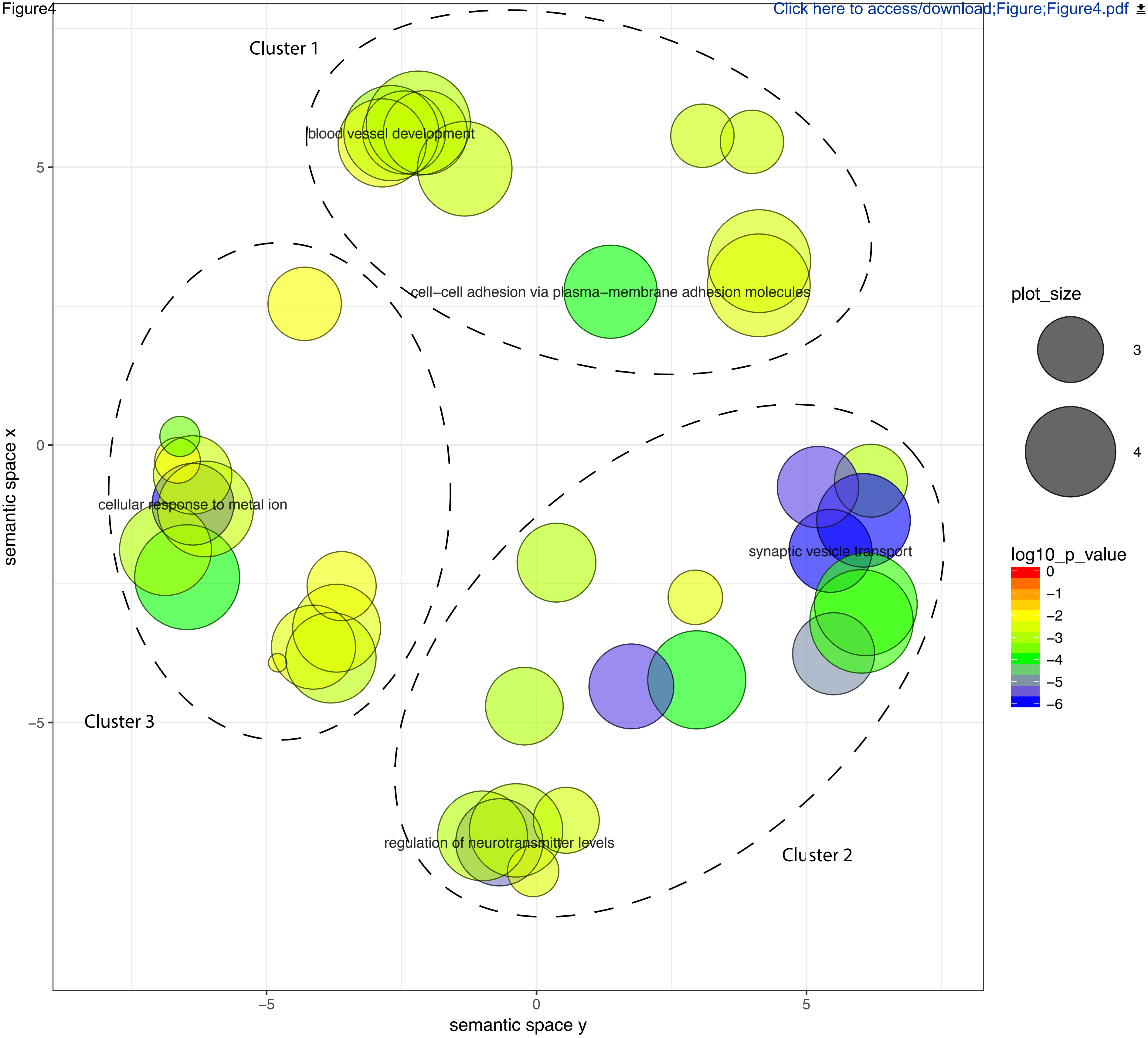

Figure5

Click here to access/download;Figure;Figure5.pdf

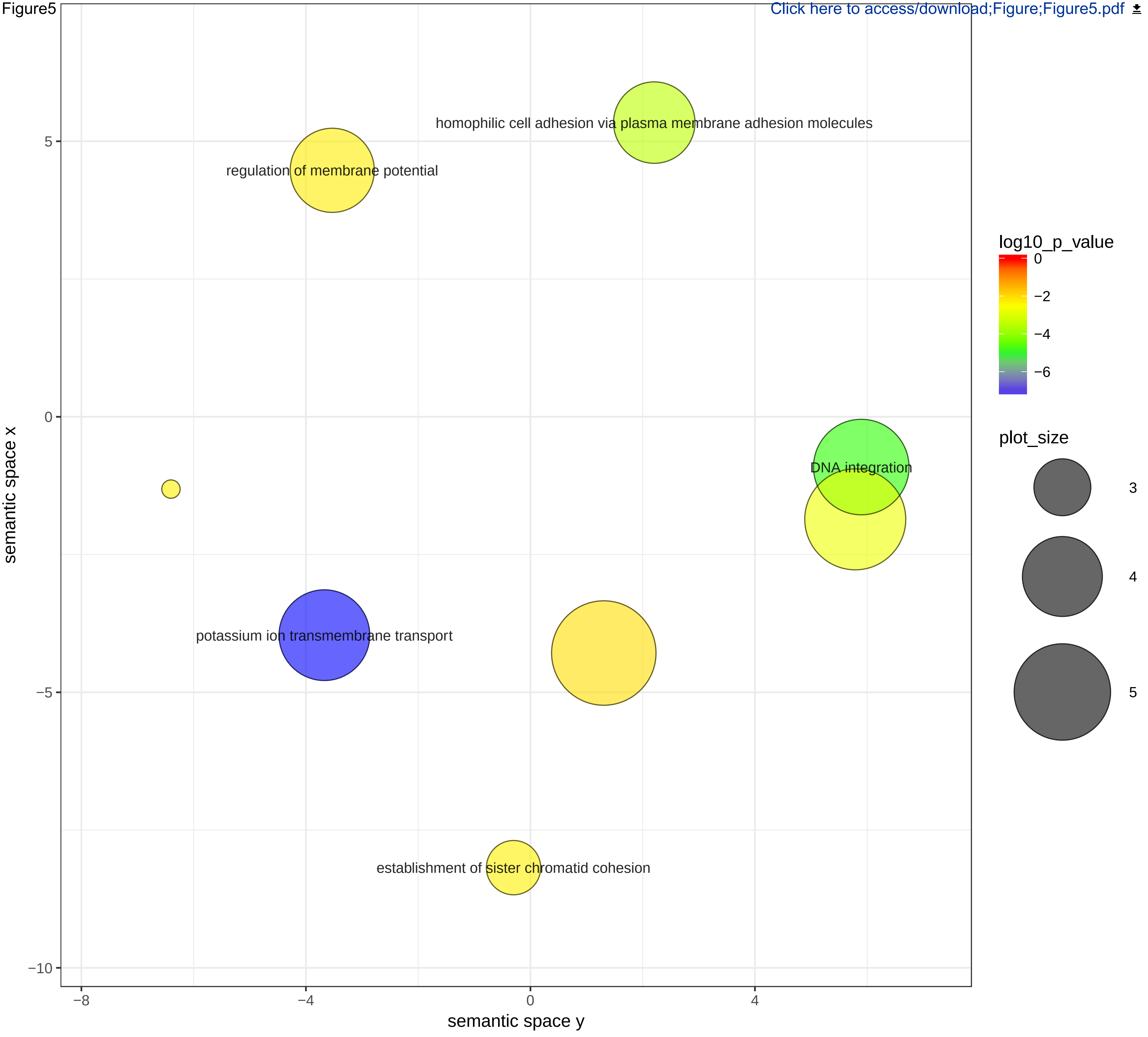

Figure6

Click here to access/download;Figure;Figure6.pdf

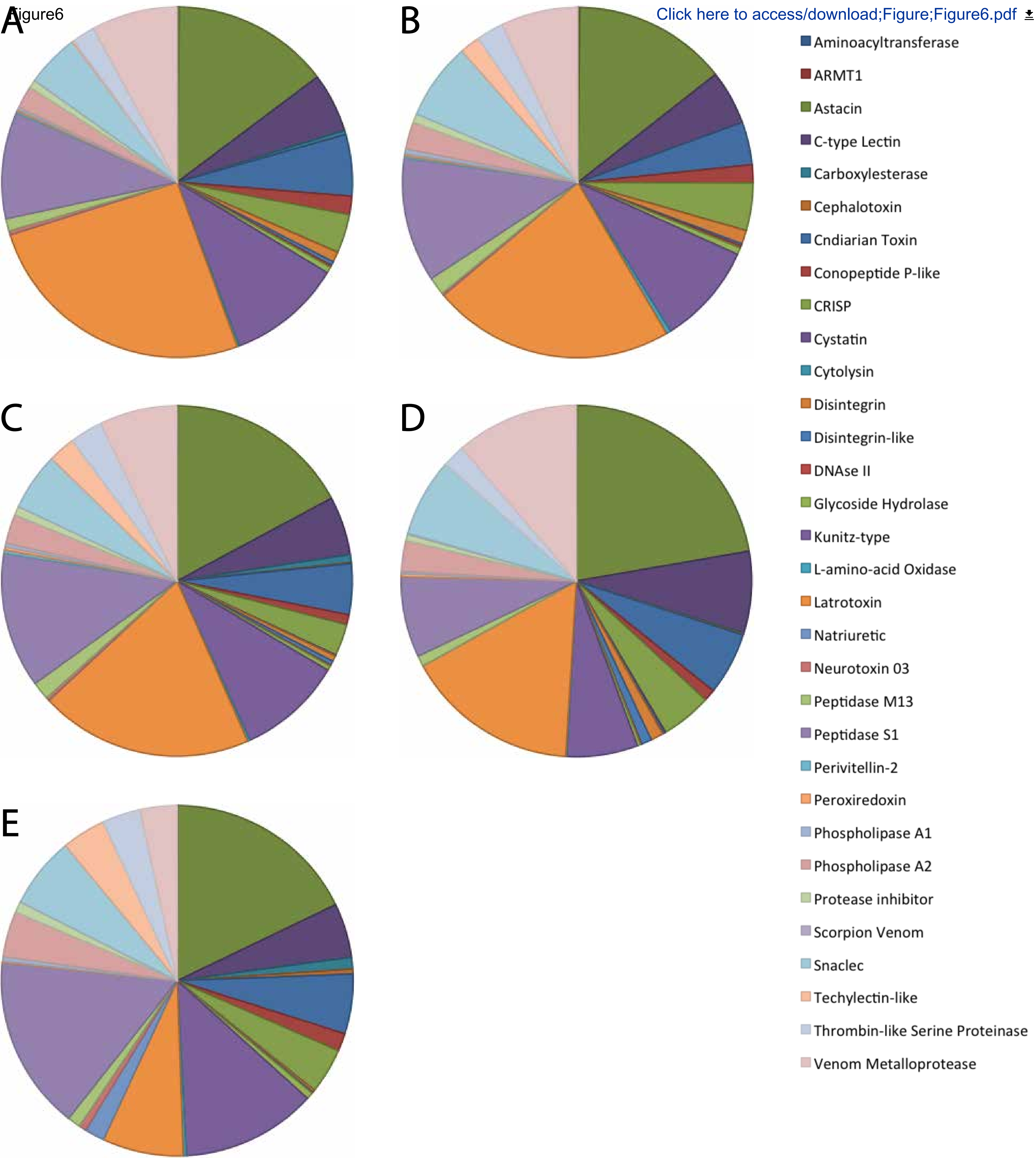

Click here to access/download;Figure;Figure7.pdf

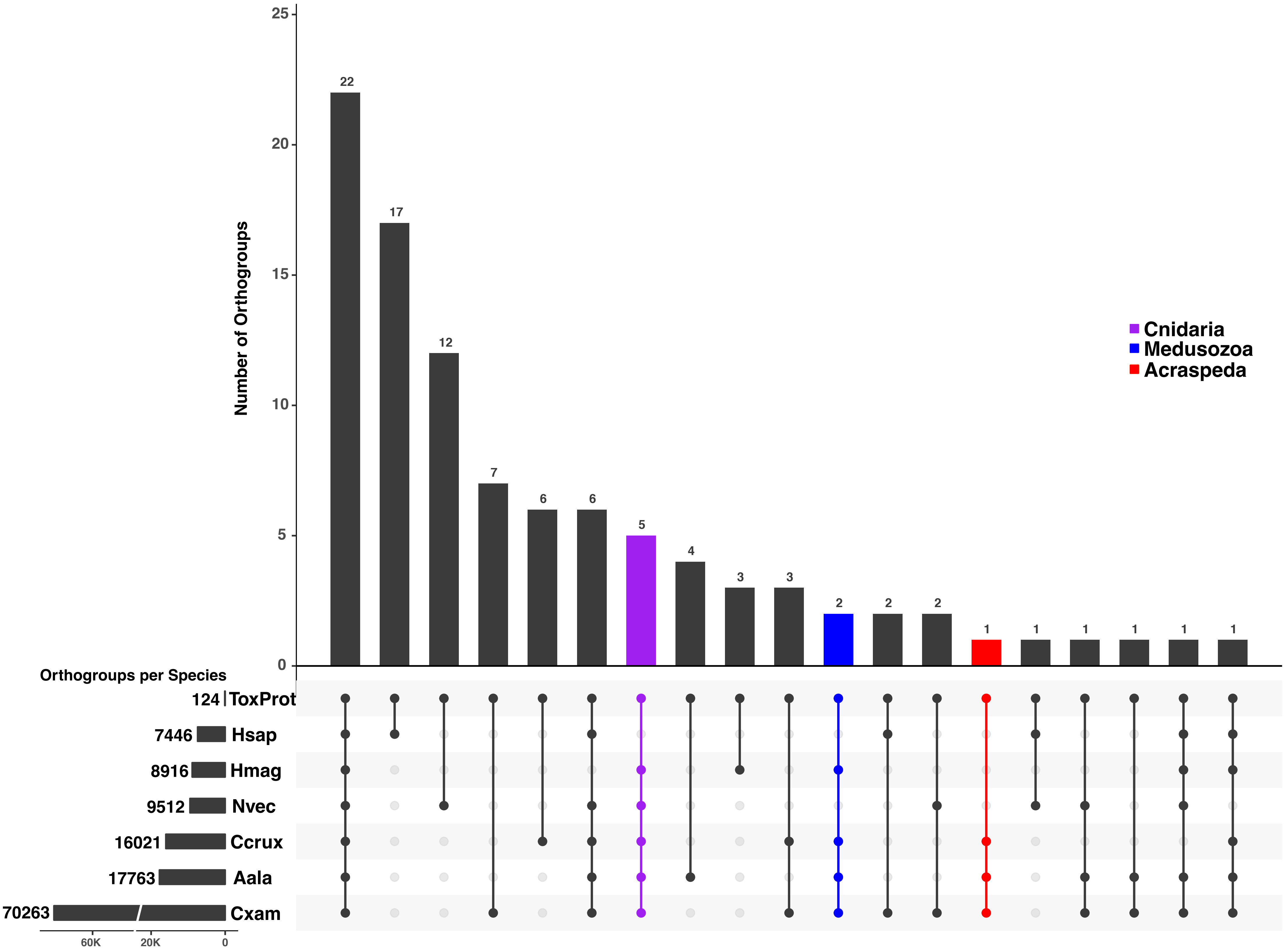

Figure8

Click here to access/download;Figure;Figure8.png

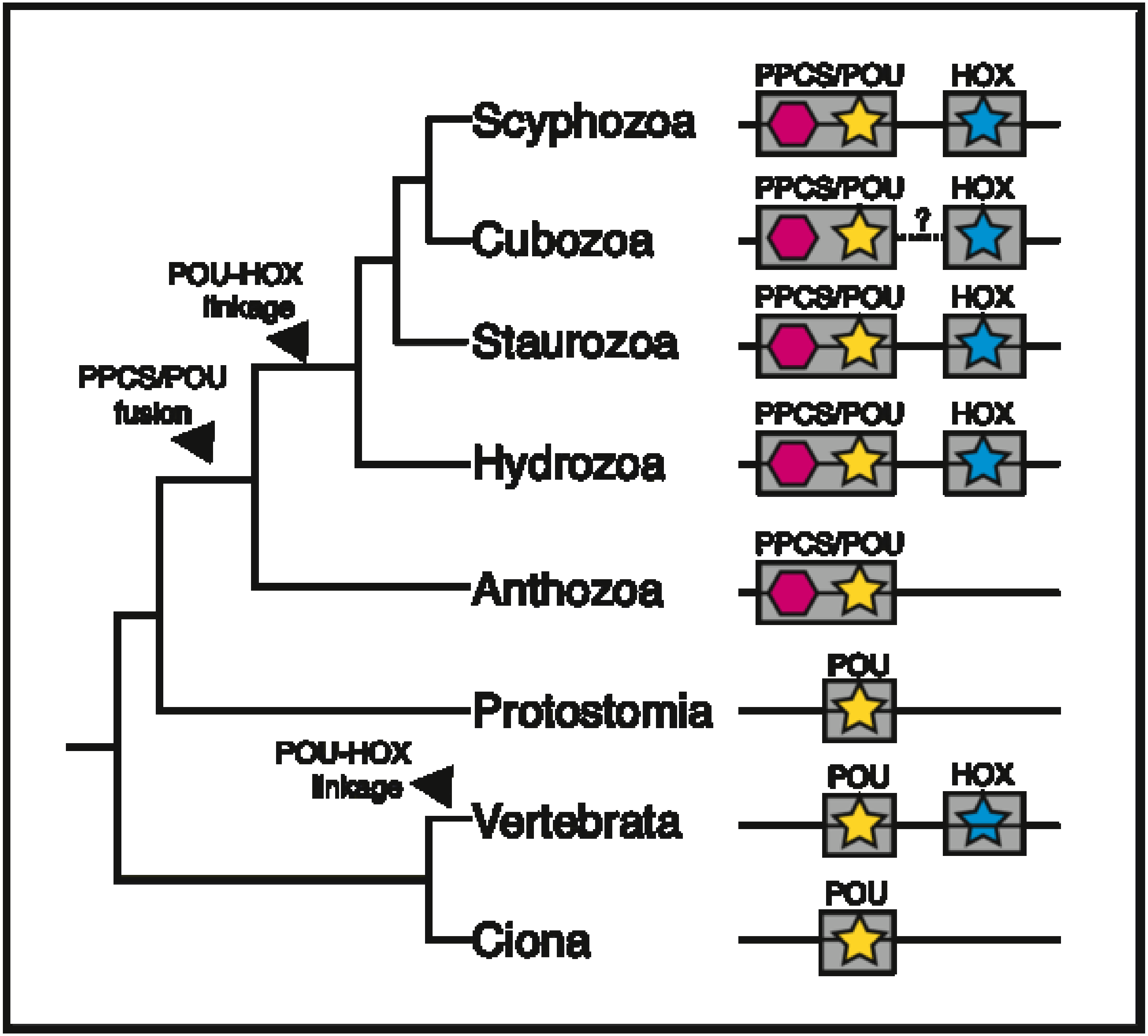

Figure2

Click here to access/download;Figure;Figure2.pdf

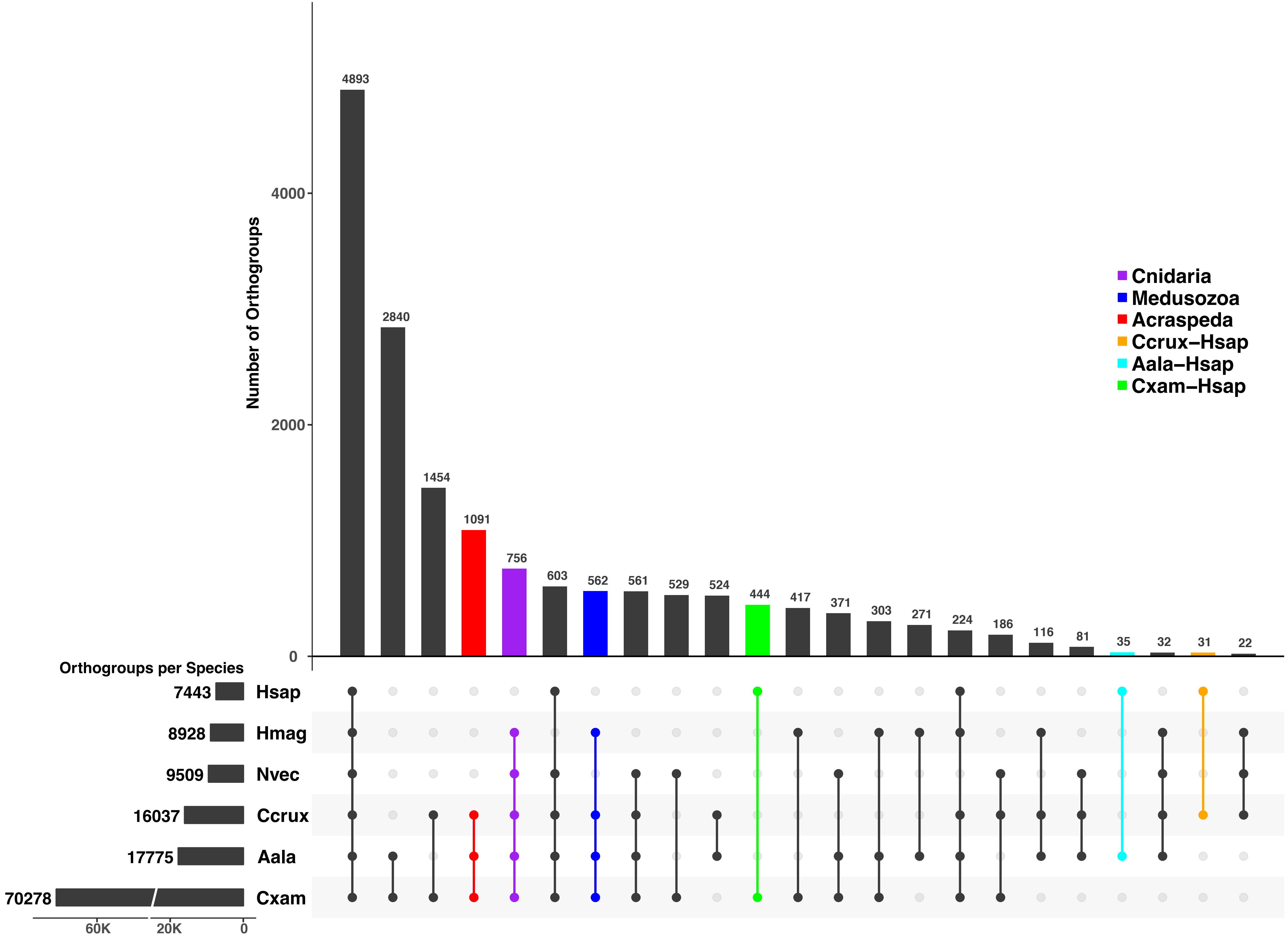

Figure3

Click here to access/download;Figure;Figure3.pdf

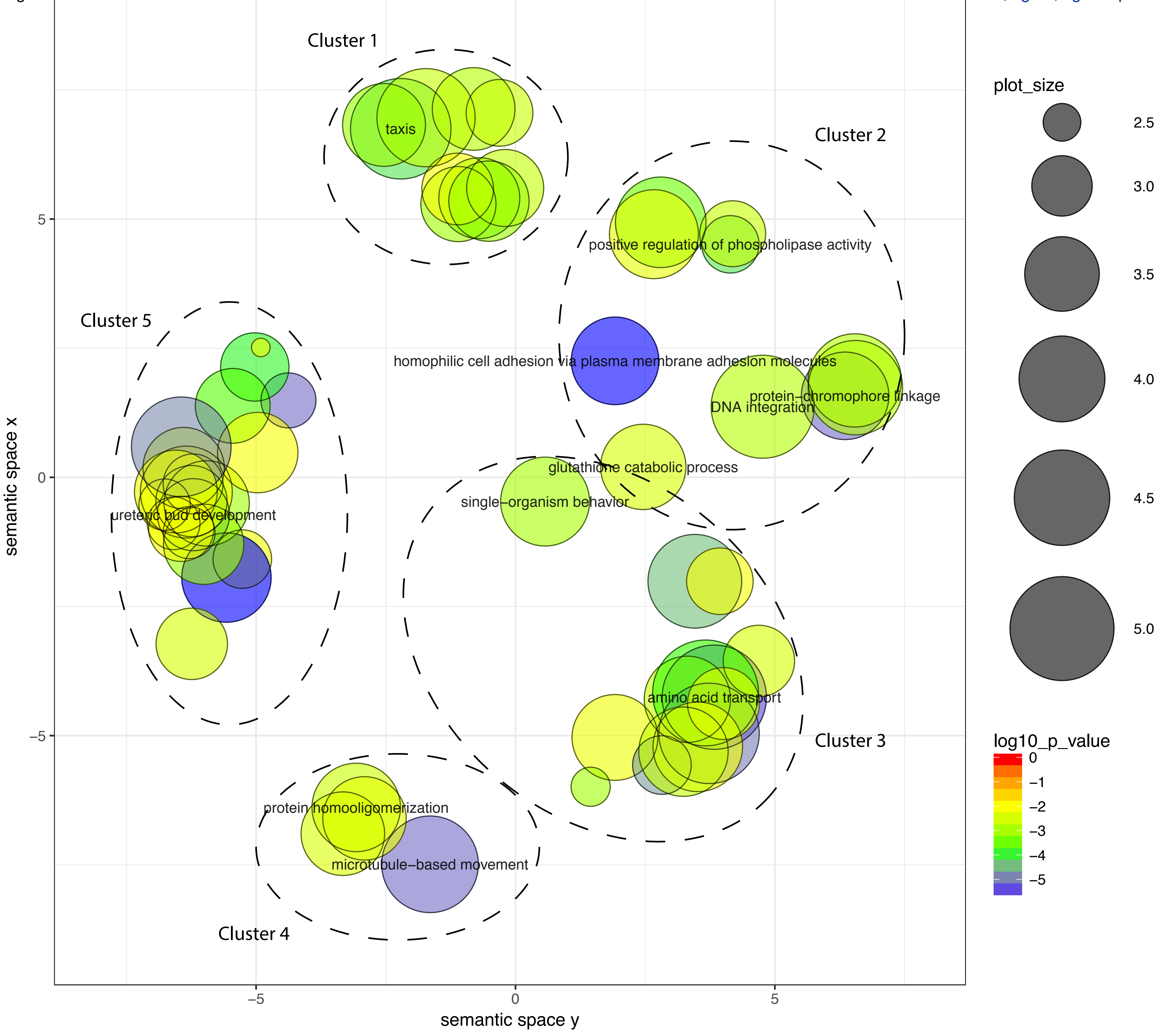

Supplementary Material - Figure S3

Click here to access/download
Supplementary Material
Figure4.jpg

Supplementary Material - Table S1

Click here to access/download
Supplementary Material
Table_S1.xlsx

Click here to access/download
Supplementary Material
Table_S2.xlsx

Supplementary Material - Table S3

Click here to access/download
Supplementary Material
Table_S3.xlsx

Click here to access/download
Supplementary Material
Table_S4.xlsx

Supplementary Material - Figure S1

Click here to access/download
Supplementary Material
Figure_S1.v2.pdf

Click here to access/download
Supplementary Material
Figure_S2.v2.pdf
