# Supplementary figures and images for: Box, stalked, and upside-down? Draft genomes from diverse jellyfish (Cnidaria, Acraspeda) lineages: Alatina alata (Cubozoa), Calvadosia cruxmelitensis (Staurozoa), and Cassiopea xamachana (Scyphozoa)
Source: Gigascience. 2019 Jul 1;8(7):giz069. doi: 10.1093/gigascience/giz069 (PMC6599738; doi:10.1093/gigascience/giz069)

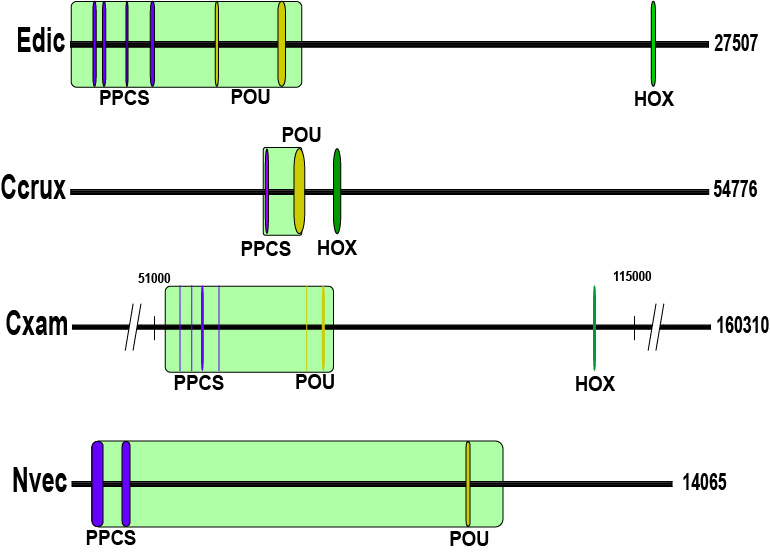

Supplement: giz069_Supplemental_Files [file giz069_supplemental_files.zip › Figure4.jpg]

Cluster plot

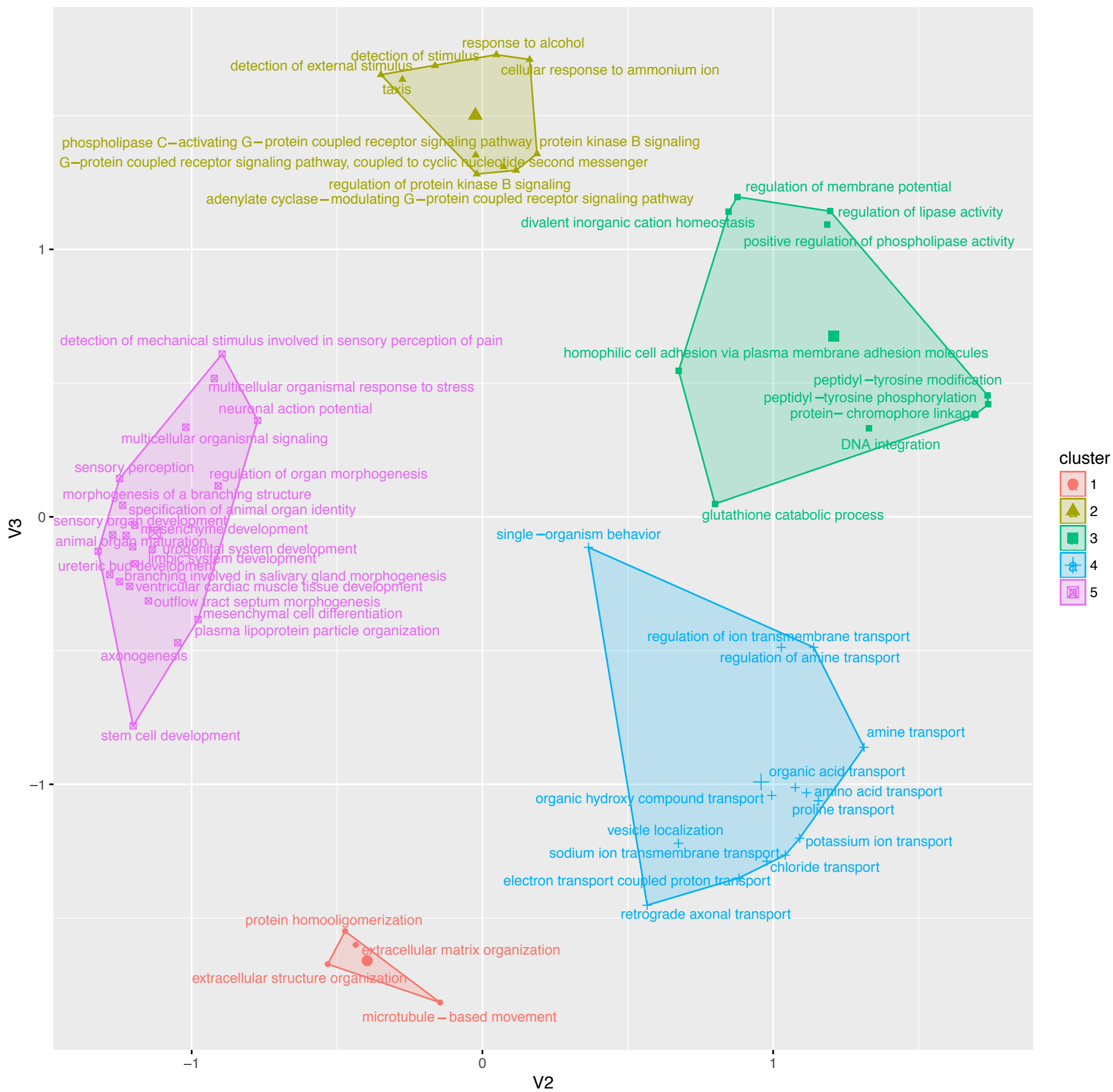

Supplement: giz069_Supplemental_Files [file giz069_supplemental_files.zip › Figure_S1.v2.pdf]

Cluster plot

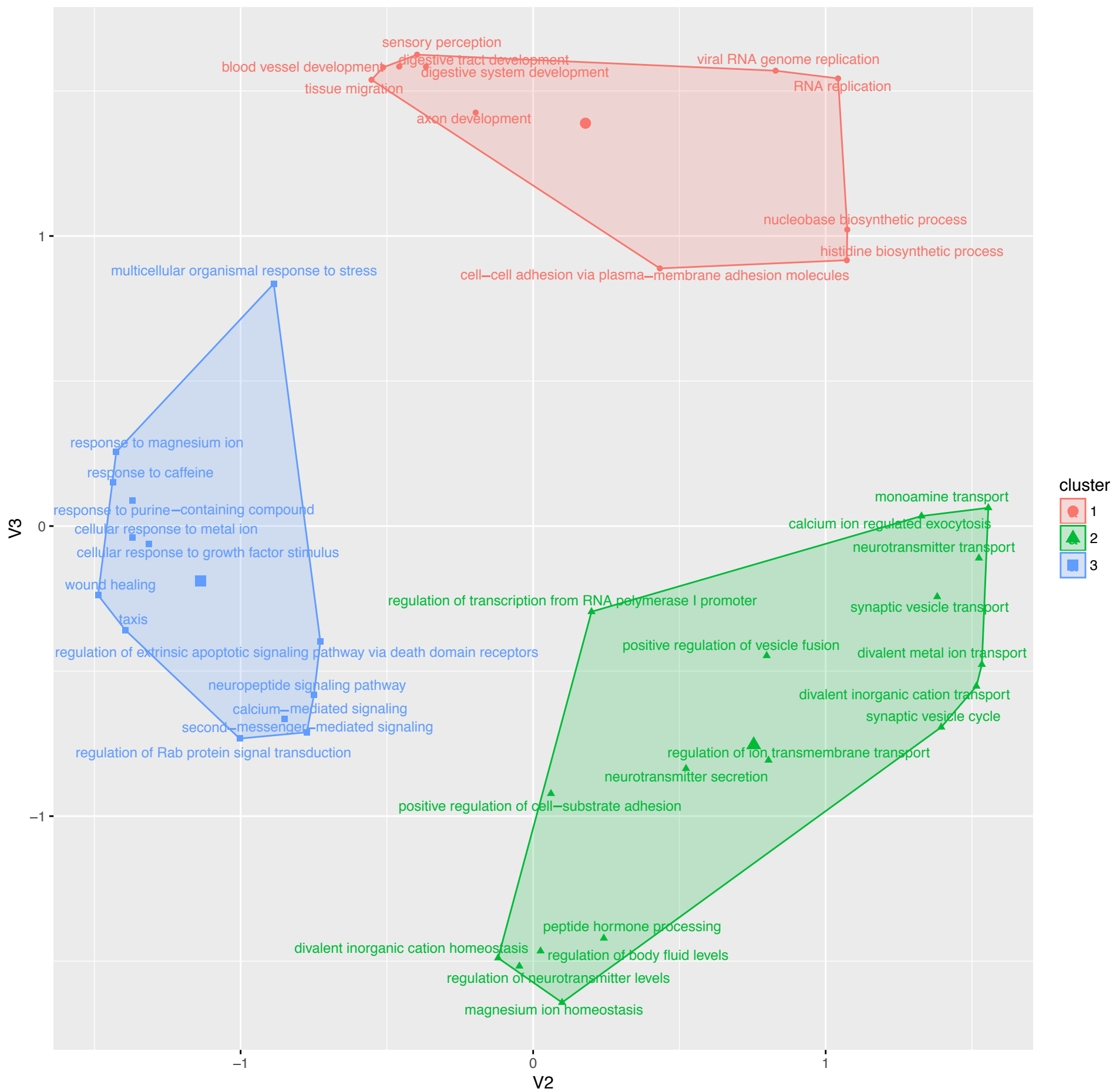

Supplement: giz069_Supplemental_Files [file giz069_supplemental_files.zip › Figure_S2.v2.pdf]
