# Supplementary material for: Box, stalked, and upside-down? Draft genomes from diverse jellyfish (Cnidaria, Acraspeda) lineages: Alatina alata (Cubozoa), Calvadosia cruxmelitensis (Staurozoa), and Cassiopea xamachana (Scyphozoa)
Source: Gigascience. 2019 Jul 1;8(7):giz069. doi: 10.1093/gigascience/giz069 (PMC6599738; doi:10.1093/gigascience/giz069)
Supplement: giz069_Response_to_Reviewer_Comments_Original_Submission [file giz069_response_to_reviewer_comments_original_submission.pdf]

Author's Response To Reviewer Comments

Close

Reviewer reports:
Reviewer #1: This is a straightforward report on the generation of 3 acraspedan genomes, the initial annotation steps and the potential further uses of the genomic resources. I believe the genomes were appropriately sequenced from carefully selected samples and I see no major issues with the manuscript at all. The genomes will be immensely important to understand not only patterns of evolution within Cnidaria but also to help clarify other issues in early-splitting lineages. I have only a couple of minor suggestions to the authors and a recommendation. But I believe this manuscript is almost ready for publication.

1. Please add the information about number of reads and volume of data for Calvadosia
- we've added this information to line 197

2. Their completeness analyses give relatively good numbers for Cassiopea and Calvadosia but very low recovery of eukaryotic and metazoan core genes in Alatina. I think the manuscript would benefit from a comment on why they think they recovered so little.
- we've added the following sentence on line 241
"The low recovery rates for conserved genes in the A. alata genome are likely due to the considerably larger size of the genome, which tends to be coupled with long introns, and therefore higher rates of gene fragmentation in a draft assembly [80-82].."

3. Although preliminary, the most interesting aspect of their analyses is the orthology. They detect potential orthologous groups that may represent lineage-specific gains, but they give no details about the genes themselves. Were they identifiable genes in terms of affinity with any other known gene? Could they identify conserved domains? I understand that would deserve a paper themselves, but I think the reader would appreciate more information to understand the importance of these resources.
- we've added some gene ontology analysis to give readers some context for the genes that were found (see figures 3-5, supplementary figures 1-2, and line 265-312. We agree with the reviewer that going into more detail is perhaps outside the scope of the giganote.

Reviewer #2: This manuscript provides a brief description of three draft genomes from medusozoan cnidarians. The genomic resources generated from this study will be very useful to the cnidarian research community. The paper is relatively straightforward, but it lacks a few details that could improve the quality of the contribution. My minor comments are listed below.

* The introduction could be improved by focusing more on the specific taxa (instead of all of cnidaria), the questions specifically addressed (venom composition, synteny), and the value of these data to the community (i.e. phylogenetic position, filling phylogenetic gaps), instead of a textbook-like introduction to Cnidaria.
We've added a paragraph which provides details of each taxa with references starting on line 119 - 130.

* The assembly methods for the three genomes needs to be standardized to include the same information for each of the genomes. For example, why were methods given for mit. Genome assembly for Calvadosia and not the others? Is this relevant to the paper?
The genomes were assembled by different team members at different times with different sequence data. Calvadosia was a particularly early assembly and at the time we were experimenting with different ideas on how to improve the assembly. One hypothesis was that the presence of mitochondrial reads, which can be present at much higher levels than nuclear genome, might improve kmer counting methods. This was fairly successful with Calvadosia, but had little effect with several other assemblies we attempted later. We therefore did not apply to Alatina and Cassiopea, and therefore did not assemble those mitochondrial genomes from our data. In addition, MT genomes from Alatina and Cassiopea have been described previously (https://academic.oup.com/gbe/article/4/1/1/536978#89413791). We have

added a more brief justification to the text starting on line 141 describing why the methodology is different for each genome.

* Lines 177 and 210 - were the units for N50 supposed to be kb or was this meant to be bp? What about the N50 unit for Alatina in line 229 (kb or bp?). Also, add the unit to the Table 1.
- The units were supposed to be bp. We have corrected this error, and they should now all have bp as the units.

* More detail for the Alatina assembly is needed. How were the Illumina and PacBio reads treated and combined?
- The Alatina reads were assembled by Masurca, which instructs users not to pre-process reads (in the manual). Masurca takes in both long and short-reads for the final assembly. We have added the following sentence starting on line 141: “These three genomes were sequenced over a five-year period, each by different authors, using various technologies and various assembly methods. As such, the methods for each assembly are unique.”

The output of Masurca included a fair amount of adapter that was caught by NCBI during sequence submission. We now mention this in the manuscript starting on line 230: "We conducted hybrid assembly of Illumina short-reads and PacBio long-reads using MaSuRCA 3.2.2 [79] (which includes an error correction step for paired-end reads) that resulted in an assembly of 291,445 contigs and an N50 of 7,049 bp (NCBI Accession PUGI00000000). We did not perform adapter trimming prior to assembly because the MaSuRCA manual advises against preprocessing of reads, including adapter removal. Nevertheless, we identified considerable adapter contamination in our final assembly."

* Some justification for the assembly methods would be warranted. Why was PacBio performed for Alatina and not the others? And how did (or didn't) PacBio affect the scaffolding? Why was artificial MP performed on Calvadosia and not the others? How did this affect assembly?
- The differences in assembly methods are justified now by the statement explaining that sequencing and assembly were performed by different authors. We feel that each of the assembly methods applied are sound and that we do a thorough job of describing each method. We also feel that analyzing these genomes together is worth the slight awkwardness of the assembly-method heterogeneity.

* A general discussion comparing assembly depth, scaffold # and size and genome completeness of all three genomes would be helpful. What sequencing, assembly methods and scaffolding proved most useful?
- We have added a section “Comparison of Assemblies” starting on line 245.

* Table 2 should be summarized into GO pie-charts or equivalent.
- We have moved Table 2 to the supplement and have added Figure 6

* Figure 2 is a bit difficult to read. It would be helpful it a Venn diagram accompanied this figure showing Cnidaria-specific, Medusozoan-specific and Acraspeda-specific orthologous groups.
- A six-way comparison will be a challenge to digest in a venn or in the current format. We have added color to group-specific combinations for visual clarification for Figures 2 and 7.

* An estimate of genomes sizes would be useful information to include.
- We now include “estimated genome size” based on the k-mer-based estimates produce by AllPaths-LG in Table 1.

* Line 312 - the 630-850 million year origin for Medusozoan is not a commonly held estimate. Please use a more reasonable estimate and citation.
- We have changed this to “likely lived more than 500 million years.”

* Line 344 - change specious to species-rich (specious means something else).
- done.

* Please cite other recent papers (in addition to Kayal et al, 2018) on cnidarian phylogenomics that contributed much of the data from Kayal et al., 2018.
- We now mention Chang et al. (2015) and Zapata et al. (2015) on line 65.

Close
