# Supplementary material for: Box, stalked, and upside-down? Draft genomes from diverse jellyfish (Cnidaria, Acraspeda) lineages: Alatina alata (Cubozoa), Calvadosia cruxmelitensis (Staurozoa), and Cassiopea xamachana (Scyphozoa)
Source: Gigascience. 2019 Jul 1;8(7):giz069. doi: 10.1093/gigascience/giz069 (PMC6599738; doi:10.1093/gigascience/giz069)
Supplement: giz069_Response_to_Reviewer_Comments_Revision_1 [file giz069_response_to_reviewer_comments_revision_1.pdf]

Author's Response To Reviewer Comments

Close

We've added citations for the three recently published medusozoan genomes from Leclere et al. 2019, Khalturin et al. 2019, and Kim et al. 2019. In addition, lines in the abstract and introduction referring to the lack of medusozoan genomes have been modified (line 42, line 22.

We removed URLs and replaced with citations for the FastqSifter and MateMaker, in addition to generating RRIDs for both tools. In addition, a place holder for the GigaScience data has been included at line 797 (reference #110).

Abbreviations, consent for publication, and Competing interests have been added after the Conclusions section.

Finally, some updates to co-author affiliations have been made.

Close
