# Supplementary material for: Box, stalked, and upside-down? Draft genomes from diverse jellyfish (Cnidaria, Acraspeda) lineages: Alatina alata (Cubozoa), Calvadosia cruxmelitensis (Staurozoa), and Cassiopea xamachana (Scyphozoa)
Source: Gigascience. 2019 Jul 1;8(7):giz069. doi: 10.1093/gigascience/giz069 (PMC6599738; doi:10.1093/gigascience/giz069)
Supplement: giz069_Reviewer_1_Report_Original_Submission — Ana Riesgo, PhD -- 6/4/2018 Reviewed [file giz069_reviewer_1_report_original_submission.pdf]

Reviewer Report

Title: Box, stalked and upside-down? Draft genomes from diverse jellyfish (Cnidaria, Acraspeda) lineages: Alatina alata (Cubozoa), Calvadosia cruxmelitensis (Staurozoa), and Cassiopea xamachana (Scyphozoa)

Version: Original Submission **Date:** 6/4/2018

Reviewer name: Ana Riesgo, PhD

Reviewer Comments to Author:

This is a straightforward report on the generation of 3 acraspedan genomes, the initial annotation steps and the potential further uses of the genomic resources. I believe the genomes were appropriately sequenced from carefully selected samples and I see no major issues with the manuscript at all. The genomes will be immensely important to understand not only patterns of evolution within Cnidaria but also to help clarify other issues in early-splitting lineages. I have only a couple of minor suggestions to the authors and a recommendation. But I believe this manuscript is almost ready for publication.
1. Please add the information about number of reads and volume of data for Calvadosia
2. Their completeness analyses give relatively good numbers for Cassiopea and Calvadosia but very low recovery of eukaryotic and metazoan core genes in Alatina. I think the manuscript would benefit from a comment on why they think they recovered so little.
3. Although preliminar, the most interesting aspect of their analyses is the orthology. They detect potential orthologous groups that may represent lineage-specific gains, but they give no details about the genes themselves. Were they identifiable genes in terms of affinity with any other known gene? Could they identify conserved domains? I understand that would deserve a paper themselves, but I think the reader would appreciate more information to understand the importance of these resources.

Level of Interest

Please indicate how interesting you found the manuscript: Choose an item.

Quality of Written English

Please indicate the quality of language in the manuscript: Choose an item.

Declaration of Competing Interests

Please complete a declaration of competing interests, considering the following questions:

- Have you in the past five years received reimbursements, fees, funding, or salary from an organisation that may in any way gain or lose financially from the publication of this manuscript, either now or in the future?

- Do you hold any stocks or shares in an organisation that may in any way gain or lose financially from the publication of this manuscript, either now or in the future?
- Do you hold or are you currently applying for any patents relating to the content of the manuscript?
- Have you received reimbursements, fees, funding, or salary from an organization that holds or has applied for patents relating to the content of the manuscript?
- Do you have any other financial competing interests?
- Do you have any non-financial competing interests in relation to this paper?

If you can answer no to all of the above, write 'I declare that I have no competing interests' below. If your reply is yes to any, please give details below.

I declare that I have no competing interests

I agree to the open peer review policy of the journal. I understand that my name will be included on my report to the authors and, if the manuscript is accepted for publication, my named report including any attachments I upload will be posted on the website along with the authors' responses. I agree for my report to be made available under an Open Access Creative Commons CC-BY license (http://creativecommons.org/licenses/by/4.0/). I understand that any comments which I do not wish to be included in my named report can be included as confidential comments to the editors, which will not be published.

I agree to the open peer review policy of the journal

To further support our reviewers, we have joined with Publons, where you can gain additional credit to further highlight your hard work (see: https://publons.com/journal/530/gigascience). On publication of this paper, your review will be automatically added to Publons, you can then choose whether or not to claim your Publons credit. I understand this statement.

Yes Choose an item.
