# Supplementary material for: Box, stalked, and upside-down? Draft genomes from diverse jellyfish (Cnidaria, Acraspeda) lineages: Alatina alata (Cubozoa), Calvadosia cruxmelitensis (Staurozoa), and Cassiopea xamachana (Scyphozoa)
Source: Gigascience. 2019 Jul 1;8(7):giz069. doi: 10.1093/gigascience/giz069 (PMC6599738; doi:10.1093/gigascience/giz069)
Supplement: giz069_Reviewer_2_Report_Original_Submission — Paulyn Cartwright -- 6/4/2018 Reviewed [file giz069_reviewer_2_report_original_submission.pdf]

Reviewer Report

Title: Box, stalked and upside-down? Draft genomes from diverse jellyfish (Cnidaria, Acraspeda) lineages: Alatina alata (Cubozoa), Calvadosia cruxmelitensis (Staurozoa), and Cassiopea xamachana (Scyphozoa)

Version: Original Submission **Date:** 6/4/2018

Reviewer name: Paulyn Cartwright

Reviewer Comments to Author:

This manuscript provides a brief description of three draft genomes from medusozoan cnidarians. The genomic resources generated from this study will be very useful to the cnidarian research community. The paper is relatively straightforward, but it lacks a few details that could improve the quality of the contribution. My minor comments are listed below.
* The introduction could be improved by focusing more on the specific taxa (instead of all of cnidaria), the questions specifically addressed (venom composition, synteny), and the value of these data to the community (i.e. phylogenetic position, filling phylogenetic gaps), instead of a textbook-like introduction to Cnidaria.
* The assembly methods for the three genomes needs to be standardized to include the same information for each of the genomes. For example, why were methods given for mit. Genome assembly for Calvadosia and not the others? Is this relevant to the paper?
* Lines 177 and 210 - were the units for N50 supposed to be kb or was this meant to be bp? What about the N50 unit for Alatina in line 229 (kb or bp?). Also, add the unit to the Table 1.
* More detail for the Alatina assembly is needed. How were the Illumina and PacBio reads treated and combined?
* Some justification for the assembly methods would be warranted. Why was PacBio performed for Alatina and not the others? And how did (or didn't) PacBio affect the scaffolding? Why was artificial MP performed on Calvadosia and not the others? How did this affect assembly?
* A general discussion comparing assembly depth, scaffold # and size and genome completeness of all three genomes would be helpful. What sequencing, assembly methods and scaffolding proved most useful?
* Table 2 should be summarized into GO pie-charts or equivalent.
* Figure 2 is a bit difficult to read. It would be helpful it a Venn diagram accompanied this figure showing Cnidaria-specific, Medusozoan-specific and Acraspeda-specific orthologous groups.
* An estimate of genomes sizes would be useful information to include.
* Line 312 - the 630-850 million year origin for Medusozoan is not a commonly held estimate. Please use a more reasonable estimate and citation.
* Line 344 - change specious to species-rich (specious means something else).
* Please cite other recent papers (in addition to Kayal et al, 2018) on cnidarian phylogenomics that contributed much of the data from Kayal et al., 2018.

Level of Interest

Please indicate how interesting you found the manuscript: Choose an item.

Quality of Written English

Please indicate the quality of language in the manuscript: Choose an item.

Declaration of Competing Interests

Please complete a declaration of competing interests, considering the following questions:

- Have you in the past five years received reimbursements, fees, funding, or salary from an organisation that may in any way gain or lose financially from the publication of this manuscript, either now or in the future?
- Do you hold any stocks or shares in an organisation that may in any way gain or lose financially from the publication of this manuscript, either now or in the future?
- Do you hold or are you currently applying for any patents relating to the content of the manuscript?
- Have you received reimbursements, fees, funding, or salary from an organization that holds or has applied for patents relating to the content of the manuscript?
- Do you have any other financial competing interests?
- Do you have any non-financial competing interests in relation to this paper?

If you can answer no to all of the above, write 'I declare that I have no competing interests' below. If your reply is yes to any, please give details below.

No to all of the above.

I agree to the open peer review policy of the journal. I understand that my name will be included on my report to the authors and, if the manuscript is accepted for publication, my named report including any attachments I upload will be posted on the website along with the authors' responses. I agree for my report to be made available under an Open Access Creative Commons CC-BY license (http://creativecommons.org/licenses/by/4.0/). I understand that any comments which I do not wish to be included in my named report can be included as confidential comments to the editors, which will not be published.

I agree to the open peer review policy of the journal

To further support our reviewers, we have joined with Publons, where you can gain additional credit to further highlight your hard work (see: https://publons.com/journal/530/gigascience). On publication of this paper, your review will be automatically added to Publons, you can then choose whether or not to claim your Publons credit. I understand this statement.

Yes Choose an item.
